# A moderated mediation model in assessing links between rumination, emotional reactivity, and suicidal risk in alcohol use disorder

**DOI:** 10.3389/fpsyt.2025.1479827

**Published:** 2025-02-28

**Authors:** Mateusz Wojtczak, Karol Karasiewicz, Katarzyna Kucharska

**Affiliations:** ^1^ Institute of Psychology, Cardinal Stefan Wyszynski University, Warsaw, Poland; ^2^ Instutute of Psychology, University of Szczecin, Szczecin, Poland

**Keywords:** alcohol use disorder, ruminations, emotional reactivity, suicide risk, negative affect

## Abstract

**Introduction:**

Suicide is a major public health concern, particularly among people with alcohol use disorders (AUD). Rumination, as a dysfunctional emotion regulation strategy, and increased emotional reactivity may significantly influence suicide risk in this population.

**Aim:**

The aim of this study was to assess whether different emotional reactivity mediate the association between ruminations and suicide risk, and whether AUD or control group (HC) status moderates these relationships.

**Methods:**

A study was conducted with 152 participants, including 86 from AUD and 66 from HC. Self-report questionnaires measuring ruminations, emotional reactivity and suicide risk were used. Structural Equation Modeling, invariance analysis, and moderated mediation estimation were used in the analyses.

**Results:**

The mediation analysis in the full sample revealed a significant indirect effect of rumination on suicide risk via emotional reactivity. Multi-group analysis indicated no significant differences in the mediation effect between the AUD and HC groups, with neither group showing a statistically significant indirect effect.

**Conclusions:**

The findings indicate that emotional reactivity may serve as a key mechanism mediating the relationship between rumination and suicide risk. Therapeutic interventions should focus on reducing ruminations and emotion reactivity to effectively reduce suicide risk in this group. Further research is needed to better understand these mechanisms.

## 1 Introduction

Suicide is one of the leading causes of death in the modern world. Each year, more than 800 000 people lose their lives to suicide as a result of a variety of circumstances (1). The theoretical construct of suicide is characterised by complexity and stages. The different stages of suicidal behaviour can be divided into suicidal thoughts, plans and attempts (2). Suicidal thoughts or ideation, is a broad term used to describe a range of ideas, fantasies or obsessions about ending one’s life (3). Suicide plans are mental representations of ways and strategies of action intended to lead to the successful realisation of an intention to take one’s own life. A suicide attempt, on the other hand, is a behaviour that leads to significant harm or a real threat to life (4).

Undoubtedly, one type of mental disorder that has a significant association with increased suicide risk is substance abuse. It has been confirmed in the literature that alcohol use disorders (hereafter AUD) increase suicide risk (5), and AUD with co-occurring mood disorders (6) represents the main psychiatric diagnosis for completed suicide attempts (7). Consumption of significant amounts of alcohol often immediately precedes the onset of suicidal thoughts and attempts, and analyses of *post-mortem* toxicological findings confirm the presence of alcohol in the blood among 30% of suicidal individuals (8).

Theoretical conceptions of the mechanisms underlying the predictive relationship between AUD and suicide risk include two complementary patterns: a proximal relationship due to acute alcohol intoxication and a distal (i.e. predisposing) relationship due to chronic alcohol use (9). The effects of alcohol intoxication, e.g. increased dysphoria, psychomotor agitation, impaired perception and impaired consciousness, can cause behavioural and affective disinhibition potentially reducing the fear of death that might otherwise act as a psychological barrier to suicide (10). In contrast, in the case of chronic alcohol use, the mechanisms of influence on suicide risk are more complex and involve both behavioural and neural processes related to the long-term effects of alcohol on brain structure and function.

People with a history of suicidal behaviour and those with AUD show similar changes in brain morphometry. According to neuroimaging studies, individuals with alcohol dependence experience impairments in higher cognitive functions, including inhibitory control and decision-making, which have been linked to structural changes in the prefrontal cortex area (PFC), including a significant reduction in grey matter volume in the dorsolateral prefrontal cortex (DLPFC) (11), medial (mPFC) (12), as well as orbitofrontal cortex (OFC) (13). Suicidal behaviour among individuals with a history of suicide is also associated with a reduction in grey matter volume in the PFC, particularly in the dlPFC involved in decision-making and inhibitory control (14–16).

Difficulties caused by emotional overreactions in response to negative stimuli and associated emotion dysregulation among people with AUD have been linked by researchers to increased activation of amygdala (17) as well as reduced amygdala volume (18). Similar results were obtained in a population of individuals with a history of suicidal thoughts, behaviours and attempts, where significant reductions in the grey matter in the amygdala were also found (19) and greater activation of this area during fMRI tasks (1).

Dysfunctional changes in the area of the reward system and habits in the AUD group have been linked to increased activity of nucleus accumbens (20) and decreased volume of the nucleus accumbens (21). In addition, studies have shown a significant reduction in the volume of both the caudate nucleus (22) and the putamen (23) in the AUD group, which have been linked to increased symptoms of alcohol dependence, impulse control disorders and increased risk of relapse (24, 25). Similarly, greater putamen activity has been noted among those with suicide attempts (26) and reduced grey matter volume in both putamen and caudate nucleus area (27).

One transdiagnostic risk factor for suicide is emotional dysregulation resulting from the use of maladaptive emotion regulation strategies (28, 29). In the research literature, rumination is defined as a dysfunctional emotion regulation strategy involving a repetitive and passive focus on symptoms of distress, as well as on the possible causes and consequences of these symptoms (30). Rumination is expressed by the presence of content-independent perseverative and/or fixational thoughts that result in a lack of problem solving (30) and thus leads to hyperactivation of emotional reactions (31). Research indicates that ruminations (measured as a state) are associated with increased HPA axis activity, resulting in higher levels of cortisol, the main stress hormone, although results for ruminations (measured as a trait) are less conclusive (32, 33). Furthermore, ruminations affect the sympathetic nervous system, as reflected in increased heart rate (HR) and blood pressure (BP) during stress-inducing fMRI tasks (34). Ruminations are associated with structural changes and brain activation. Research indicates that individuals with high levels of ruminations have altered volume in the DLPFC and anterior cingulate cortex (ACC), which affects their ability to regulate emotions and make decisions (35). fMRI studies have shown that ruminations are associated with impaired activation of the default mode network (36). The results confirm that rumination is an important factor in problematic alcohol use (37) and highlight the potential importance of therapeutic interventions targeting the mechanisms of ruminative strategies to treat alcohol dependence (38). Both cognitively (rumination) and interpersonally (co-rumination), the ruminative strategy is significantly associated with increased alcohol use in both adolescents and adults regardless of gender (39). In addition, individuals using ruminative strategies may similarly use alcohol to escape from or modulate distressing thoughts (39). The authors also showed that ruminations can directly or indirectly cause cognitive hunger experiences when an individual uses alcohol to interrupt the ruminative process, with the likelihood of relapse increasing due to the involvement of alcohol-dependent individuals in ruminations (40).

Empirical findings suggest that ruminations may mediate the impact of negative life events on suicidal thoughts (41). The authors highlight that rumination is positively related to suicidal thoughts and the likelihood of suicide attempts, through the maintenance of negative emotions and accompanying dysfunctional cognitive schemas (42). Rumination has been shown to be a predictor of the duration of suicidal thoughts (43). Furthermore, specific ruminations about suicide, i.e. repeated thoughts about suicide, have been shown to be associated with a higher prediction of a suicide attempt than other known risk factors (44) and may mediate the association between suicidal thoughts and suicide attempts across the life course (45). This scientific domain shows particular clinical potential in a population with AUD, due to the limited number of studies directly analysing the association between ruminations and suicide risk in this group. Undoubtedly, there is a need for further empirical exploration regarding this research issue and understanding complexity of mechanisms may contribute to the development of more effective therapeutic interventions.

Because rumination can lead to emotional arousal, it has been linked to increased emotional reactivity (46, 47). Furthermore, research suggests that rumination may prolong emotional reactivity following a negative event (32). Ruminations have been shown to correlate positively with emotional reactivity, particularly in the context of elevated cortisol levels and increased negative affect. This suggests a link between ruminations and physiological and behavioural markers of emotional reactivity (48).

Analyses of emotional reactivity as a potential risk factor for suicide have appeared in the literature (49). Researchers define emotional reactivity as a subjective predisposition to respond intensely to a wide range of emotional stimuli (especially of negative valence) and to recover slowly from emotional arousal (50, 51). Furthermore, the authors describe emotional reactivity in terms of emotion sensitivity/activation (e.g., speed of response to stimuli), emotion intensity (e.g., how strongly or intensely the emotion is felt) and emotion persistence (e.g., how long it takes to return to baseline after arousal) (51). Emotional reactivity has been linked to increased activation of the autonomic system and the HPA axis (48). Neuroimaging studies have shown that high emotional reactivity correlates with increased activation of the amygdala as well as the left PFC (52).

Among people with AUD, the accumulation of negative life events leads to an increase in negative emotional states (53). According to theories of emotional reactivity, individuals characterised by experiencing emotions with greater intensity may also have difficulty tolerating stressful situations (54). Therefore, the risk of AUD is high in individuals with high levels of emotional reactivity (55).

Theoretical models of suicide assume that along with a high level of emotional reactivity, there is significant clinical suffering that the individual cannot cope with, and suicide is a behavior aimed at escaping mental suffering (56). Also in the theoretical motivational-volitional model of suicide (57) emotional reactivity has been identified as one of the diatheses providing the background for the occurrence of suicidal thoughts. Similarly, empirical research indicates that there is a positive association between emotional reactivity and suicidal thoughts and attempts (58, 59). Furthermore, current research indicates higher levels of emotional reactivity among people with emotion dysregulation disorders, including patients with suicidal tendencies (60). However, the existing research literature on the relationship between suicide risk and emotional reactivity lacks empirical data that takes into account the context of maladaptive emotion regulation strategies, including ruminations. Also, the mediating mechanisms (i.e. how emotional reactivity relates to suicide risk) and moderating mechanisms underlying the relationship remain largely unexplored.

Research on the direct impact of rumination on suicide risk remains limited both among individuals with AUD and within the healthy control (HC) group. It has been demonstrated that rumination affects emotional reactivity, which may serve as a mediating variable (mediator) in the relationship between rumination and suicide risk. Consequently, modelling the indirect pathway involving emotional reactivity as a mediator warrants further investigation, particularly in AUD and HC groups, given the lack of prior studies addressing this associations.

The aim of the present study is to assess: a) intergroup differences in levels of ruminations, emotional reactivity (emotions with negative valence) and suicide risk, b) correlations between study variables, c) predictive structural model designed to examine the relationships between rumination, emotional reactivity, and suicide risk in two study groups: individuals with AUD and HC. The model posits that rumination has a direct effect on suicide risk, while emotional reactivity serves as a mediating variable in the relationship between rumination and suicide risk. Furthermore, group status (AUD vs. HC) moderates these relationships. Additionally, the analysis aimed to assess the invariance of the model across the two groups by examining intergroup differences in the structural relationships between the tested variables.

## 2 Materials and methods

### 2.1 Participants

All together 152 participants took part in the study, which was conducted anonymously online via the study website. Data came from an ongoing project assessing emotion dynamics and regulation in the context of suicide risk factors and protective factors in people with a diagnosis of AUD compared to HC.

The AUD sample consisted of *n* = 86 subjects aged between 24 and 67 years (*M_age_* = 39.15; *SD_age_* = 8.82), among whom *n_m_* = 54 were men (62.8%) and *n_w_* = 32 were women (37.2%). The majority of AUD respondents (46.5%) had a secondary education. Completed primary education was declared by 1.2% of people. Vocational school was completed by 8.1% of the respondents, while tertiary education was held by 38.4% of the respondents. Most people (67.4%) did not declare somatic diseases. The mean duration of abstinence in years among AUD subjects was *M* = 2.17, *SD* = 4.09. The AUD group was recruited from among those maintaining abstinence in inpatient wards and outpatient treatment addiction clinics. Inpatient treatment included an 8-week abstinence-based programme and intensive individual and group therapy. In contrast, outpatient treatment included patients in both partial and full remission. The diagnosis of AUD was based on the International Classification of Diseases and Health Problems (61) psychiatric diagnosis made on admission to the treatment unit. Due to the high overrepresentation of men in addiction treatment programmes, the AUD group in this study was predominantly male (62.8%). Patients were ineligible to participate in a treatment programme if they had a clinically significant cognitive deficit or met any of the following criteria: a history of psychosis, a co-occurring psychiatric disorder requiring current psychiatric treatment (e.g. history of psychosis, bipolar affective disorder) or the presence of acute alcohol withdrawal symptoms.

The HC group consisted of *n* = 66 people aged 18 to 57 years (*M_age_* = 29.35*; SD_age_* = 9.46), of whom *n_m_* = 37 were men (56.1%) and *n_k_* = 29 were women (43.9%). Exclusion criteria for the HC group was the presence of an alcohol related problem and other exclusion criteria were similar to those for the AUD group. The majority of subjects in the HC group (56.1%) had a tertiary education. Completed primary education was declared by 3.0% of subjects. Secondary school was completed by 22.7% of the respondents, while those currently studying accounted for 13.6% of the respondents.

The study was approved by the Ethics Committee at Cardinal Stefan Wyszynski University in Warsaw (Evidence: 6/2023).

### 2.2 Measures

Sociodemographic characteristics (e.g. age, biological sex, education) were obtained by means of a self-report questionnaire.

Audit (62). The tool is a screening tool to determine the extent to which a person surveyed consumes alcohol. It contains ten questions that address three areas of alcohol use: risky drinking, harmful drinking and alcohol dependence. Each question is assigned a set of answers to choose from. Each answer is assigned a score (from 1 to 4). Reliability for the total score Cronbach’s alpha = 0.972.

The Hospital Anxiety Depression Scale (HADS) (63), Polish validation (64). A screening tool used to assess the severity of medication and depression symptoms. It is not used for clinical diagnosis. It contains 14 items, of which 7 form the anxiety subscale (HADS-A) and 7 form the depression subscale. For the depression scale, Cronbach’s alpha reliability = 0.752.

Cognitive Emotion Regulation Questionnaire (CERQ) (65), Polish validation (66):. A 36-item questionnaire designed to assess individual differences in cognitive emotion regulation in response to stressful, threatening or traumatic life events. The tool assesses nine 4-item dimensions: blaming self, blaming others, acceptance, refocusing on planning, positive refocusing, rumination, positive refocusing, positive refocusing, perspective taking and catastrophising. Responses are given on a 5-point Likert scale from 1 ‘almost never’ to ‘almost always’. Therefore, subscale scores can range from 4 to 20, with higher subscale scores indicating greater frequency of use of a particular cognitive strategy. Cronbach’s alpha reliability for the ruminations scale = 0.763.

Perth Emotional Reactivity Scale (PERS) (67) Polish version (30): is a 30-item self-report questionnaire used to assess traits (dimensions) of emotional reactivity. PERS examines emotional reactivity as defined by Becerra (67); that is, it measures the typical ease/speed of activation, intensity and duration of emotional reactions, and allows the assessment of emotional reactivity in relation to positive (e.g. joy) and negative (e.g. sadness) emotions separately. Scores for the six subscales and the two composite subscales can be obtained by summing the respondent’s responses (i.e. the numbers to be circled on the 5-point response scale) for the respective items. The higher the score for the individual subscales and the two composite subscales, the higher the level of the dimensions of emotional reactivity; in other words, this means that the emotion in question can be more easily/quickly activated, more intense and lasts longer. Cronbach’s alpha reliability for PERS General Emotional Reactivity scale = 0.928.

Suicidal Behaviour Questionnaire Revised (SBQR) (68) pol. version (69). The questionnaire consists of four test items. The first three questions concern retrospective assessment: (1) “Have you ever thought about taking your own life or made an attempt to do so?”; (2) “How often have you had thoughts about taking your own life in the past year?”; (3) “Have you ever told someone about your intention to commit suicide or the likelihood that you might commit suicide”. The last, fourth question, concerns prospective assessment: (4) “How likely is it that one day you will attempt/attempt suicide?”. Responses are scored on a scale of 1 to 3 (questions 1-3) and 0 to 6 (question 4). Cronbach’s alpha reliability = 0.864.

### 2.3 Statistical methods

In the first step of the analysis, a series of plots were generated to assess the relationships between the dependent variable, the mediator, and other variables. Both linear and quadratic functions were fitted to the observed data to evaluate their adequacy. This procedure was conducted separately for the alcoholic and healthy groups to assess the consistency of the relationships within each group. The series of plots and R-squared coefficients for both linear and quadratic functions, included in **Supplementary Material A**, demonstrated that linear relationships between variables were comparable to, or slightly weaker than, those for the quadratic function. However, the better fit of the quadratic function may have resulted from the small sample size and the presence of outliers, which could have significantly influenced the fit of both models. The visualised relationships confirmed the similarity in the fit of linear and quadratic functions; therefore, linear analyses were chosen for further investigation.

In the second step of the analysis, outliers were identified and smoothed. This smoothing procedure was performed using the statistical software SZTOS (70), designed to detect outliers in linear regression models testing moderation effects. The moderation procedure was applied because the slope-invariance procedure is a generalisation of simple interaction analysis.

The first step of the smoothing algorithm involved the random selection of the dependent variable, independent variable, and moderating variable, followed by the construction of a regression model. The randomness in selecting these variables aimed to minimise bias in the sequence of data imputation. Based on the specified regression model, the following influence measures were computed for each observation: studentised residuals, leverage values, and Cook’s distance (71). Observations with elevated values for these indicators were flagged, and missing values were inserted into the dependent variable columns of the identified rows. These missing values were then imputed using the random forests technique, which leveraged all available dataset information to predict the missing values. Additionally, if there were more than two independent, dependent, or moderating variables, the smoothed data from the first iteration were carried forward to subsequent smoothing analyses.

As mentioned earlier, missing data imputation via the random forests method was carried out using the *missForest* package from the CRAN repository (72) in R. This non-parametric method applies the random forests algorithm (73) to optimise the prediction of missing values. The random forests method involves randomly selecting observations (with replacement) to generate multiple decision trees containing different variables and observations from the dataset. When aggregated, these trees enable either regression-based prediction (for quantitative variables) or classification-based voting (for qualitative variables). The missing data imputation process using the random forests method consists of three iterative steps:

1. Determining the quantity and type (qualitative vs. quantitative) of missing data.
2. Replacing missing values with the mean (for continuous data) or mode (for categorical data).
3. Performing regression- or classification-based prediction of missing values using the random forests algorithm.

The entire dataset was randomly used in the missing value prediction process, and these steps were repeated until the prediction or classification error was minimised.

The results of the analysis are presented in **Supplementary Material B**, which includes a series of figures and tables. Figure *a* displays a plot of influential observations, Figure *b* presents the moderated relationships between variables in the model recalculated using raw data, and Figure *c* shows the moderated relationships recalculated using smoothed data predicted via the random forests technique. The analysis was performed on the full dataset, which consisted of *N* = 152 observations.

**Figure 1:**
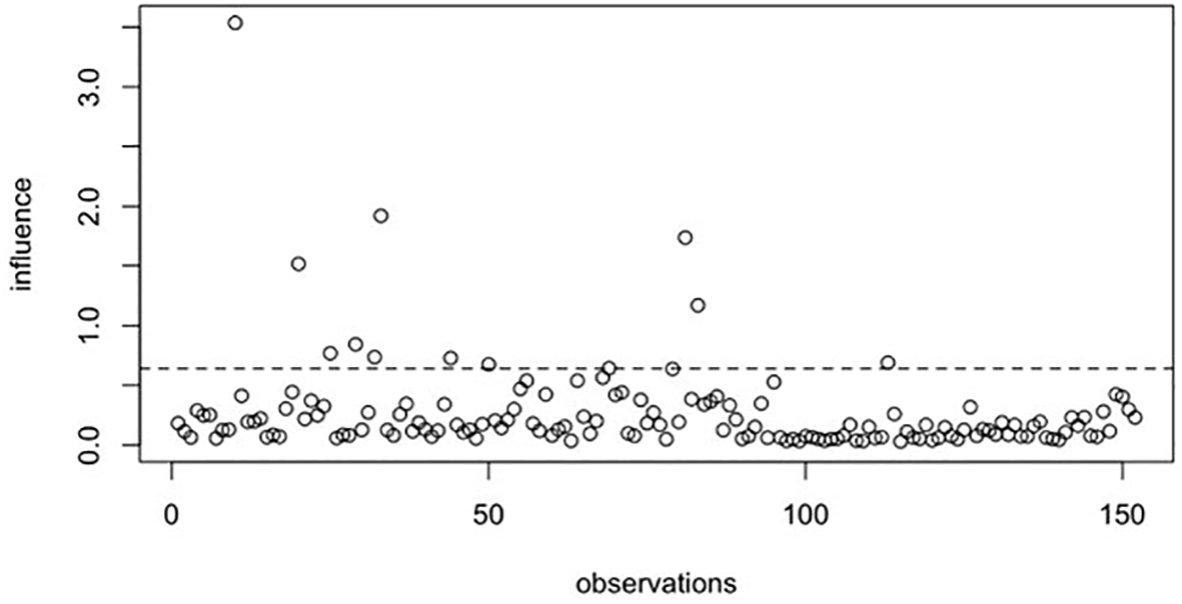
genCookDistance results based on raw data.

**Figure 2:**
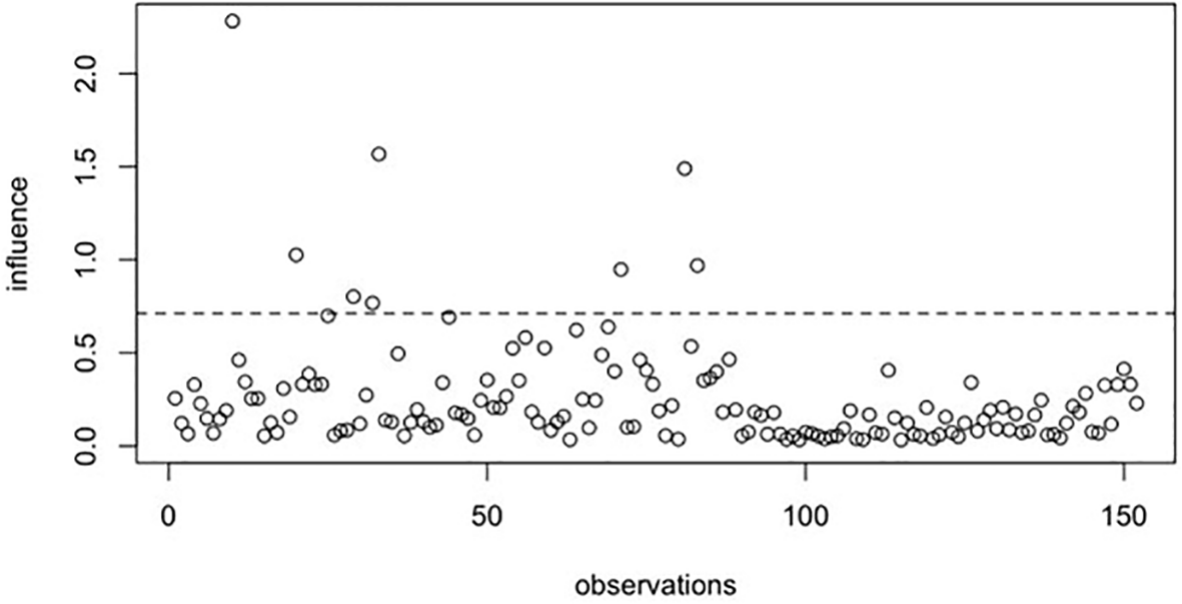
genCookDistance results based on preprocessed data.

Based on both the raw and smoothed data, two structural equation models were estimated using the *lavaan* package (74). The estimator used was MLR (maximum likelihood estimation with robust Huber-White standard errors), which is recommended for small samples with non-normally distributed data (75). Model diagnostics for both datasets were evaluated using general Cook’s Distance in the *influence.SEM* package (76). The *deltaChi* method was omitted because the model demonstrated an almost ideal fit. These diagnostic procedures iteratively removed each observation (one at a time) and refitted the SEM model to the reduced dataset (*N*-1 cases). The recalculated general Cook’s Distance indicated that preprocessing the data resulted in fewer extreme observations. Even if an observation remained an outlier in the smoothed dataset, its influence was weaker than in the raw data. The results of the Cook’s Distance analysis are presented in **Figures 1** and **2**.

To determine whether the groups were of equal size, a chi-square test for one variable was conducted (Pearson, 1900). The analysis revealed that the groups were statistically equal in size, χ² (1) = 2.63; p = 0.105. The homogeneity of variance in both groups was assessed using Levene’s test (Levene, 1960). If the assumption of homogeneity of variance was met, the result of Student’s t-test was reported; otherwise, Welch’s t-test was used (Welch, 1951). Effect sizes were assessed using Cohen’s d measure (Cohen, 2013). Analyses using Spearman’s correlation were performed to examine the relationship between the variables under study. A 95% confidence interval (CI) was used in both analyses.

Due to differences in the level of variables analysed across groups, and based on previous literature on the relationships examined, further analyses included the controlling co-variables: age (77), biological sex (47), current severity of alcohol use (78), severity of depressive symptoms (79, 80), and education (81).

## 3 Results

### 3.1 Preliminary analyses

First, intergroup differences were examined for the primary variables under investigation, namely rumination, emotional reactivity, and suicide risk. Subsequently, correlations between these variables were analysed both within the entire sample and across individual groups. Additionally, correlations between rumination, emotional reactivity, and suicide risk were assessed to evaluate the predictive validity of the structural model. The analyses also included depression severity, alcohol consumption, age, gender, and education as control variables. **Table 1** below presents the descriptive statistics for the analysed variables.

**Table 1:** Descriptive statistics for variables in the whole group (N=152).

Scale	*M*	*Min*	*Max*	*SD*	*S*	*K*	*W*	*p*
Age	34,89	18	67	10,30	,390	-,388	,971	**,003**
HADS Depression	6,05	0,00	18,00	4,25	,406	-,710	,947	**<,001**
AUDIT Score	11,16	0,00	40,00	13,58	,815	-1,01	,770	**<,001**
CERQ Rumination	8,78	0,00	16,00	3,04	-,363	-,123	,978	**,017**
PERS General Emotional Reactivity	31,62	0,00	59,00	12,10	-,070	-,631	,991	**,341**
SBQR Score	6,83	3,00	13,00	2,94	,410	-,940	,910	**<,001**

*M*, mean; *Min/Max*, minimum/maximum; *SD*, standard deviations; *S*, skewness; *K*, kurtosis; *W*, Shapiro-Wilk test Statistics; *p*, significance of the Shapiro-Wilk test. Bold values indicate statistical significance.

Individuals diagnosed with AUD had significantly higher mean scores across all the variables listed below compared to the control group, except for rumination. Specifically, the AUD sample attained a mean SBQR score of 7.81 ± 2.74. The authors of the scale proposed a cutoff score of 8 points for individuals in clinical samples (68). Detailed results are presented in **Table 2**.

**Table 2:** Student’s (Welch’s) t-test scores for variables in both groups (N=152).

	N (AUD)	M (SD)	N (HC)	M (SD)	t (df 150)	p	AUD vs HC	F	p (Levene)	T/W	Cohen’s d
PERS General Emotional Reactivity	86	35.12 (11.19)	66	27.06 (11.78)	4.3	< 0.001	AUD > HC	0.31	0.579	T	0.7
SBQR Score	86	7.81 (2.74)	66	4.58 (2.05)	8.33	< 0.001	AUD > HC	5.46	0.021	W	1.34
CERQ Rumination	86	9.19 (3.04)	66	8.26 (2.98)	1.88	0.062	AUD = HC	0.04	0.842	T	0.31
HADS Depression	86	6.74 (4.31)	66	5.15 (4.03)	2.32	0.021	AUD > HC	0.22	0.638	T	0.38
AUDIT Score	86	17.52 (14.98)	66	2.86 (3.39)	8.79	< 0.001	AUD > HC	252.24	< 0.001	W	1.35
AGE	86	39.15 (8.82)	66	29.35 (9.46)	6.58	< 0.001	AUD > HC	0.9	0.344	T	1.08

*M*, mean; *SD*, standard deviation; *t*, Student’s/Welch’s t-test statistic; *df*, Degrees of freedom; *p*, Statistical significance; *F*, Levene’s test for equality of variances; *T*, Assumption of equal variances met; *W*, Assumption of equal variances not met; Cohen’s *d*, Effect size statistic.

Correlations between rumination, emotional reactivity, suicide risk, and control variables were computed separately for individuals with AUD and HC to identify significant associations and preliminarily confirm the validity of the proposed model. **Table 3** presents the correlation coefficients for all variables in the entire sample, while **Tables 4** and **5** display the correlation coefficients within each group.

**Table 3:** rho Spearman correlation between variables in the whole group (N=152).

Scale	Id.	1	2	3	4	5	6	7	8
SBQR Score	1								
PERS Emotional Reactivity	2	0.46***							
HADS Depression	3	0.46***	0.36***						
CERQ Rumination	4	0.27**	0.33***	0.23**					
AUDIT Score	5	0.25**	0.13	0.19*	0.27**				
AGE	6	0.33***	0.01	-0.13	0.00	0.14			
SEX	7	-0.25**	-0.19*	-0.24**	-0.10	-0.15	0.04		
EDUCATION	8	0.21**	0.05	0.16*	0.10	0.11	0.10	-0.16	
GROUP	9	0.55***	0.34***	0.19*	0.17*	0.28***	0.49***	0.07	0.18*

* *p* < 0.05, ** *p* < 0.01, *** *p* < 0.001 (two-tailed).

**Table 4:** rho Spearman’s correlation between variables in the HC group (N=66).

Scale	Id.	1	2	3	4	5	6	7
SBQR Score	1							
PERS General Emotional Reactivity	2	0.34**						
HADS Depression	3	0.51***	0.33**					
CERQ Rumination	4	0.29*	0.54***	0.31*				
AUDIT Score	5	0.41**	0.13	-0.04	0.11			
AGE	6	0.07	-0.21	-0.29*	-0.15	0.27*		
SEX	7	-0.67***	-0.26*	-0.26*	-0.28*	-0.21	-0.13	
EDUCATION	8	0.29*	0.17	0.13	0.06	0.07	0.31*	-0.26*

*p < 0.05, ** *p* < 0.01, *** *p* < 0.001 (two-tailed).

**Table 5:** rho Spearman’s r correlation between variables in the AUD group (N=86).

SCALE	Id.	1	2	3	4	5	6	7
SBQR Score	1							
PERS General Emotional Reactivity	2	0.22*						
HADS Depression	3	0.37***	0.28**					
CERQ Rumination	4	0.12	0.11	0.13				
AUDIT Score	5	0.02	-0.05	0.23*	0.19			
AGE	6	0.08	-0.13	-0.23*	0.02	-0.17		
SEX	7	-0.13	-0.14	-0.25*	0.04	-0.19	0.22*	
EDUCATION	8	0.06	-0.13	0.12	0.07	0.11	-0.19	-0.11

* *p* < 0.05, ** *p* < 0.01, *** *p* < 0.001 (two-tailed).

### 3.2 Structural equation model, invariance analysis, and moderated mediation estimation

**Figure 3:**
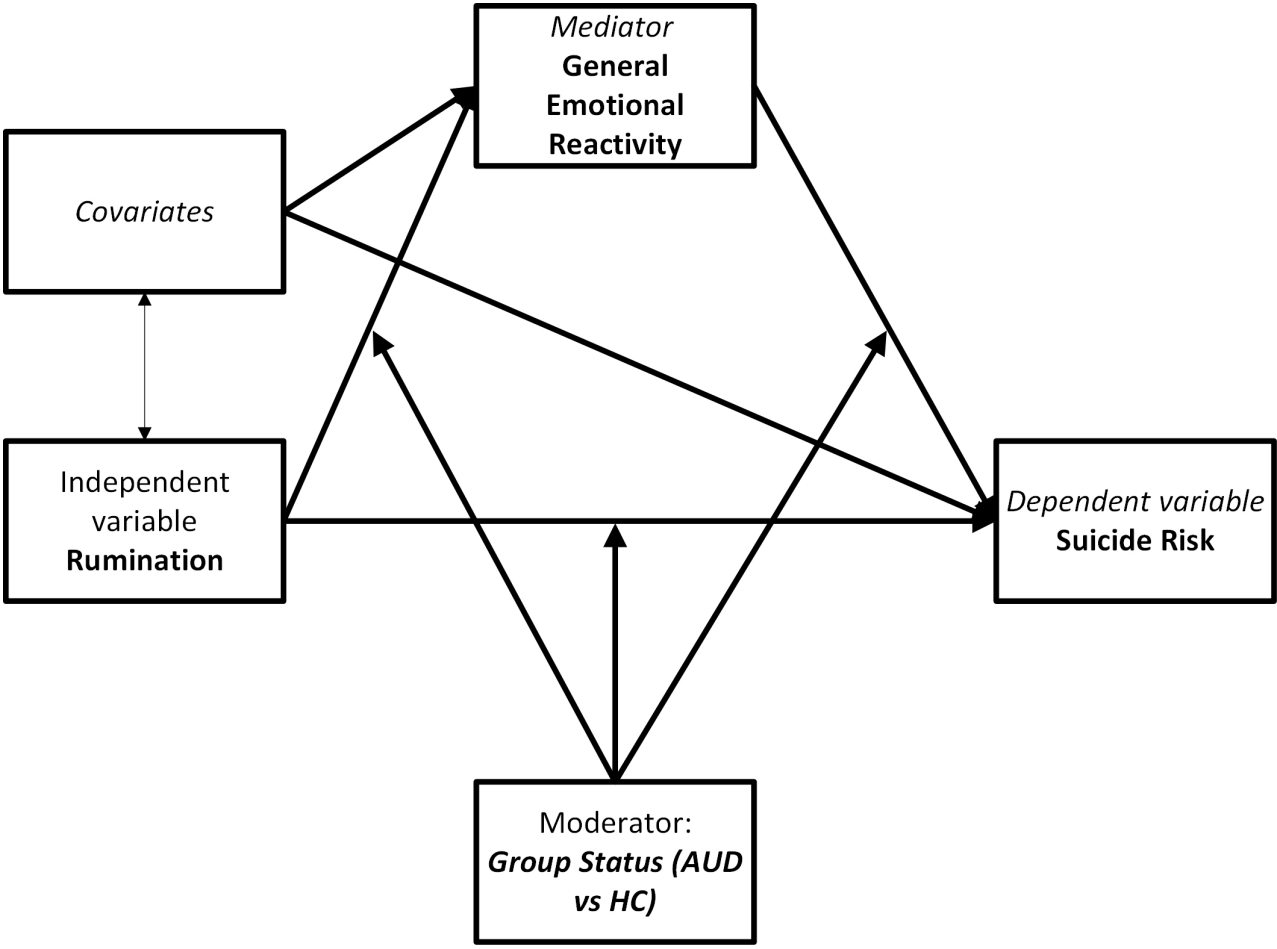
Conceptual structural equation model.

In this study, the relationship between rumination (CERQ Rumination) and suicide risk (SBQR Score) was examined, with emotional reactivity (PERS General Emotional Reactivity) included as a mediator (**Figure 3** presents conceptual model). The analysis was conducted using structural equation modelling (SEM) in R (lavaan package), and the estimation was performed using the MLR method (maximum likelihood estimation with robust standard errors), which provides robust estimates and adjusted standard errors.

The model fit analysis demonstrated a very good alignment between the model and the empirical data. The chi-square statistic for one degree of freedom was not statistically significant (χ²(1) = 0.01; *p* > 0.05), and other fit indices also indicated a high level of model-data fit (CFI = 1.00; TLI = 1.18; NFI = 1.00; IFI = 1.01; RMSEA = 0.00, 90% CI [0.00–0.07], PCLOSE = 0.942; SRMR = 0.00; GFI = 1.00; AGFI = 1.00). These results suggest that the model adequately reflects the structural relationships among the examined constructs. A detailed overview of all structural model parameter estimates is presented in **Table 6**.

**Table 6:** Estimated structural model results.

Dependent Variable	←	Independent Variable	*B*	s.e.	DPU	GPU	β	*Z*
SBQR Score	←	PERS General Emotional Reactivity	0.25	0.08	0.10	0.40	0.25	3.33**
SBQR Score	←	CERQ Rumination	0.07	0.06	-0.06	0.19	0.07	1.05
SBQR Score	←	HADS Depression	0.34	0.07	0.21	0.47	0.34	5.11***
SBQR Score	←	AUDIT Score	0.11	0.07	-0.04	0.25	0.11	1.45
SBQR Score	←	SEX	-0.16	0.14	-0.43	0.10	-0.08	-1.21
SBQR Score	←	AGE	0.03	0.01	0.02	0.04	0.33	5.68***
SBQR Score	←	EDUCATION	0.07	0.06	-0.04	0.17	0.07	1.18
PERS General Emotional Reactivity	←	CERQ Rumination	0.24	0.08	0.08	0.40	0.24	2.91**
PERS General Emotional Reactivity	←	HADS Depression	0.28	0.08	0.12	0.43	0.28	3.41**
PERS General Emotional Reactivity	←	AUDIT Score	0.06	0.08	-0.10	0.22	0.06	0.74
PERS General Emotional Reactivity	←	SEX	-0.23	0.16	-0.53	0.08	-0.11	-1.46
PERS General Emotional Reactivity	←	AGE	0.00	0.01	-0.01	0.02	0.04	0.50
PERS General Emotional Reactivity	←	EDUCATION	-0.07	0.06	-0.19	0.06	-0.08	-1.05

←, Direction of effect; B, Unstandardised regression coefficient; s.e., Standard error of B estimation; Z, Z statistic; LCI and UCI, 95% confidence intervals (lower and upper, respectively); β, Standardised regression coefficient; X²(1) = 0.01; p > 0.05; CFI = 1.00; TLI = 1.18; NFI = 1.00; IFI = 1.01; RMSEA = 0.00; 90% CI [0.00–0.07]; PCLOSE = 0.942; SRMR = 0.00; GFI = 1.00; AGFI = 1.00. *** p < 0.001, ** p < 0.01.

In the path analysis, the examined model accounted for the effect of rumination on suicide risk through a single mediator - emotional reactivity. The model also controlled for the effects of age, sex, education, depression severity (HADS Depression), and alcohol use severity (AUDIT Score). Additionally, correlations between rumination and control variables were estimated, allowing for the consideration of their potential influence on the analysed relationships (see **Table 1** in **Supplementary Material C**). A significant positive association was found between rumination and emotional reactivity (β = 0.24; Z = 2.91; *p* < 0.01), indicating that higher levels of rumination were significantly associated with heightened emotional reactivity. Furthermore, elevated emotional reactivity significantly increased suicide risk (β = 0.25; Z = 3.33; *p* < 0.001). However, the direct effect of rumination on suicide risk was not statistically significant (β = 0.07; Z = 1.05; *p* > 0.05), suggesting that this relationship primarily occurs via emotional reactivity.

The mediation effects in the full sample revealed a statistically significant indirect effect (b = 0.060; s.e. = 0.030; Z = 2.003; *p* = 0.045). This finding suggests that rumination contributes to increased suicide risk through heightened emotional reactivity. Furthermore, the lack of a significant direct effect of rumination on suicide risk strengthens the hypothesis of full (or predominant) mediation. The *R²* values indicated that the model explained 45% of the variance in suicide risk (*R²* = 0.45) and 21% of the variance in emotional reactivity (*R²* = 0.21), highlighting the model’s substantial predictive capability.

Further analysis of path coefficient invariance revealed significant differences in model fit between models assuming equal versus varying regression slopes across groups (χ²(13) = 36.82; *p* < 0.001). The lower χ² value suggests that the model accounting for group differences in specific paths provides a better fit to the data.

A multi-group analysis was conducted to examine the potential moderate mediation effect by group status (AUD vs. HC). **Table 7** presents the comparison of model parameter estimates between the AUD and HC groups. The overall mediation effect did not significantly differ between groups (Z = 1.05; *p* = 0.294). However, separate estimates of the indirect effect within each group indicated that in the HC group, the indirect effect (b = 0.048; s.e. = 0.032; Z = 1.493; *p* = 0.135) and in the AUD group (b = 0.010; s.e. = 0.017; Z = 0.585; *p* = 0.558) did not reach statistical significance.

**Table 7:** Comparison of model parameter estimates across AUD and HC groups defined by the GROUP variable.

Variables			AUD			HC			
DEPENDENT	←	INDEPENDENT	β	s.e.	*Z*	β	s.e.	*Z*	Zdiff
SBQR Score	←	PERS General Emotional Reactivity	0.12	0.11	1.08	0.17	0.10	1.73	-0.34
SBQR Score	←	CERQ Rumination	0.11	0.09	1.30	0.01	0.09	0.12	0.81
SBQR Score	←	HADS Depression	0.35	0.10	3.62***	0.39	0.09	4.56***	-0.30
SBQR Score	←	AUDIT Score	-0.07	0.10	-0.73	0.30	0.08	3.56***	-2.83**
SBQR Score	←	SEX	-0.07	0.11	-0.66	-0.38	0.11	-3.60***	2.00*
SBQR Score	←	AGE	0.19	0.09	2.06*	0.07	0.10	0.73	0.87
SBQR Score	←	EDUCATION	0.06	0.10	0.65	0.04	0.07	0.49	0.23
PERS General Emotional Reactivity	←	CERQ Rumination	0.09	0.11	0.84	0.40	0.10	4.05***	-2.11*
PERS General Emotional Reactivity	←	HADS Depression	0.28	0.10	2.67**	0.10	0.12	0.86	1.10
PERS General Emotional Reactivity	←	AUDIT	-0.12	0.10	-1.20	0.24	0.10	2.44*	-2.57*
PERS General Emotional Reactivity	←	SEX	-0.13	0.10	-1.29	-0.17	0.10	-1.72	0.31
PERS General Emotional Reactivity	←	AGE	-0.06	0.11	-0.53	-0.25	0.08	-2.99**	1.39
PERS General Emotional Reactivity	←	EDUCATION	-0.17	0.09	-1.89	0.02	0.11	0.16	-1.32

←, Direction of effect; β, Standardised regression coefficient; s.e., Standard error of β estimation; Z, Z statistic; Zdiff, Significance test for differences in β estimates between Group AUD and Group HC. *** p < 0.001, ** p < 0.01, *p < 0.05.

## 4 Discussion

The aim of this article was to examine the associations between rumination, emotional reactivity, and suicide risk (i.e., the presence of suicidal thoughts, plans, and attempts) in individuals with AUD and in a healthy control group. The analysis also included depressive symptoms and other sociodemographic variables (age, gender, education), as well as alcohol use severity. To the authors’ knowledge, this is the first study to use structural equation modelling (SEM) to estimate the influence of the analysed effects of rumination and emotional reactivity on suicide risk, as well as possible differences between the studied groups (AUD vs. HC). Therefore, in the next section of the article, we will compare our results with reports from other studies that analysed similar variables but in different contexts or groups.

In the tested model, in the entire studied sample, it turned out that rumination did not exert a significant direct effect on suicide risk. This is particularly interesting in the context of the research literature cited earlier, in which rumination was a predictor of suicide risk. However, our findings are consistent with other studies suggesting that rumination may operate through more complex mechanisms and mediators (82–85).

Our study confirmed that ruminations are significantly positively associated with emotional reactivity, and that increased emotional reactivity, in turn, significantly and positively affects suicide risk. Moreover, a significant mediation effect (b = 0.06, p = 0.045) of ruminations on suicide risk via emotional reactivity was obtained in the whole sample. This result is consistent with the concepts emphasising that intense and persistent ruminative states may lead to an increased susceptibility to reacting to emotions of negative valence. In turn, increased emotional reactivity facilitates the emergence and maintenance of suicidal thoughts. This sequence of events – from chronic, negative ruminations to the escalation of emotional reaction – may promote the development of suicidal behavior. It should also be noted that in our model, depressive symptoms turned out to be a key predictor of both emotional reactivity and suicidal risk, which confirms previous findings that depression is one of the most important risk factors in the context of suicidal behavior. suicidal.

The tested structural model explained approximately 45% of the variance in suicide risk and approximately 21% of the variance in emotional reactivity. These results are consistent with other reports indicating that cognitive factors (such as rumination) and emotional factors (including increased reactivity) combined with depressive symptoms may jointly contribute significantly to explaining the complex phenomenon of suicidal (49, 51, 86).

Invariance analysis showed that some of the regression slopes differed between the groups, which was confirmed by a significant difference in the fit of the models (χ²(13) = 36.82; p < 0.001). This means that some of the relationships between variables take slightly different values in both groups. For example, in the control group, the intensity of alcohol use turned out to be a significantly positive predictor of suicide risk, while in the AUD group this effect was insignificant and had a negative direction. On the other hand, in the AUD group, depression retained a significant, positive effect on suicide risk, which did not differ substantially from the effect in the control group, although the strength of the relationship may have differed slightly.

From the perspective of testing moderated mediation, the most important thing is that group status (AUD vs. HC) did not significantly change the indirect effect of rumination on suicide risk via emotional reactivity. In other words, the mechanism itself, in which ruminations translate into suicidal behaviours mainly due to increased emotional reactivity, did not depend in a statistically significant way on membership in the studied group. The effect obtained in the HC group is surprising in the context of another study, which showed that emotional reactivity was a significant predictor of suicidal thoughts and behaviours among people from the general population (59), however, in this study only the healthy population was assessed, and emotional reactivity was tested as an independent variable, and the authors did not control for depressive symptoms and current level of alcohol consumption as covariates.

Our result, however, may indicate that the analysed subgroups are too small to obtain the moderated mediation effect, as well as the significant mediation effect expected in the HC group, although our result suggests that some differences in the intensity of specific predictors (e.g. depression, alcohol use or gender) may modify this relationship in each of the groups. Similarly, Nolen-Hoeksema and Harrell (87) have shown that rumination is strongly associated with depression and perceived negative affect in alcohol abusers, which in the long term may lead to suicide risk. However, healthy individuals, in contrast to those with AUD, often have access to more adaptive emotion regulation strategies that allow them to effectively modulate negative emotion reactivity (88).

### 4.1 Clinical implications

These findings have important clinical implications. Therapeutic interventions and pharmacological treatment for people with AUD should specifically focus on reducing the severity of ruminative thoughts. Ruminations-focused interventions, such as metacognitive therapy and ruminations-focused cognitive behavioural therapy (89), have shown significant efficacy in the treatment of depression (90). In addition, there is some evidence to suggest that mindfulness-based cognitive therapy, which also places a strong emphasis on eliminating ruminative processes, is effective in therapy of patients with suicidal thoughts (91). More recently, forms of brief online interventions targeting ruminations have also emerged and have been found to be acceptable and effective in reducing symptoms of ruminations, worry anxiety and depression (92). For emotional reactivity, mindfulness meditation is a particularly promising intervention, as it has been found to simultaneously reduce physiological overreactivity and, in addition, reduce the severity of ruminations (93). Therefore, empirical studies on the effectiveness of such interventions in the AUD population are therefore scientifically proven and necessary in prevention and therapy of suicide risk. The aforementioned metacognitive therapy and cognitive-behavioural therapy focused on ruminations may be implemented into therapeutic programme for people with AUD representing a valuable form in reducing suicide risk. Certainly, these promising results require further exploration considering very limited research done in this field in AUD group. On the other hand, there is also research conducted on pharmacological interventions aimed at reducing the severity of ruminations. For example, combining amisulpride with antidepressant therapy in patients with treatment-resistant depression led to marked improvements in psychopathology in most patients, including those with severe ruminations (94). Aripiprazole monotherapy appears effective in reducing ruminations in cases of non-psychotic depression (95). Given that patients exhibiting increased ruminations are beneficiaries of treatment with antipsychotics (96), it is reasonable to focus future research on evaluating the efficacy of these therapies also in the AUD population who use this maladaptive emotion regulation strategy excessively.

## 5 Limitations and future research

Several limitations of this study are worth noting. First of all, the study was cross-sectional, which limits the ability to draw cause-and-effect conclusions. In addition, variables were measured as traits rather than as momentary mental states, meaning that they were analysed independently of situational context and temporal implications and thus limiting insight into the dynamics of the mutual links between the variables presented. This is important particularly in the AUD group, as their emotional state, the regulatory strategies used and potential suicidal behaviour can fluctuate considerably depending on a number of factors related to both clinical variables and situational context. Finally, testing a model of such complexity may introduce the risk of over-interpreting the results therefore the conclusions drawn from its validation are preliminary analyses requiring further scientific exploration.

Future research should use a longitudinal approach such as Ecological Momentary Assessment (EMA) to better understand the dynamic and time-varying relationships between ruminations, emotional reactivity and suicide risk. The use of EMA may also help to identify everyday emotional triggering moments (trigger moments) and explore how the activation, intensity and duration of negative emotions affect the relationship between ruminations and suicide risk, and in particular whether there are specific patterns of emotional reactivity that increase suicide risk in the AUD group (97). In line with research on variability and flexibility of emotion regulation, it is worth considering an analysis of whether different patterns of ruminative strategy use over time directly predict suicide risk in the AUD group (98).

## Data Availability

The raw data supporting the conclusions of this article will be made available by the authors, without undue reservation.
