# Supplementary material for: A moderated mediation model in assessing links between rumination, emotional reactivity, and suicidal risk in alcohol use disorder
Source: Front Psychiatry. 2025 Feb 28;16:1479827. doi: 10.3389/fpsyt.2025.1479827 (PMC11907195; doi:10.3389/fpsyt.2025.1479827)

Supplementary Material A

Comparison of Linear and Quadratic Relationships Based on R-Squared Coefficients and Plots

1 Healthy Control Group

1.1 Linear and Quadratic Relationships with the SBQR Score

Figure 1: Relationship between PERS General Emotional Reactivity and SBQR Score

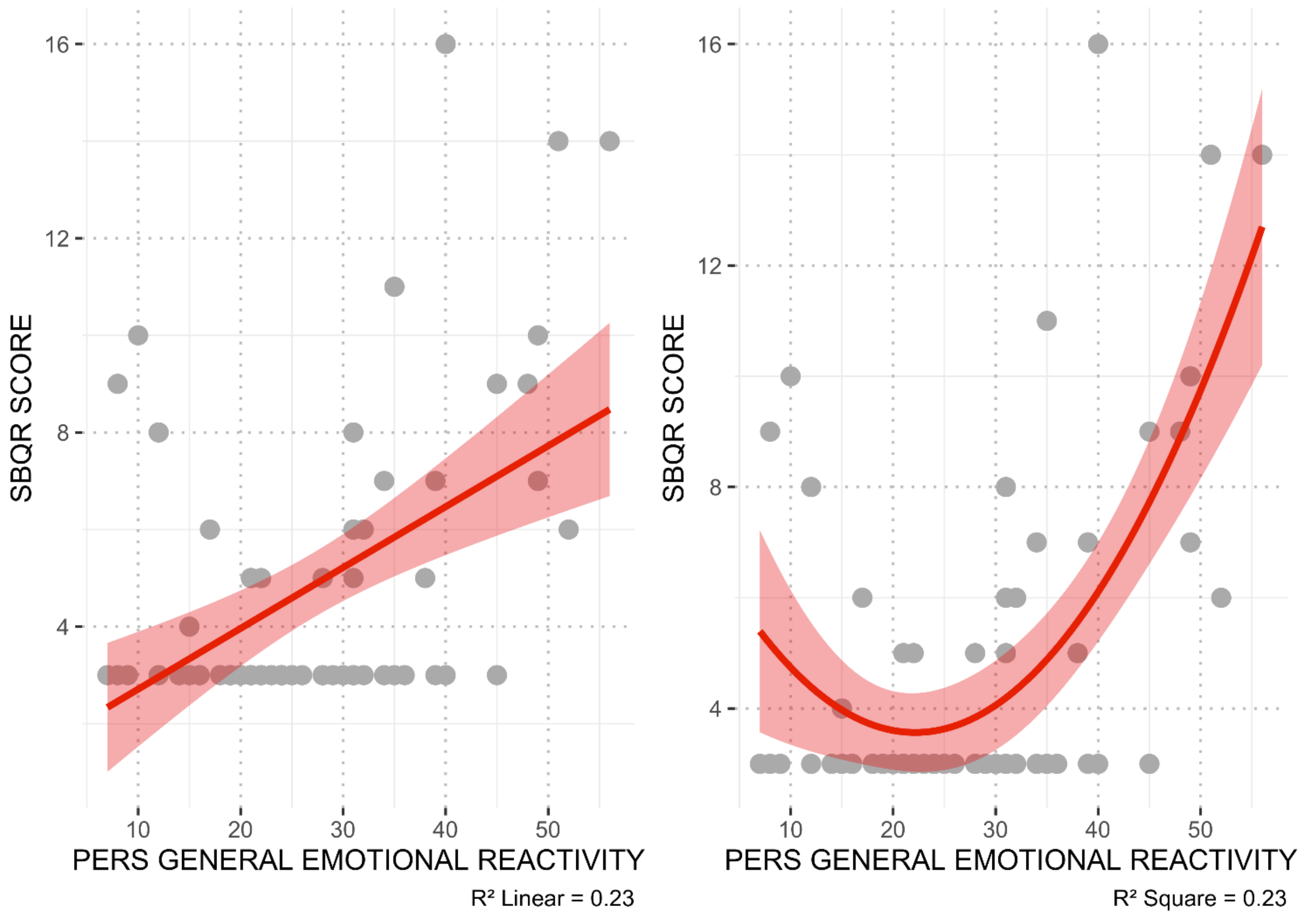

Figure 2: Relationship between HADS Depression and SBQR Score

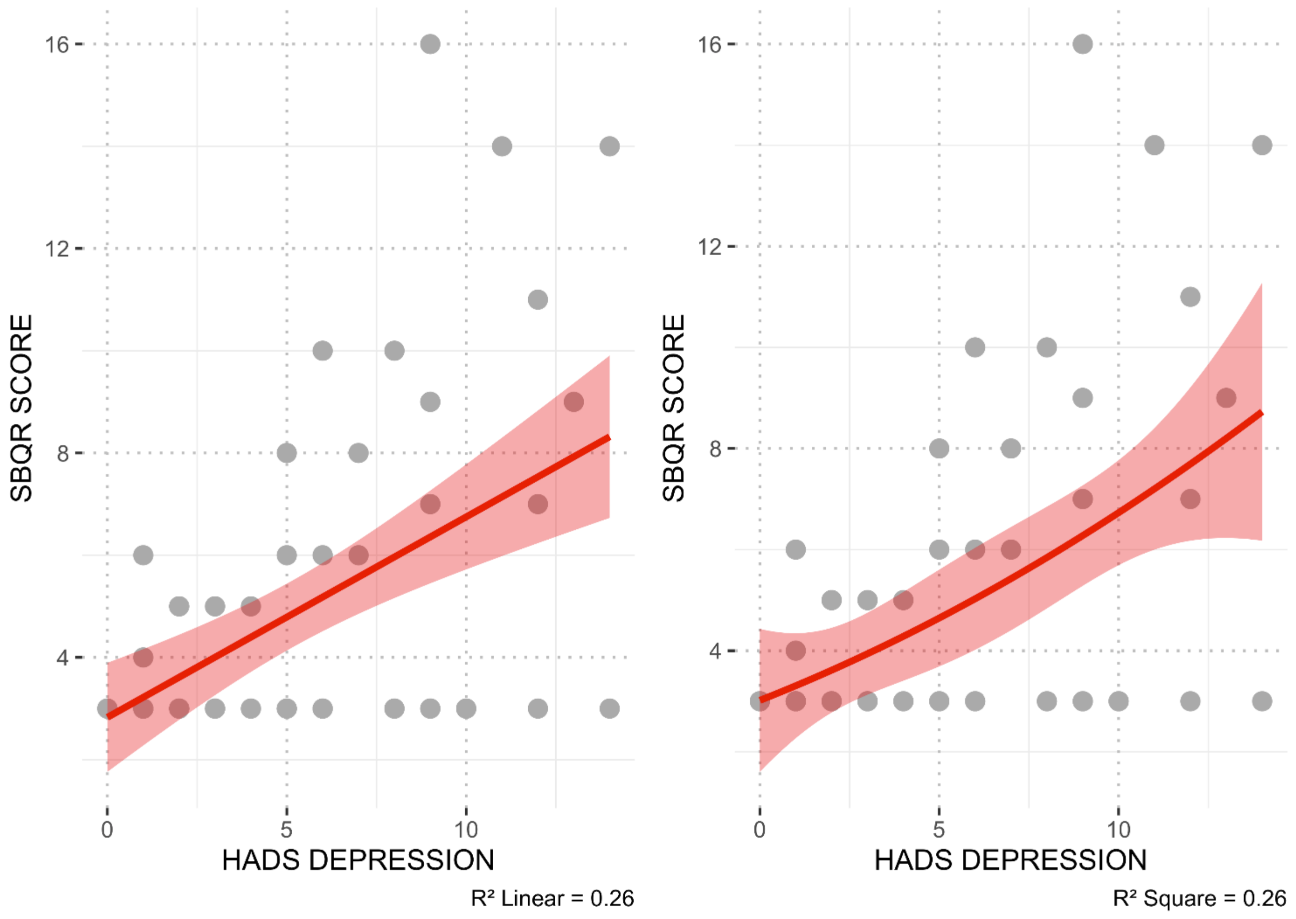

Figure 3 Relationship between Age and SBQR Score

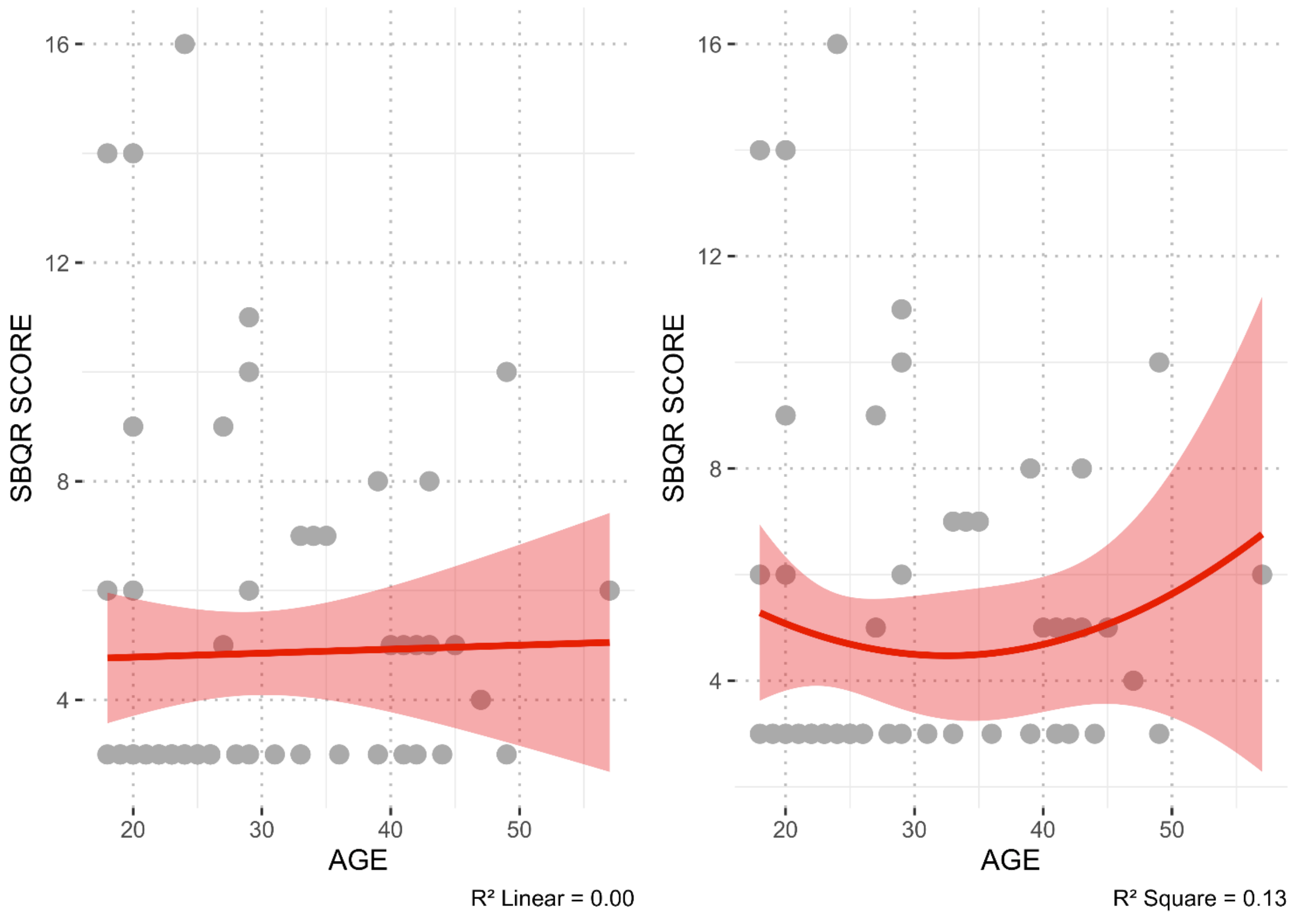

Figure 4: Relationship between Sex and SBQR Score

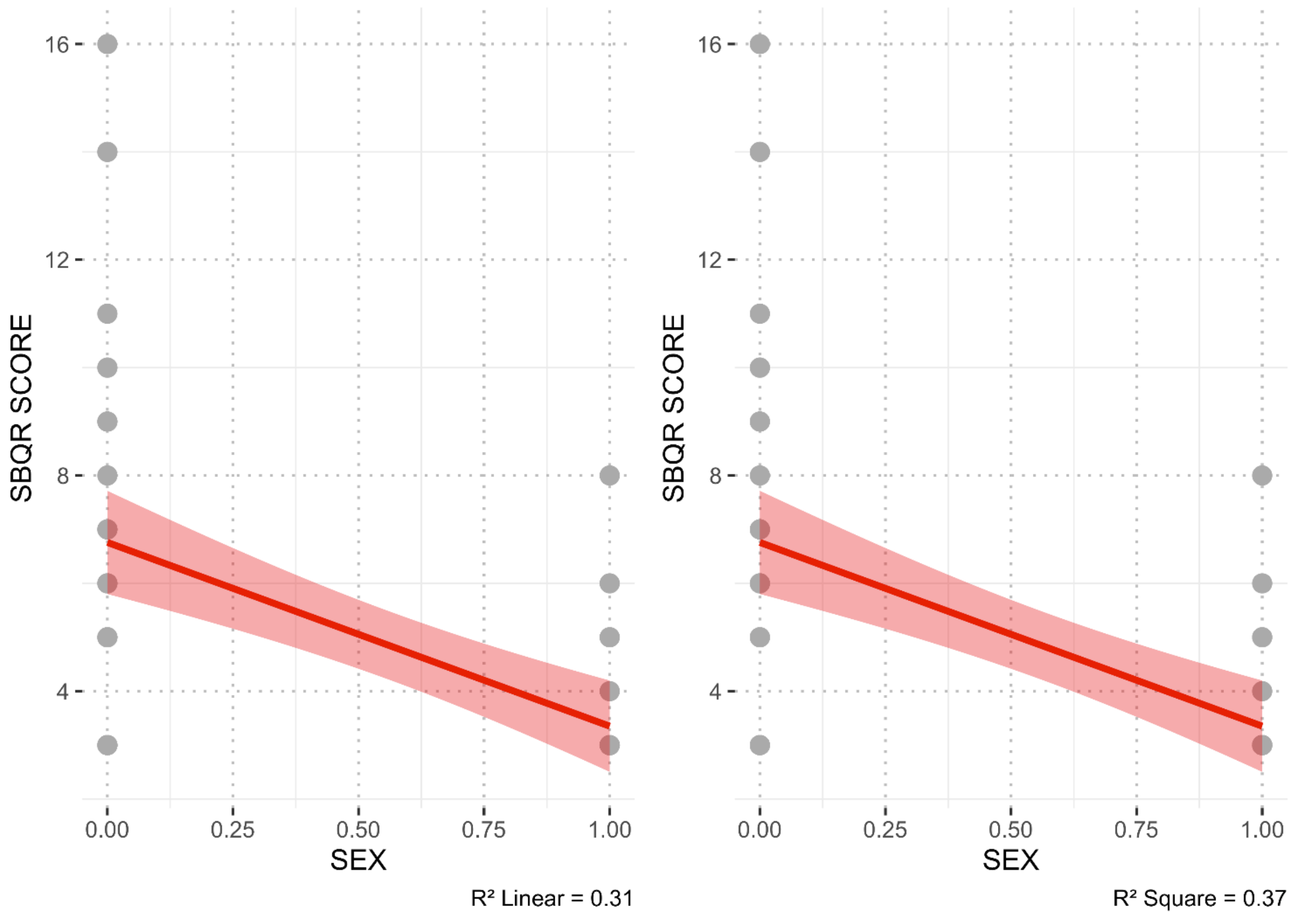

Figure 5: Relationship between Education and SBQR Score

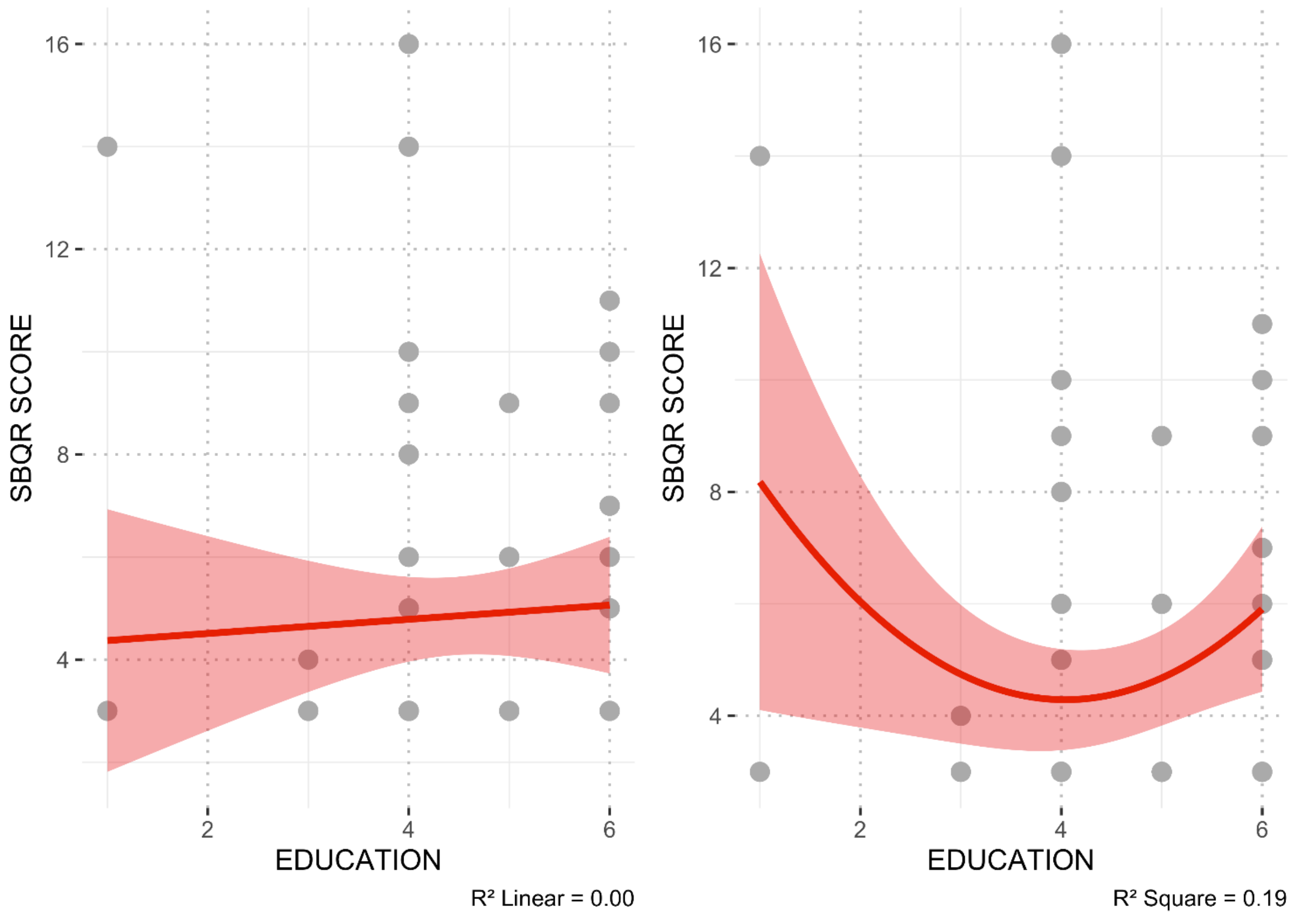

Figure 6: Relationship between CERQ Rumination and SBQR Score

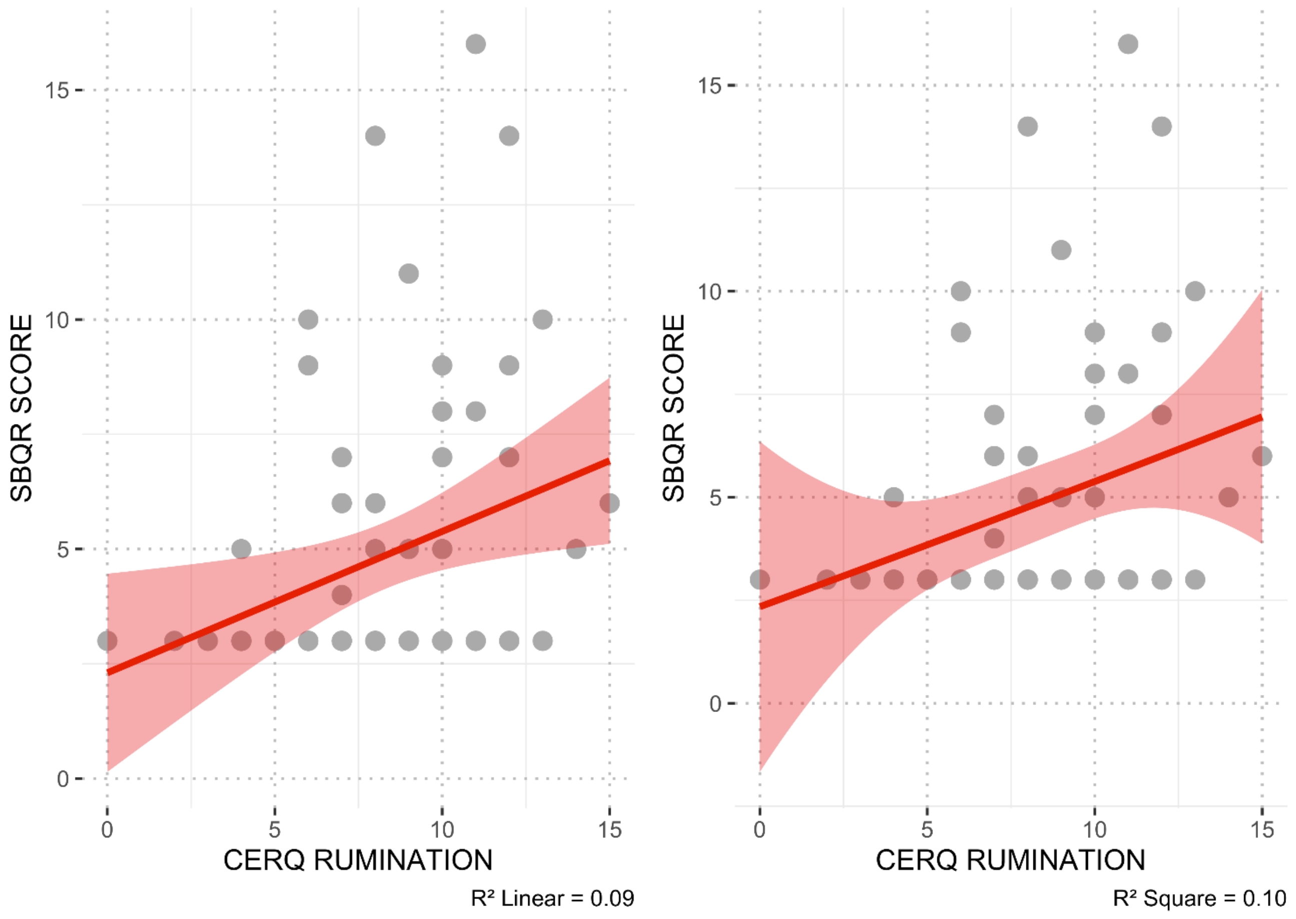

Figure 7: Relationship between AUDIT Score and SBQR Score

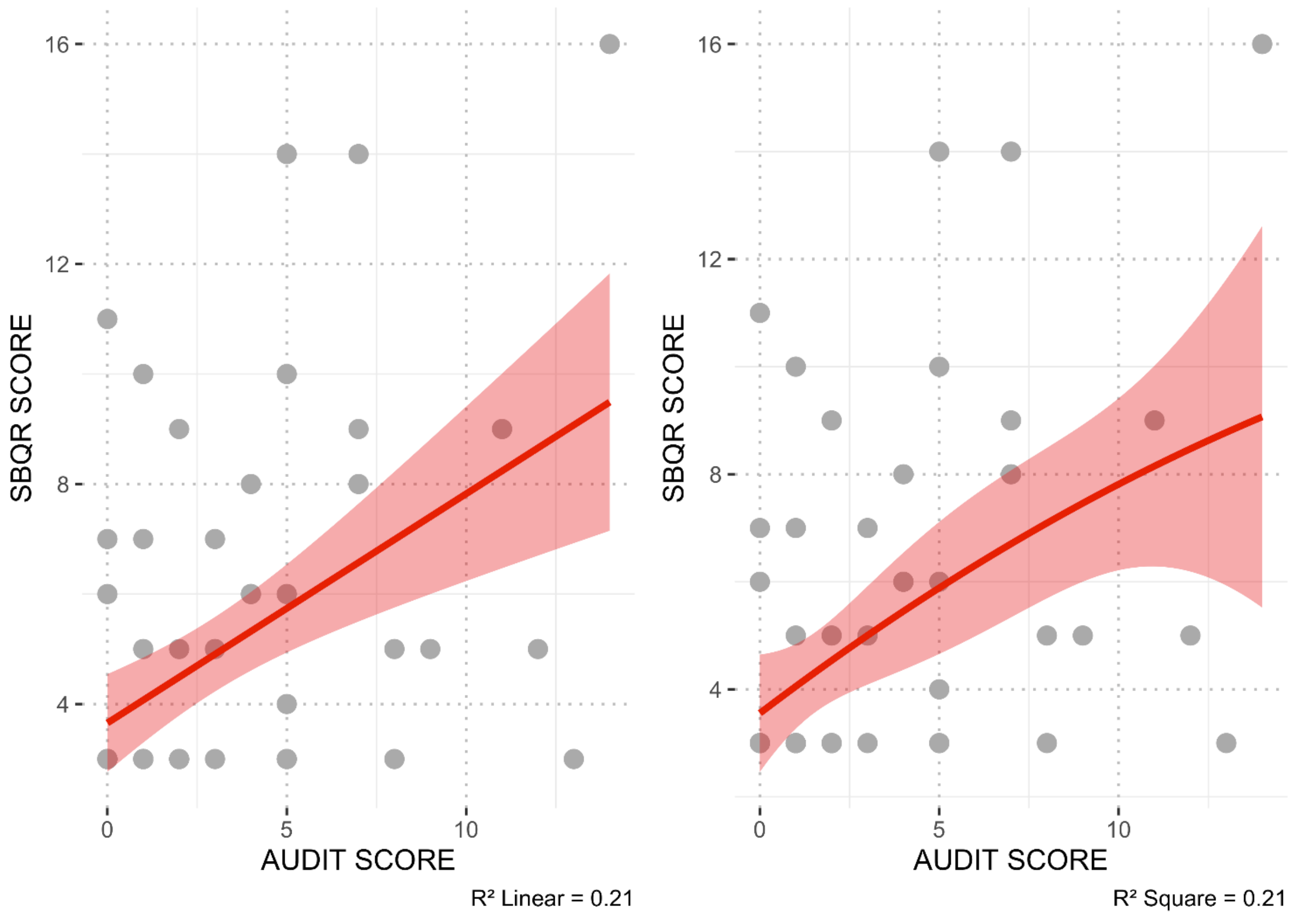

Linear and Quadratic Relationships with PERS General Emotional Reactivity

Figure 8: Relationship between HADS Depression and PERS General Emotional Reactivity

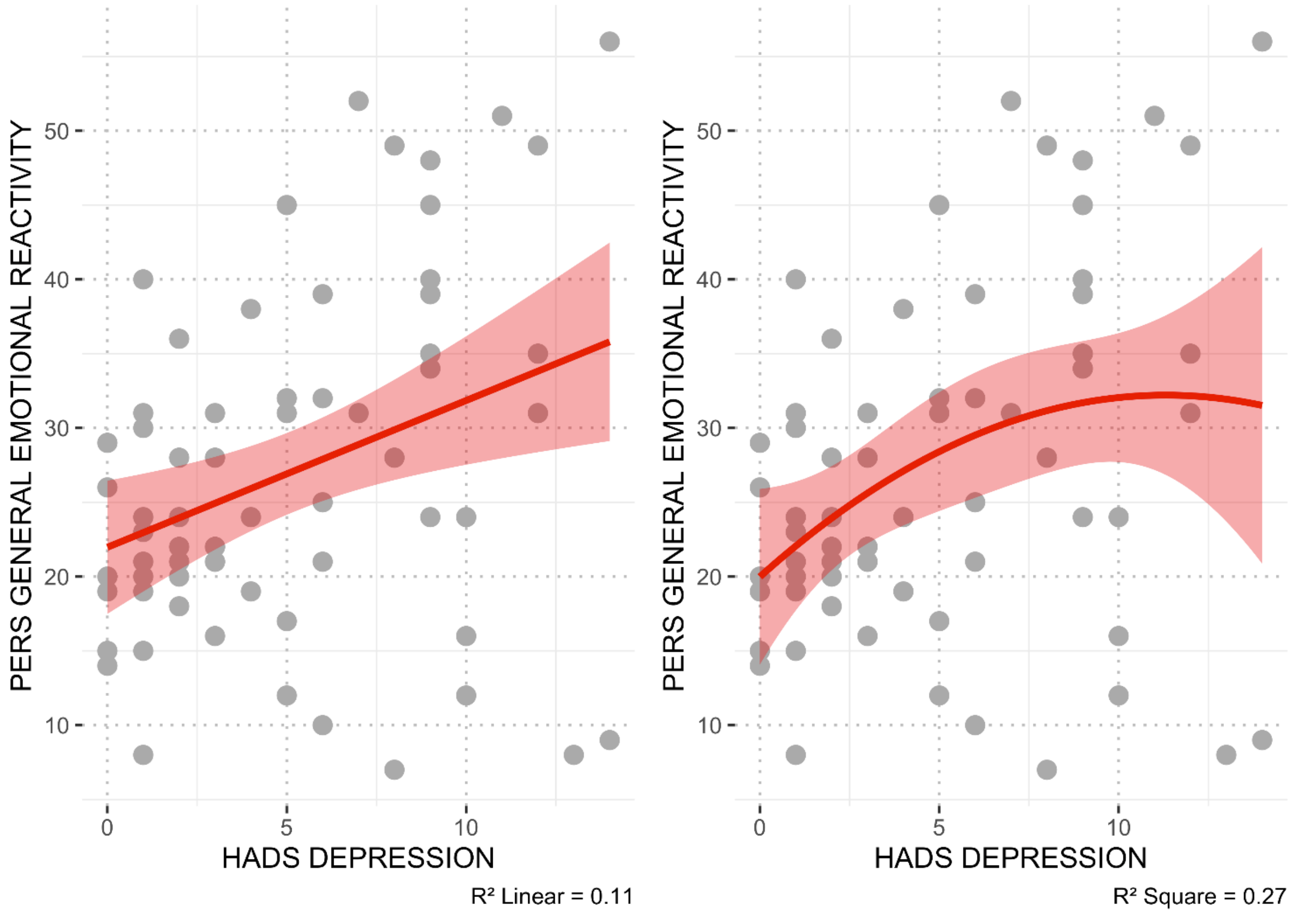

Figure 9: Relationship between Age and PERS General Emotional Reactivity

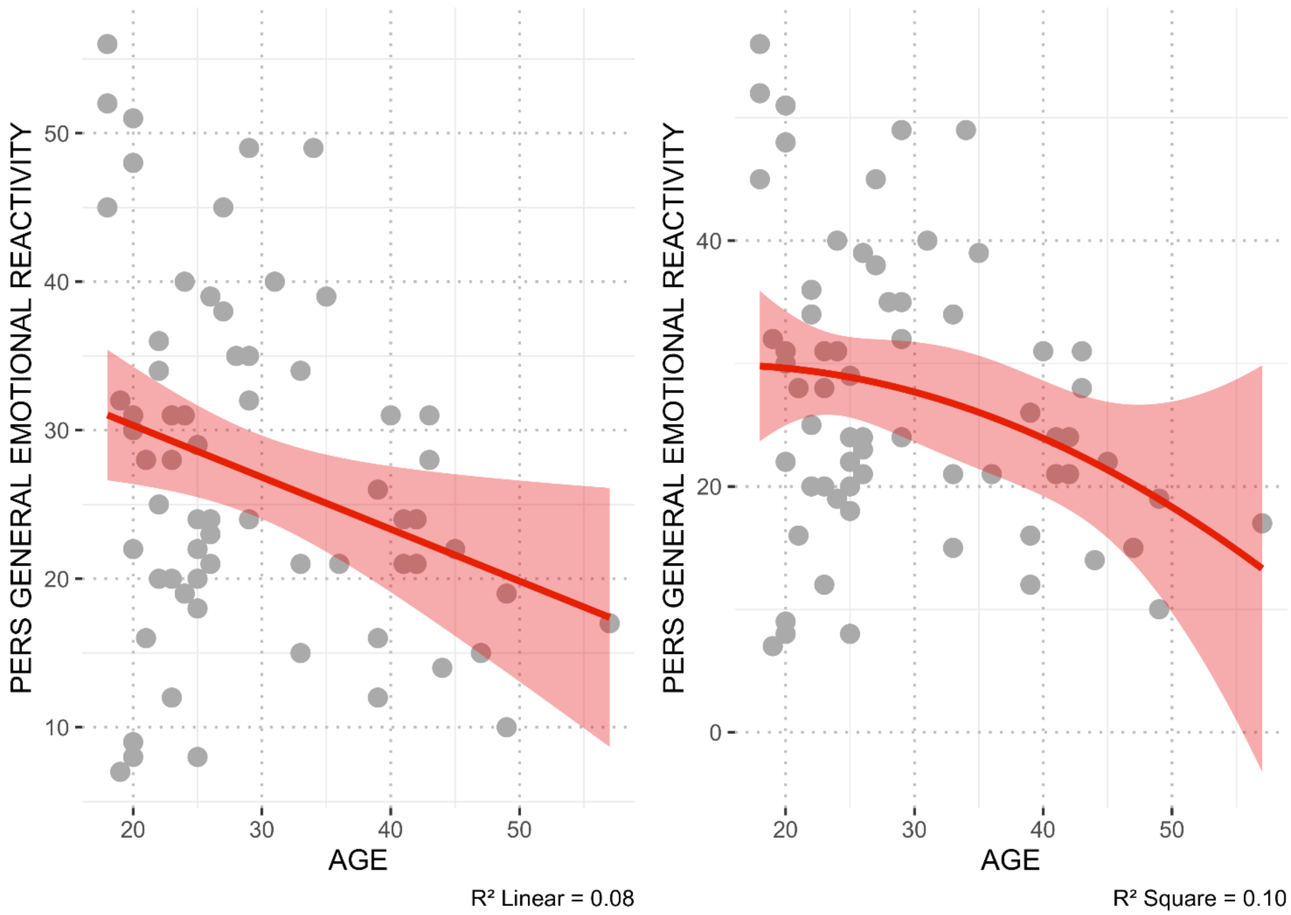

Figure 10: Relationship between Sex and PERS General Emotional Reactivity

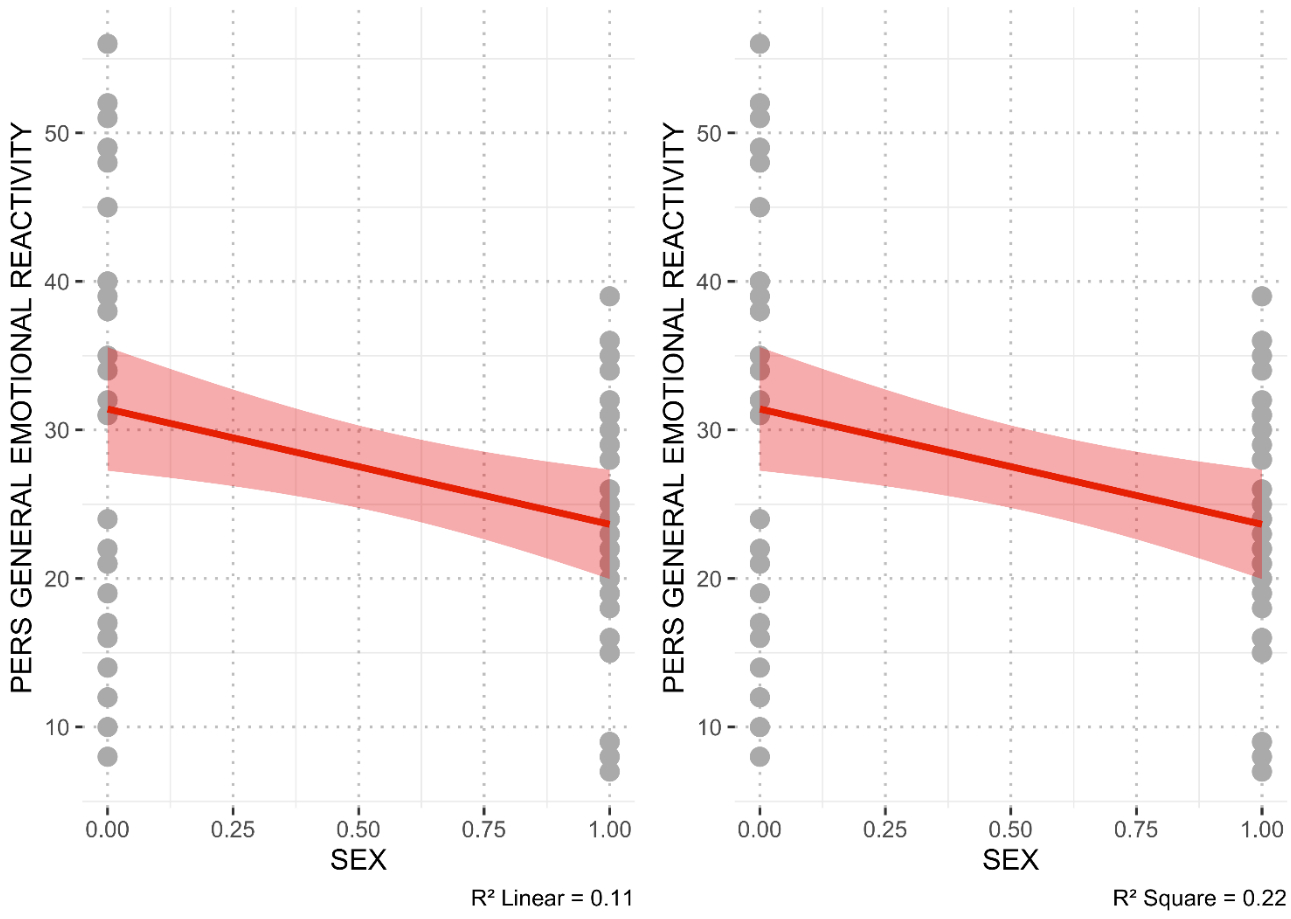

Figure 11

Relationship between Education and PERS General Emotional Reactivity

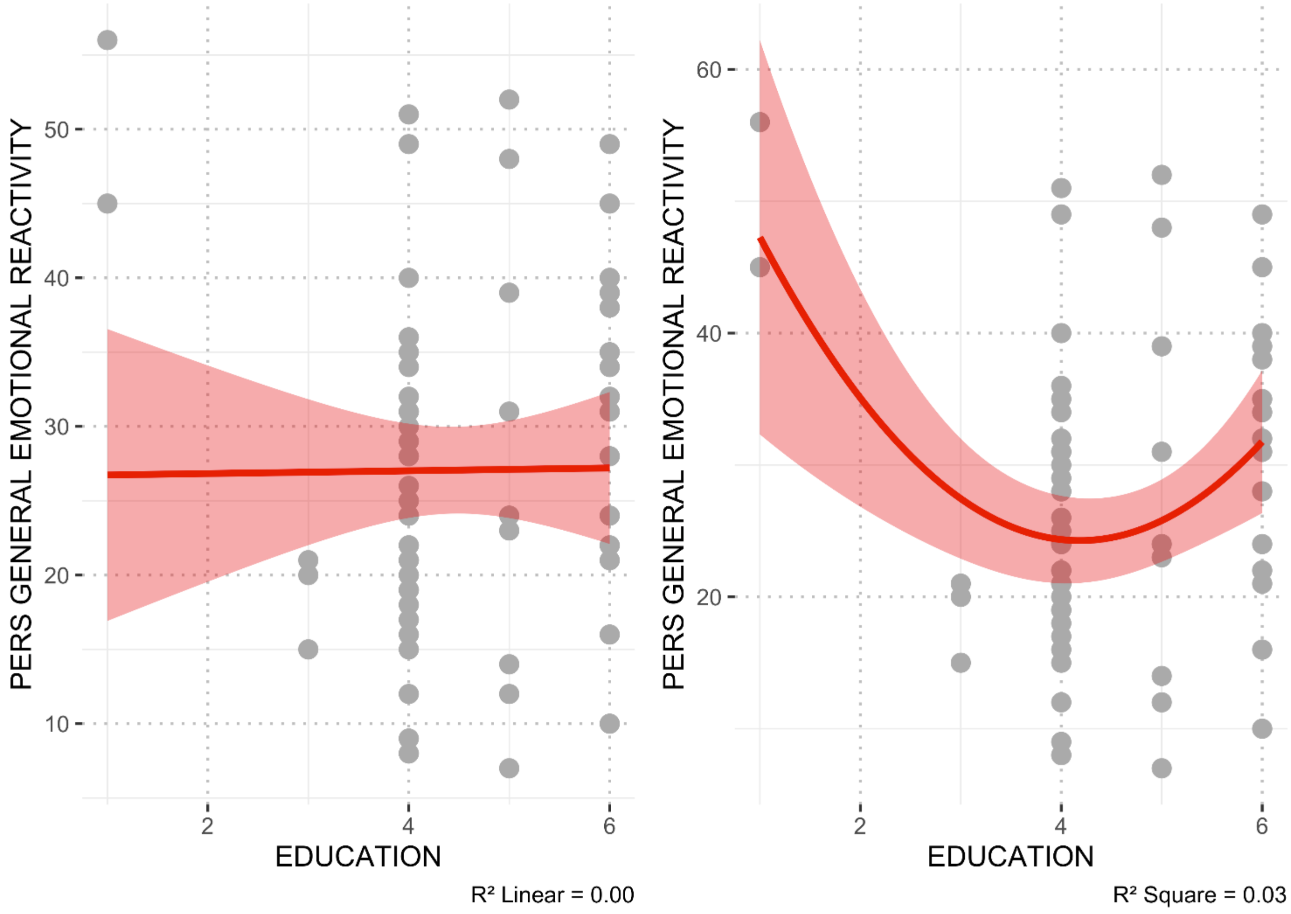

Figure 12

Relationship between CERQ Rumination and PERS General Emotional Reactivity

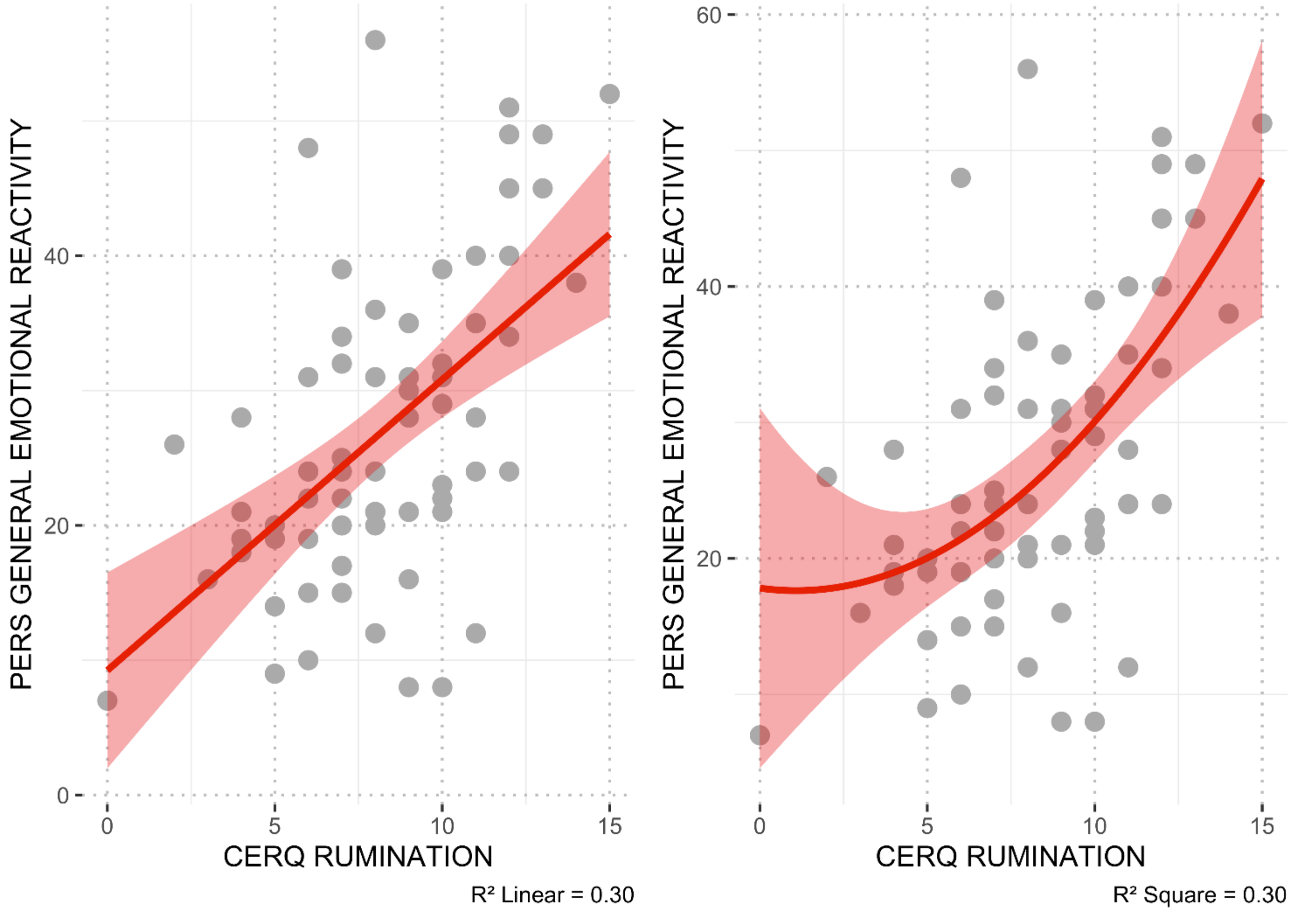

Figure 13

Relationship between AUDIT Score and PERS General Emotional Reactivity

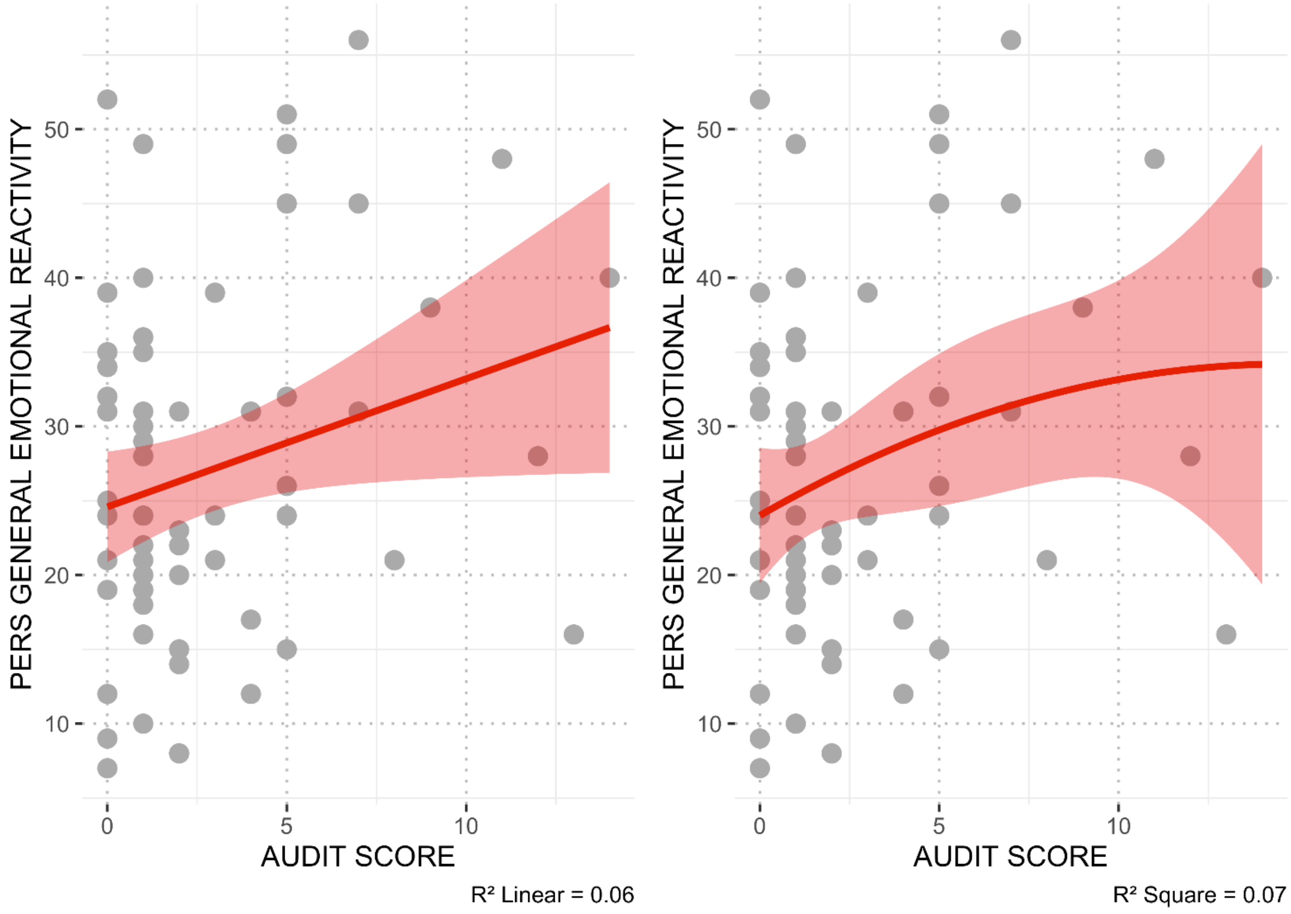

2 Alcohol Use Disorder Group

2.1 Linear and Quadratic Relationships with the SBQR Score

Figure 1

Relationship between PERS General Emotional Reactivity and SBQR Score

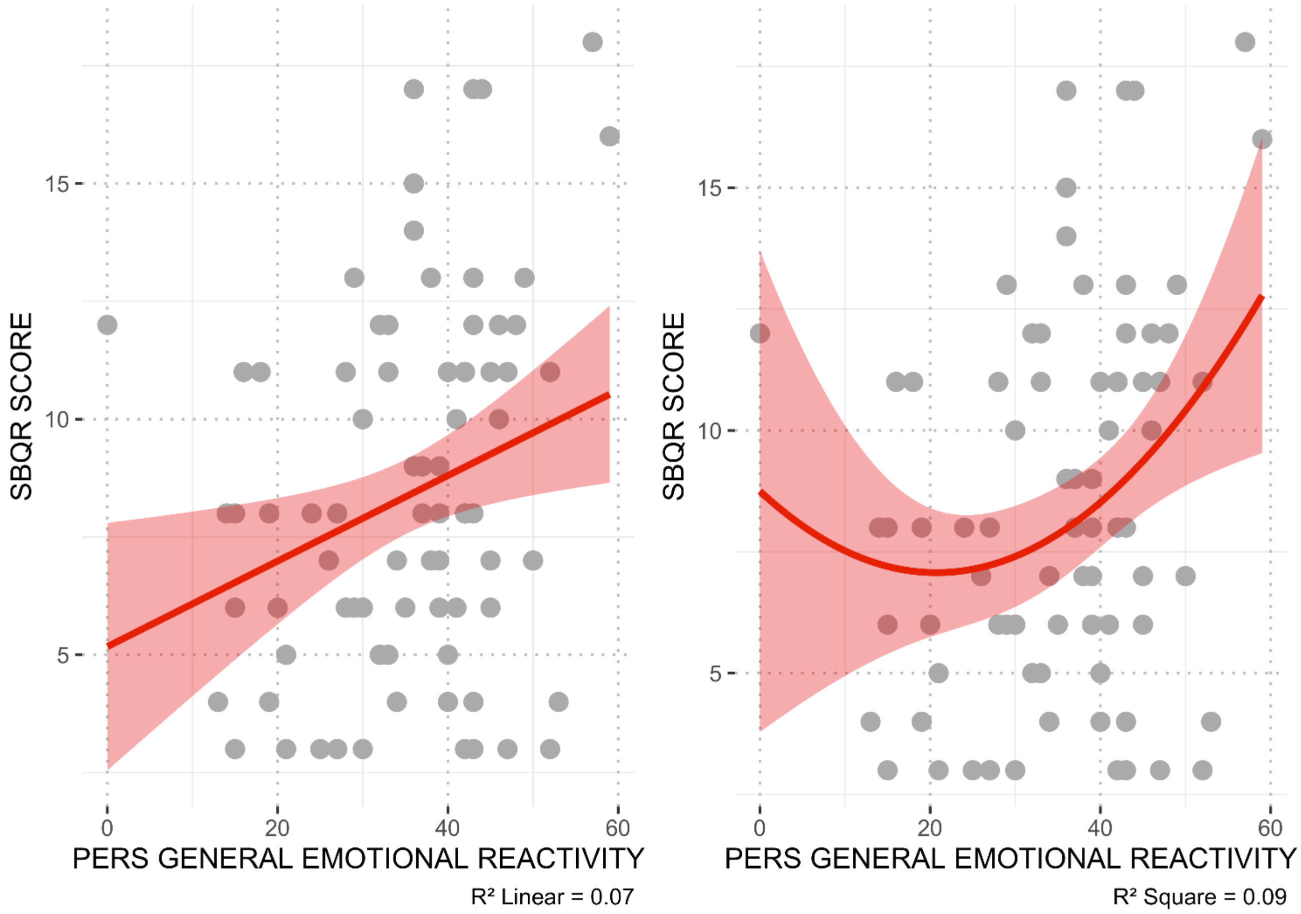

Figure 2

Relationship between HADS Depression and SBQR Score

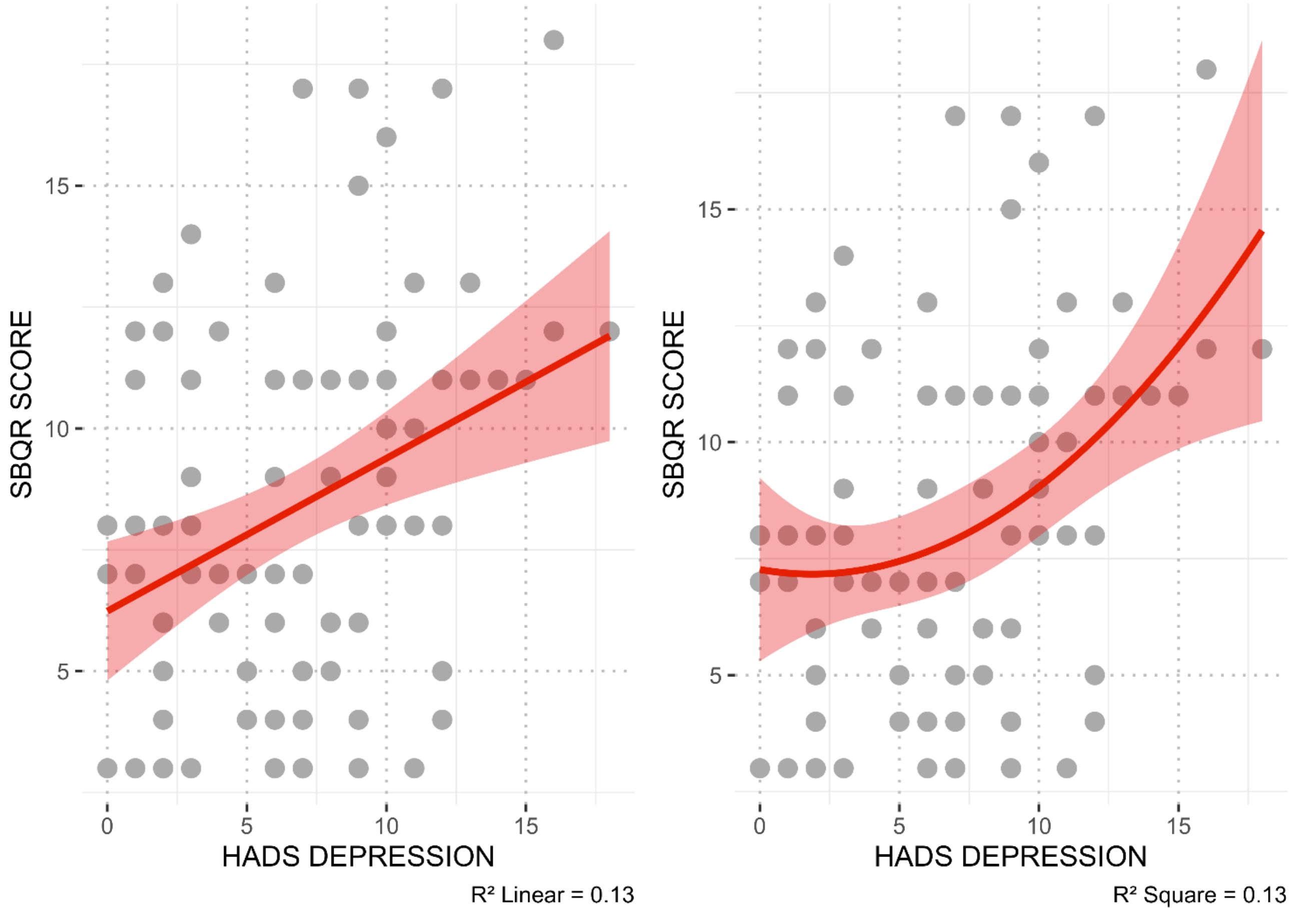

Figure 3

Relationship between Age and SBQR Score

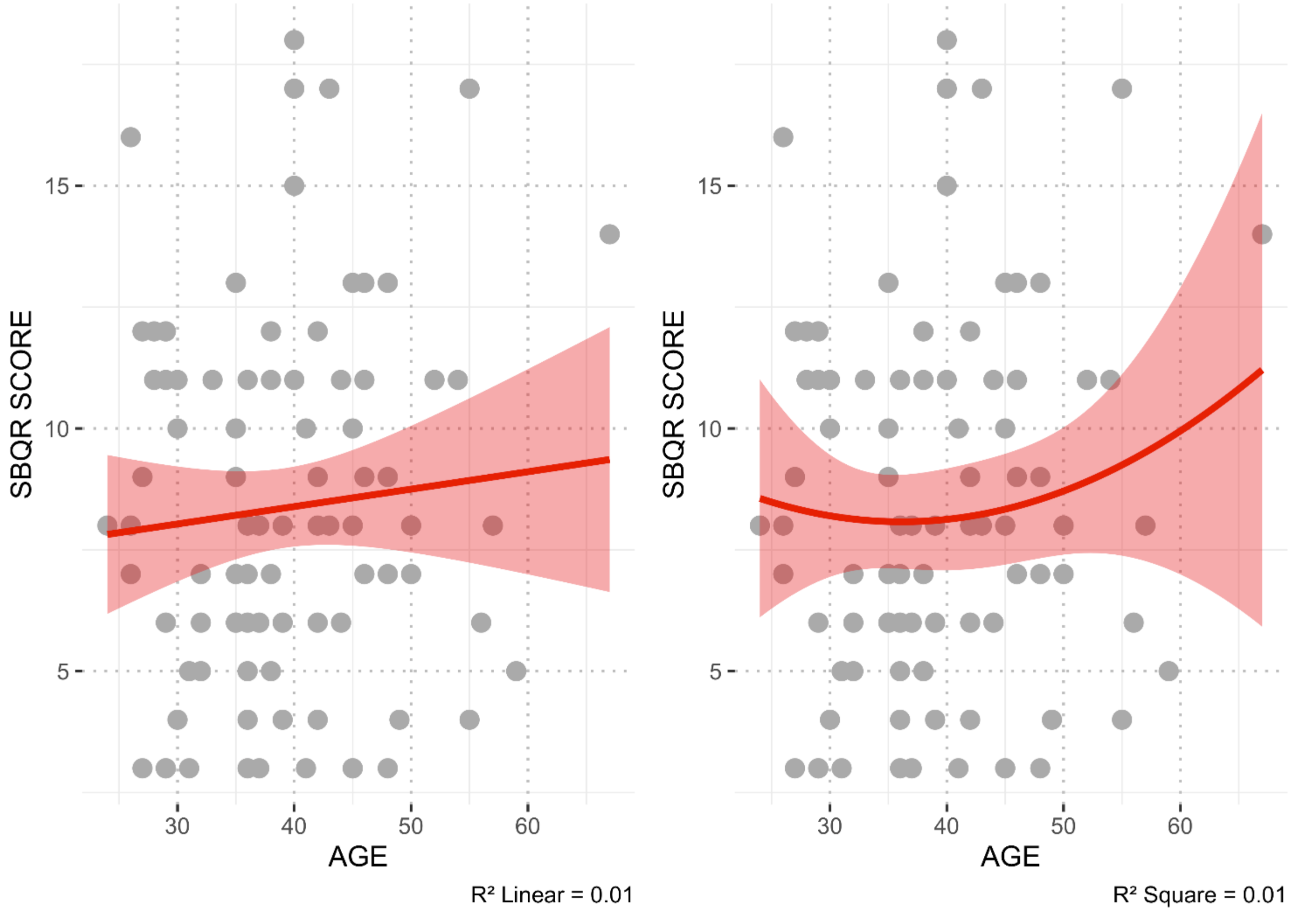

Figure 4

Relationship between Sex and SBQR Score

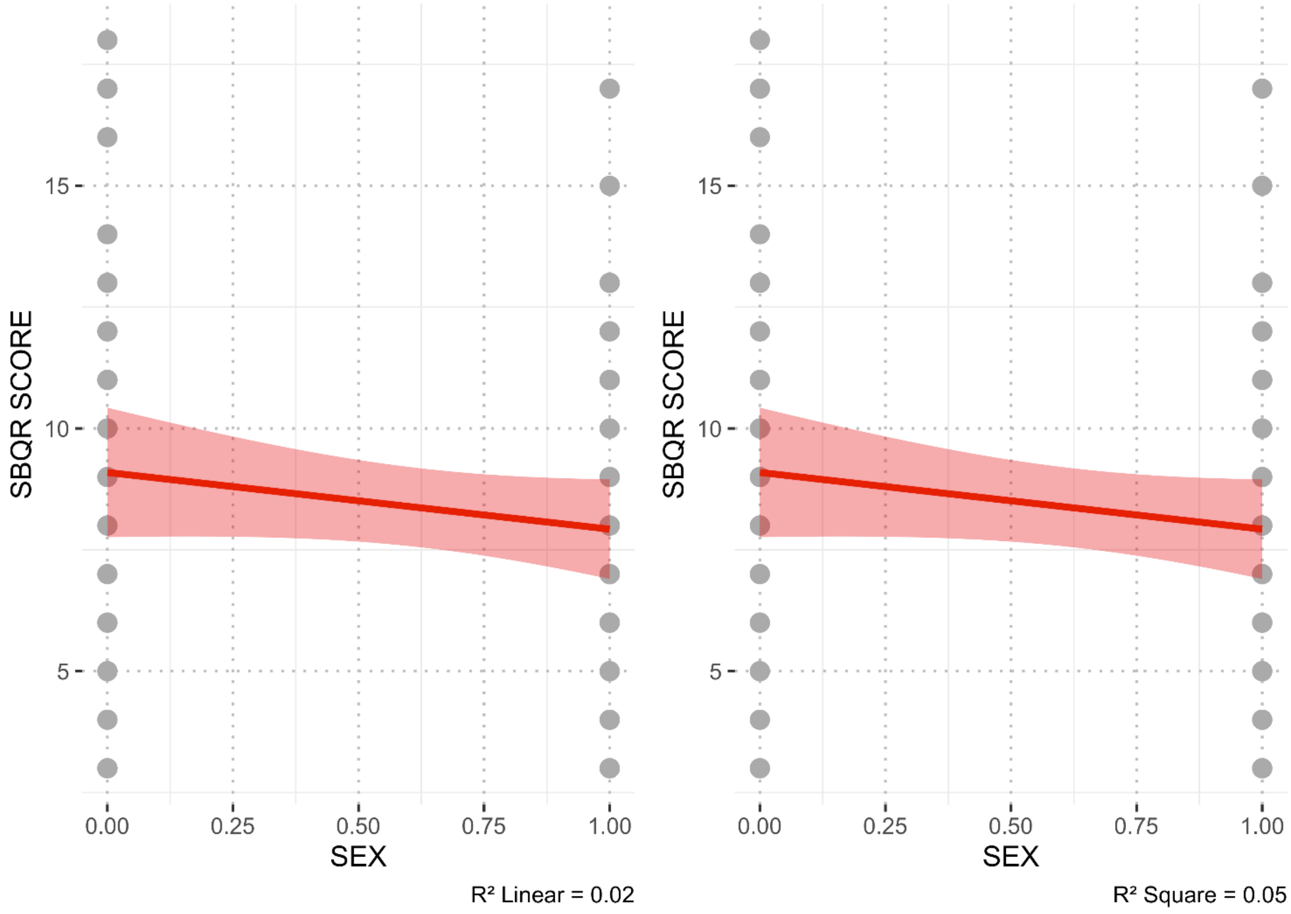

Figure 5

Relationship between Education and SBQR Score

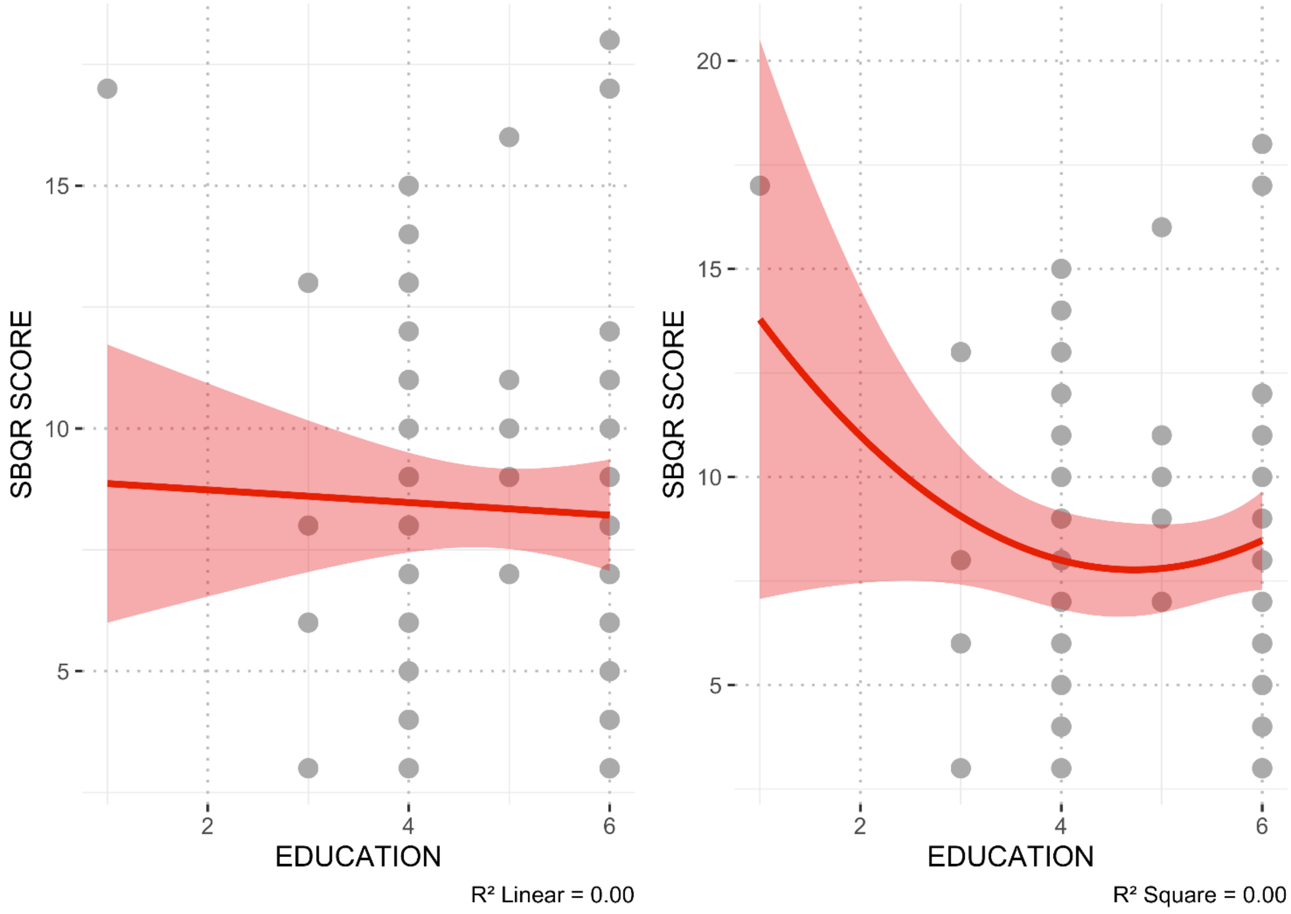

Figure 6

Relationship between CERQ Rumination and SBQR Score

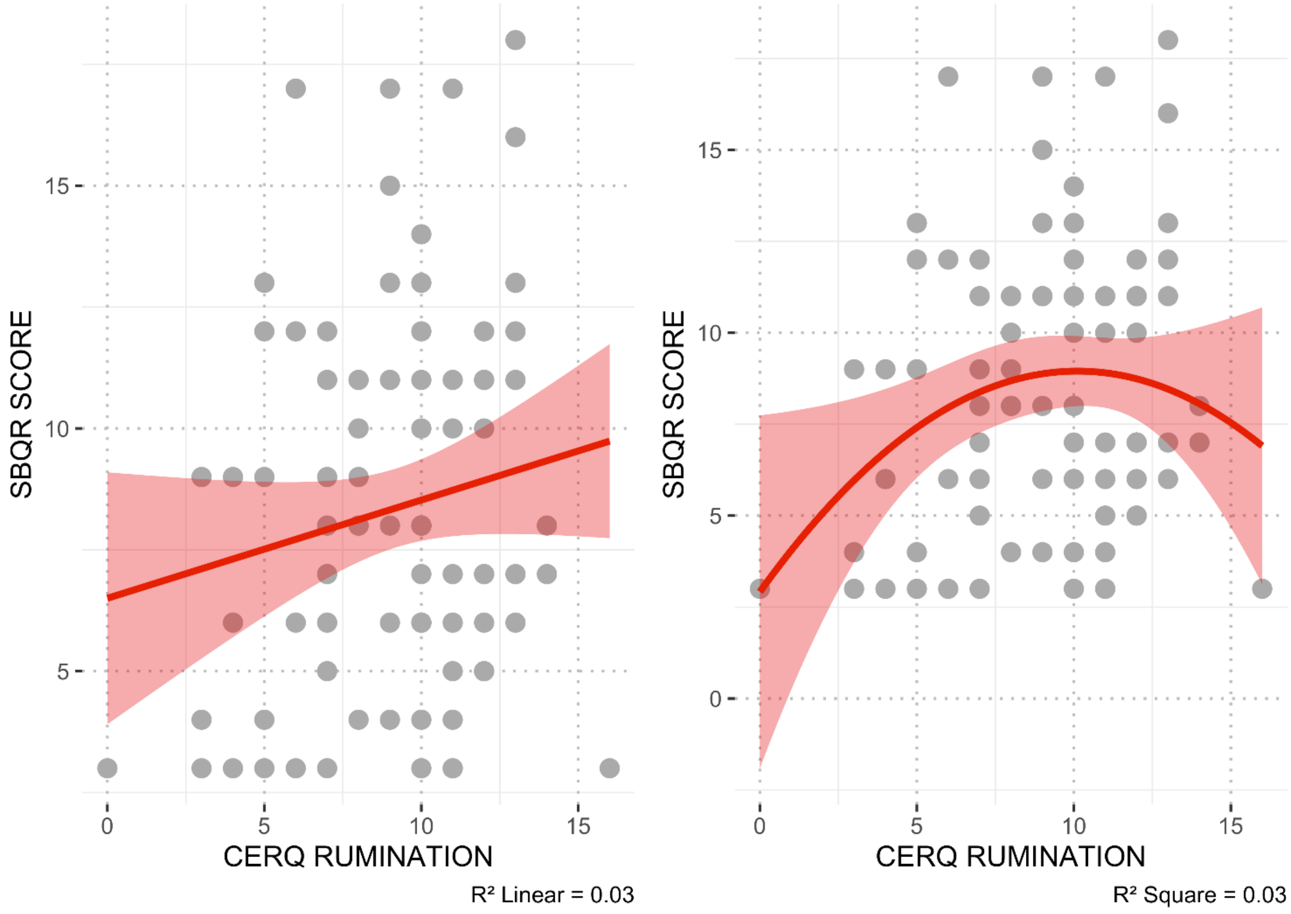

Figure 7

Relationship between AUDIT Score and SBQR Score

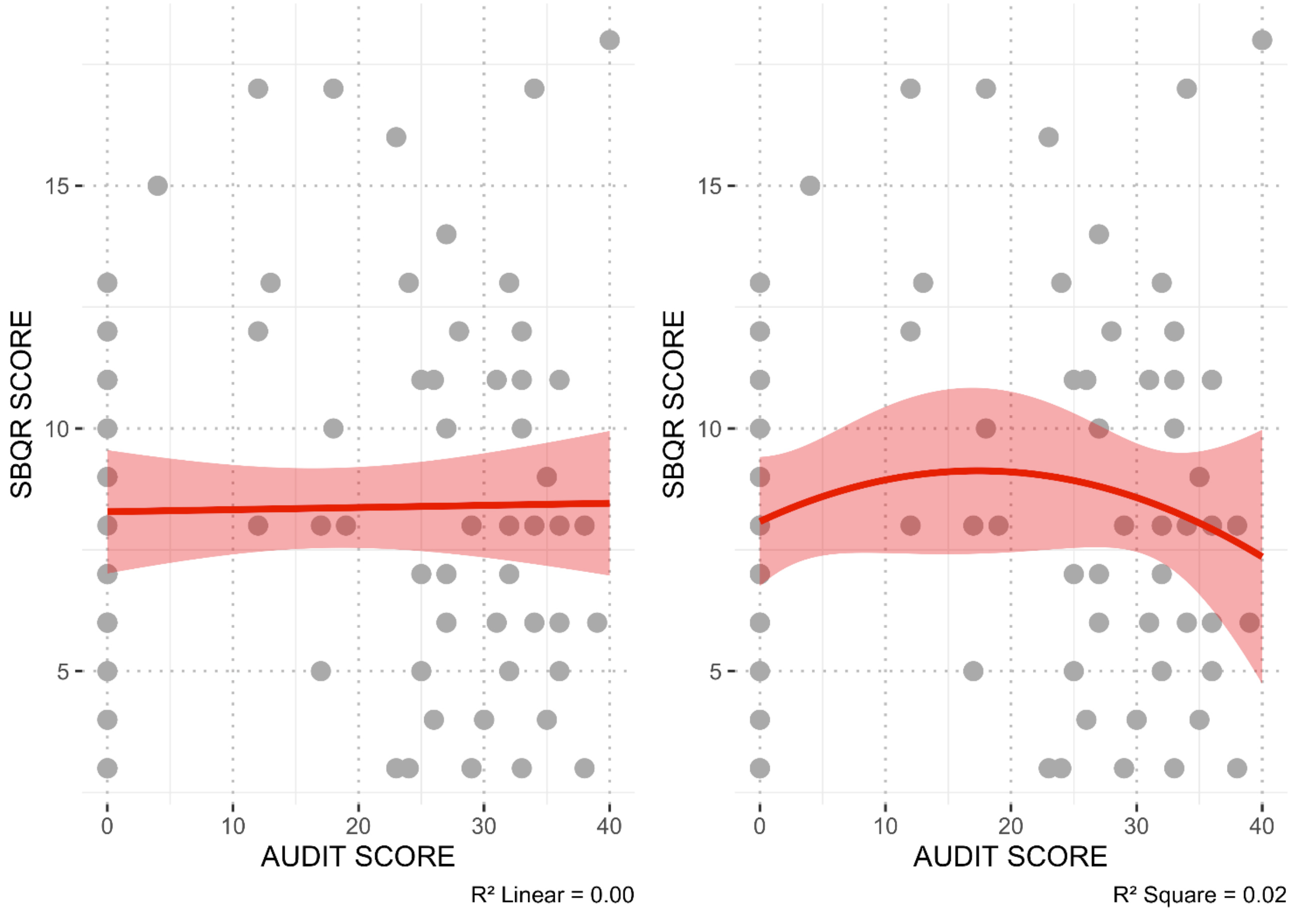

Linear and Quadratic Relationships with PERS General Emotional Reactivity

Figure 8

Relationship between HADS Depression and PERS General Emotional Reactivity

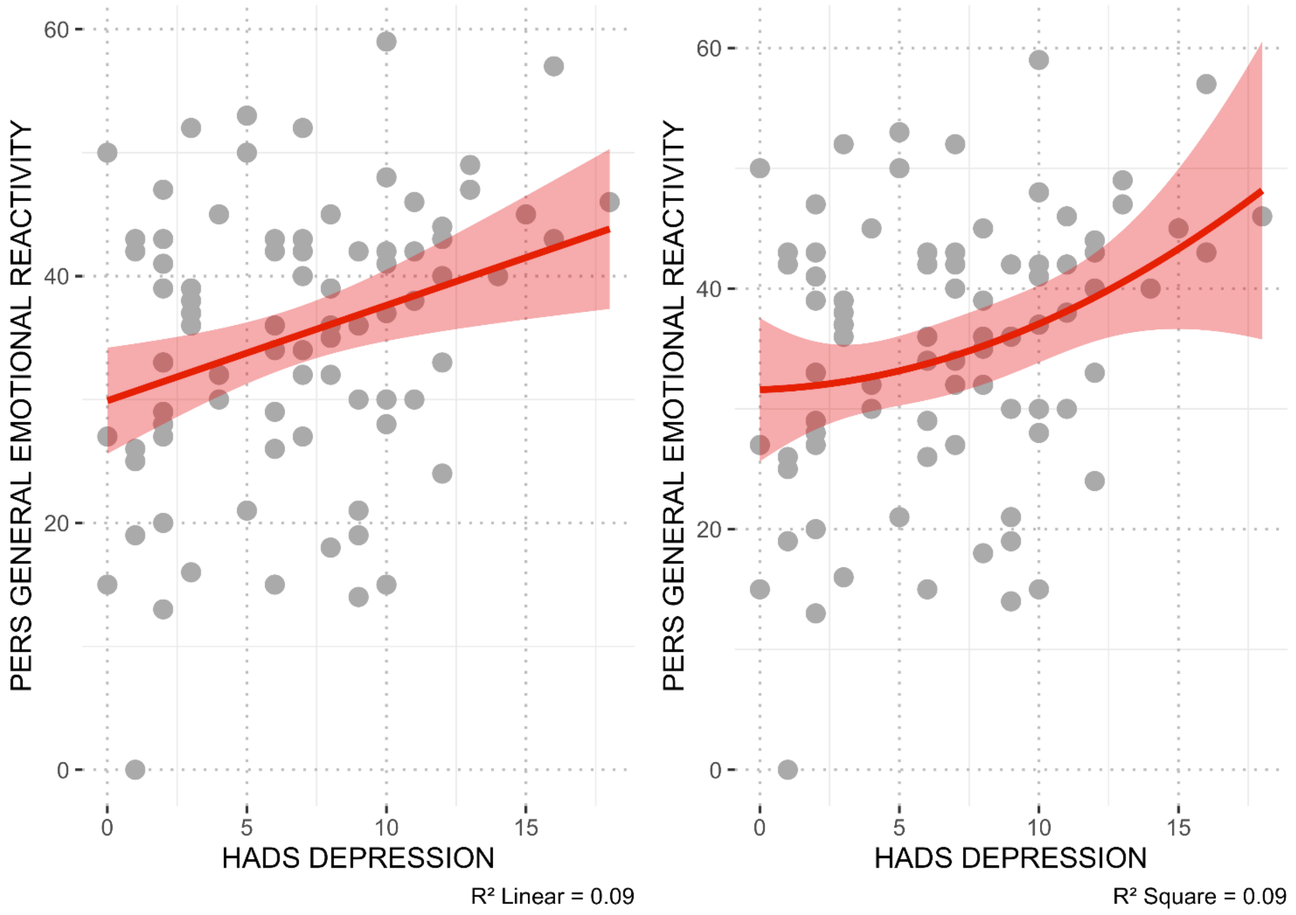

Figure 9

Relationship between Age and PERS General Emotional Reactivity

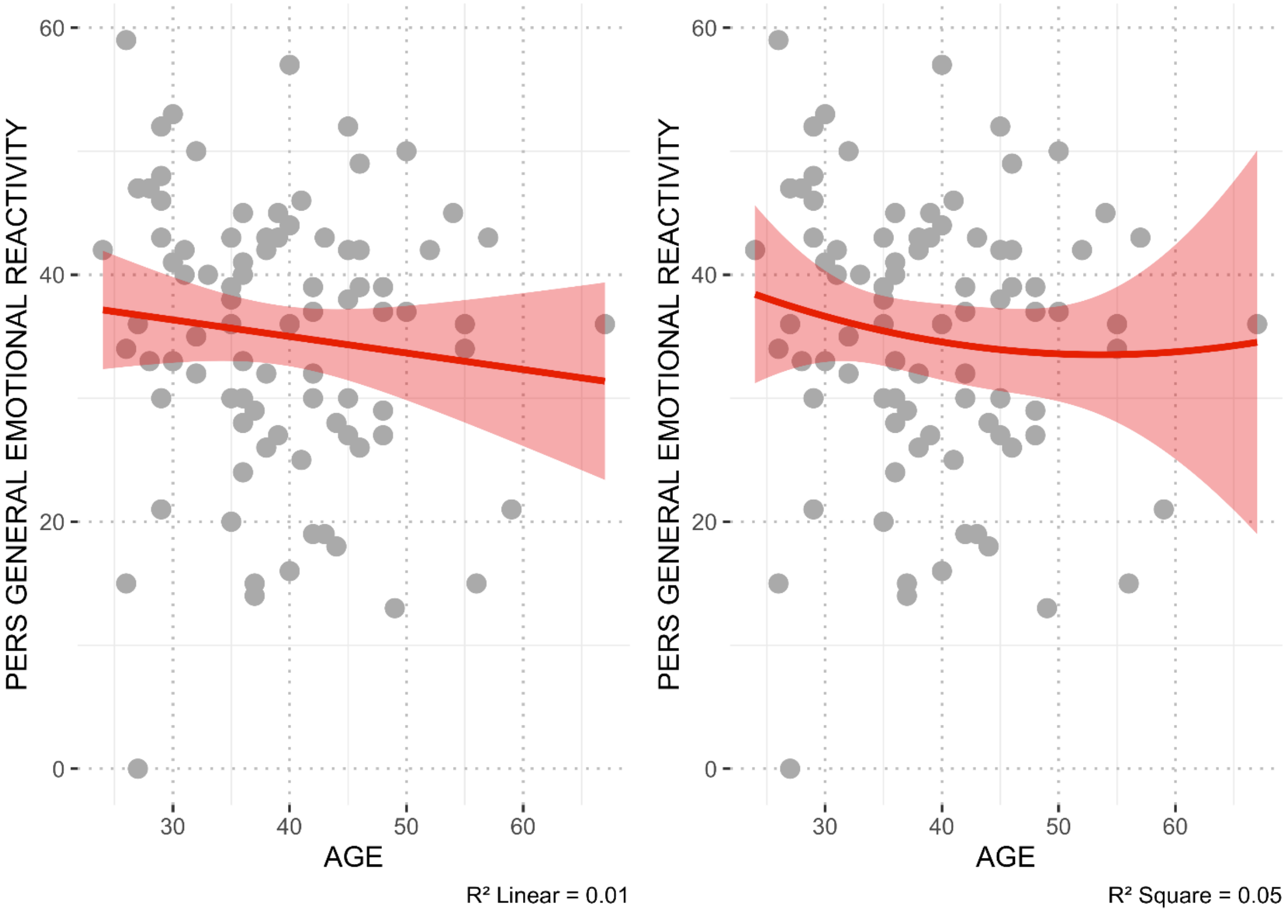

Figure 10

Relationship between Sex and PERS General Emotional Reactivity

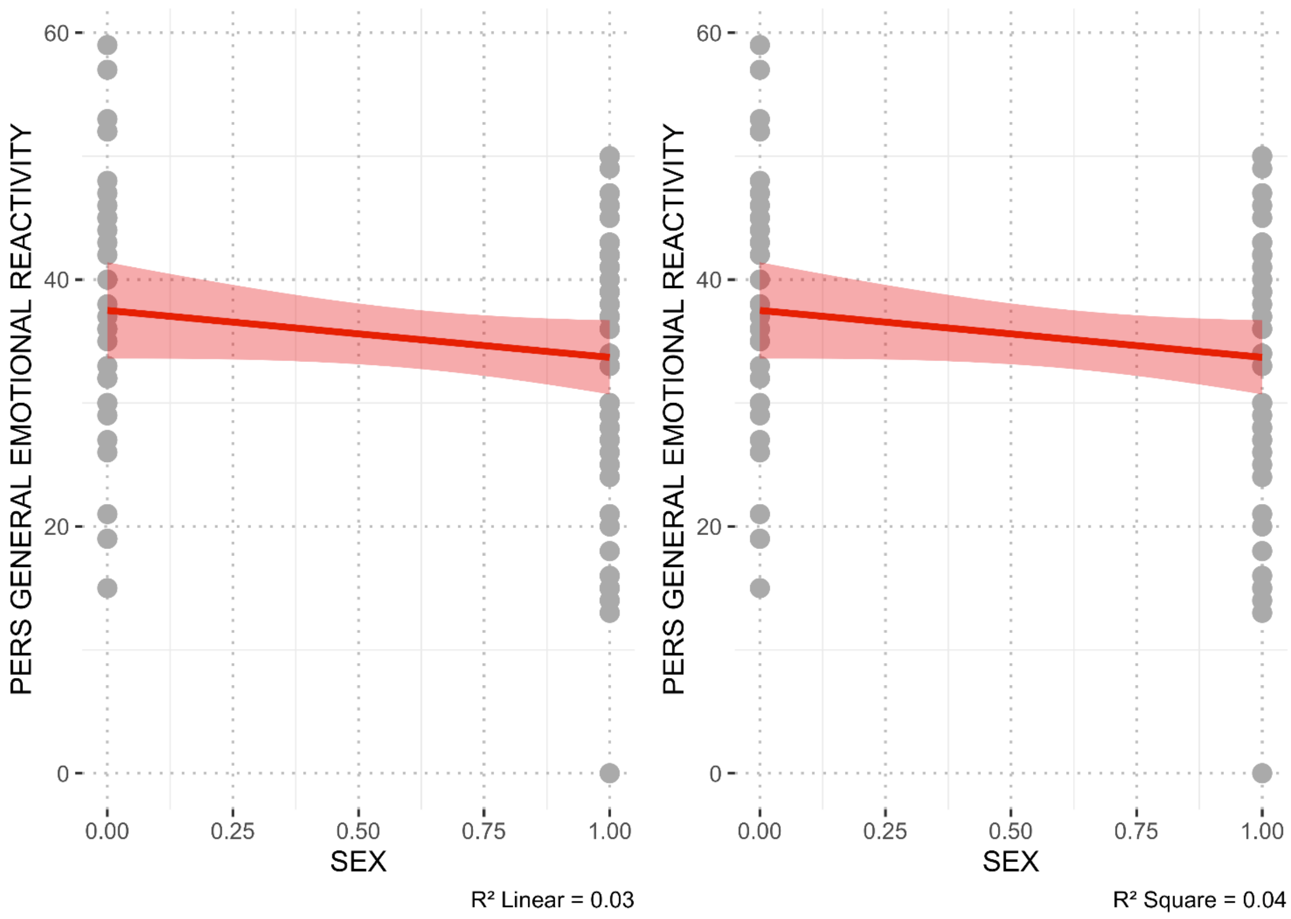

Figure 11

Relationship between Education and PERS General Emotional Reactivity

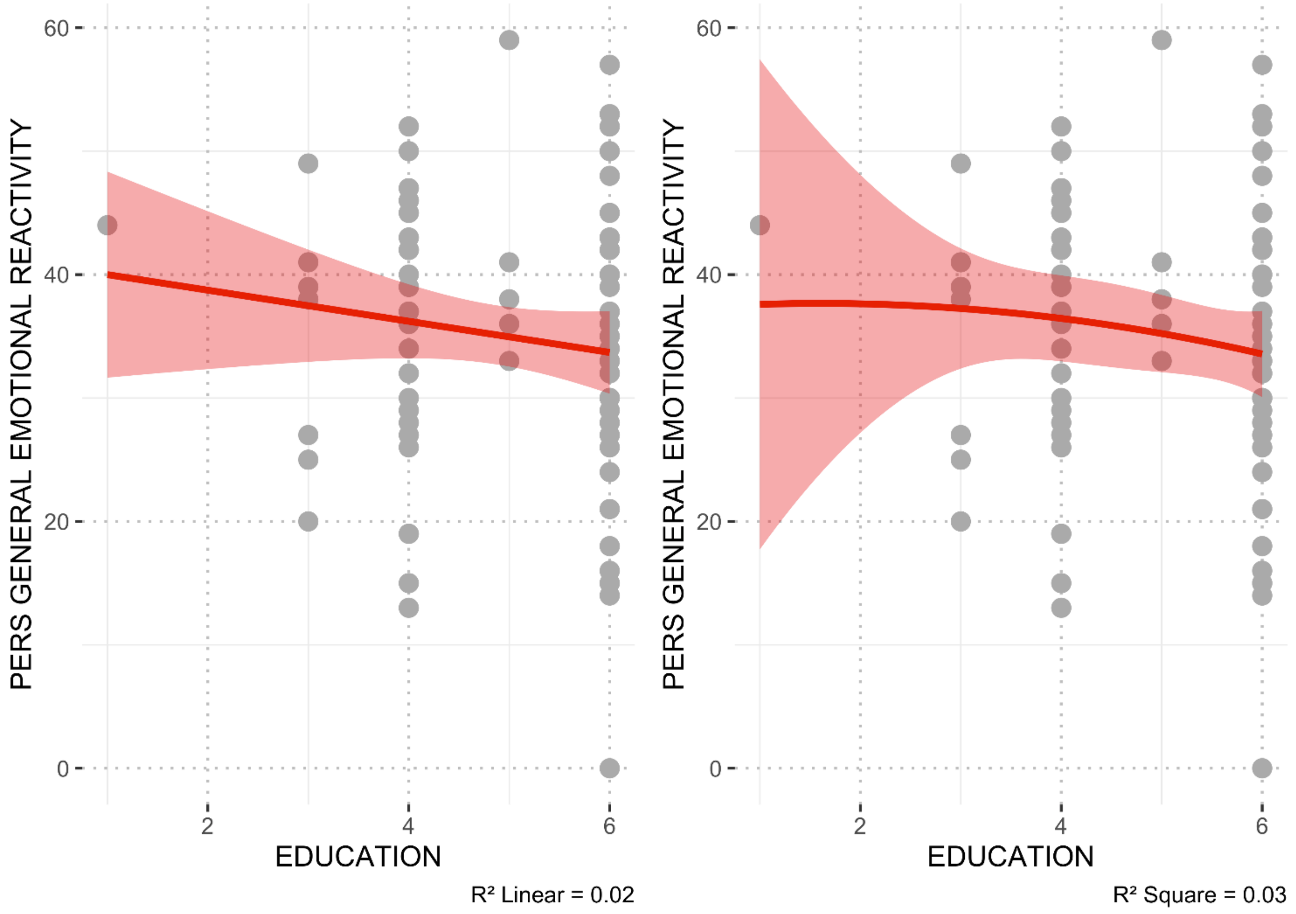

Figure 12

Relationship between CERQ Rumination and PERS General Emotional Reactivity

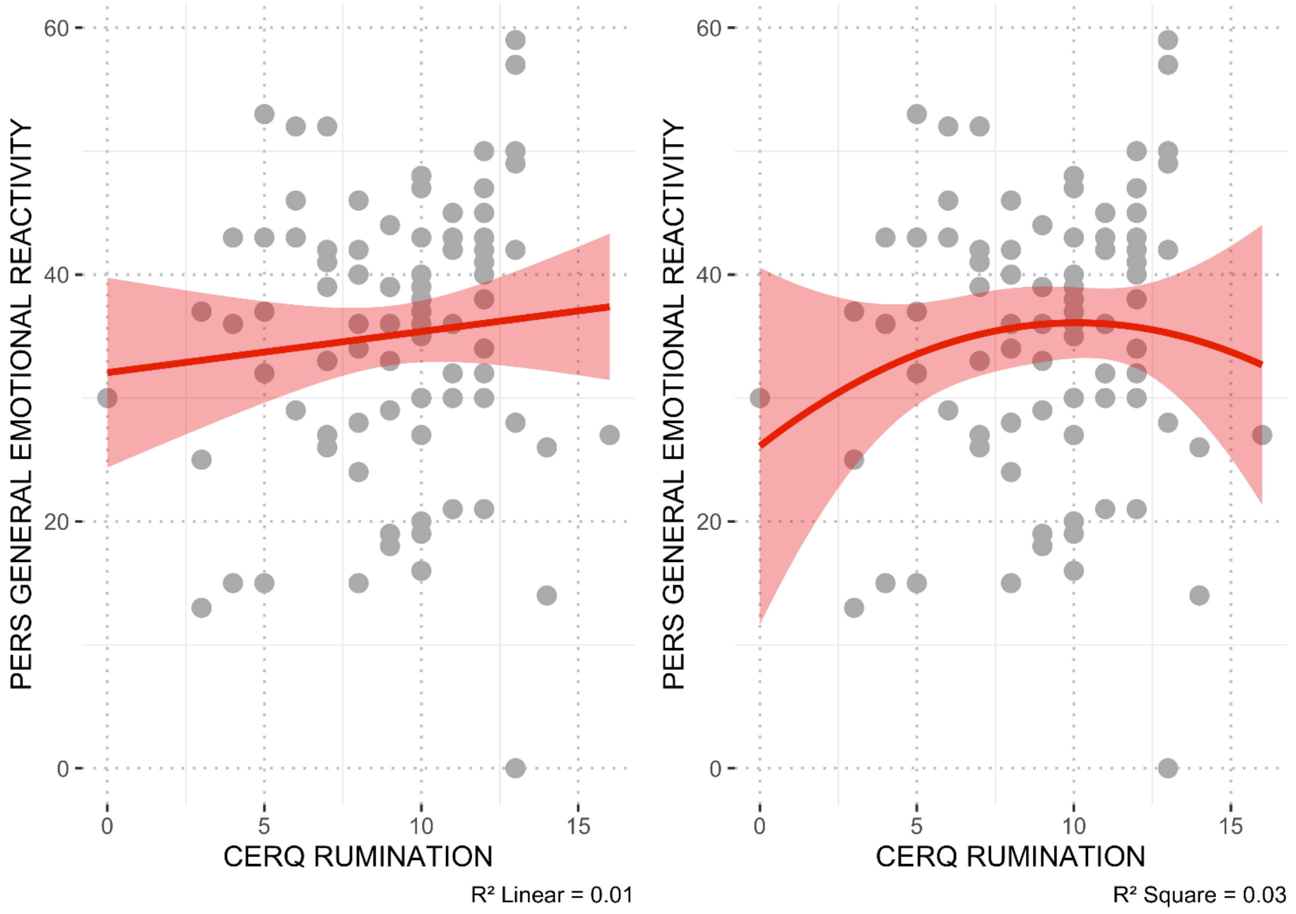

Figure 13

Relationship between AUDIT Score and PERS General Emotional Reactivity

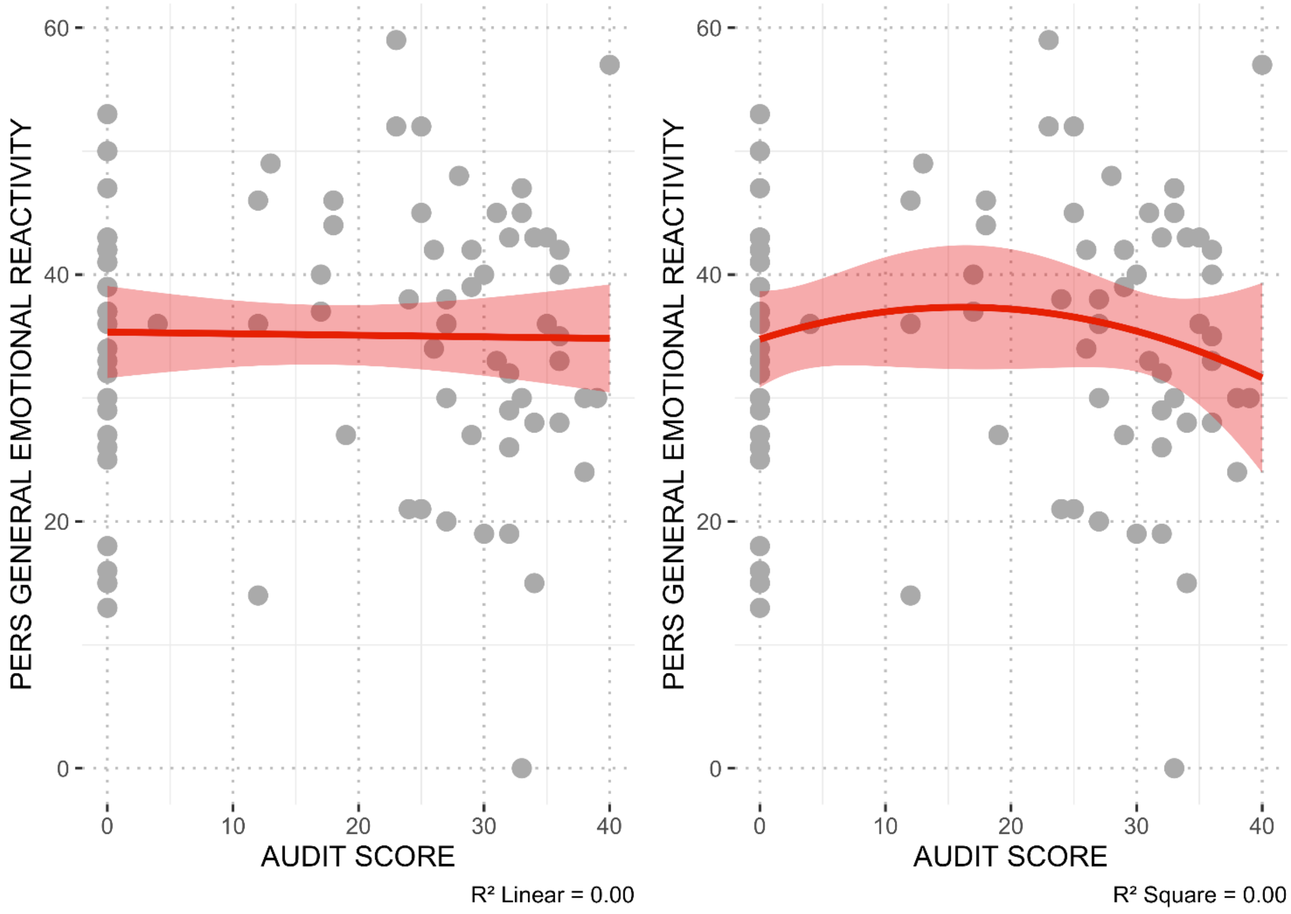

Supplement: Supplementary file 1 [file DataSheet1.pdf]
