# Supplementary material for: A moderated mediation model in assessing links between rumination, emotional reactivity, and suicidal risk in alcohol use disorder
Source: Front Psychiatry. 2025 Feb 28;16:1479827. doi: 10.3389/fpsyt.2025.1479827 (PMC11907195; doi:10.3389/fpsyt.2025.1479827)
Supplement: Supplementary file 2 [file DataSheet2.pdf]

Supplementary Material B

Influence Diagnostics and Moderation Analysis

1. Moderation Effect of the Variable GROUP on the Relationship Between the Variable SEX and the Variable SBQR SCORE

Figure 1a: Outlier observations in the relationship between the variable SEX and the variable SBQR SCORE within subsets of the variable GROUP (Note: id = row position in the dataset, different circle sizes represent varying Cook's distances, while the red gradient indicates the observed Cook's distance values)

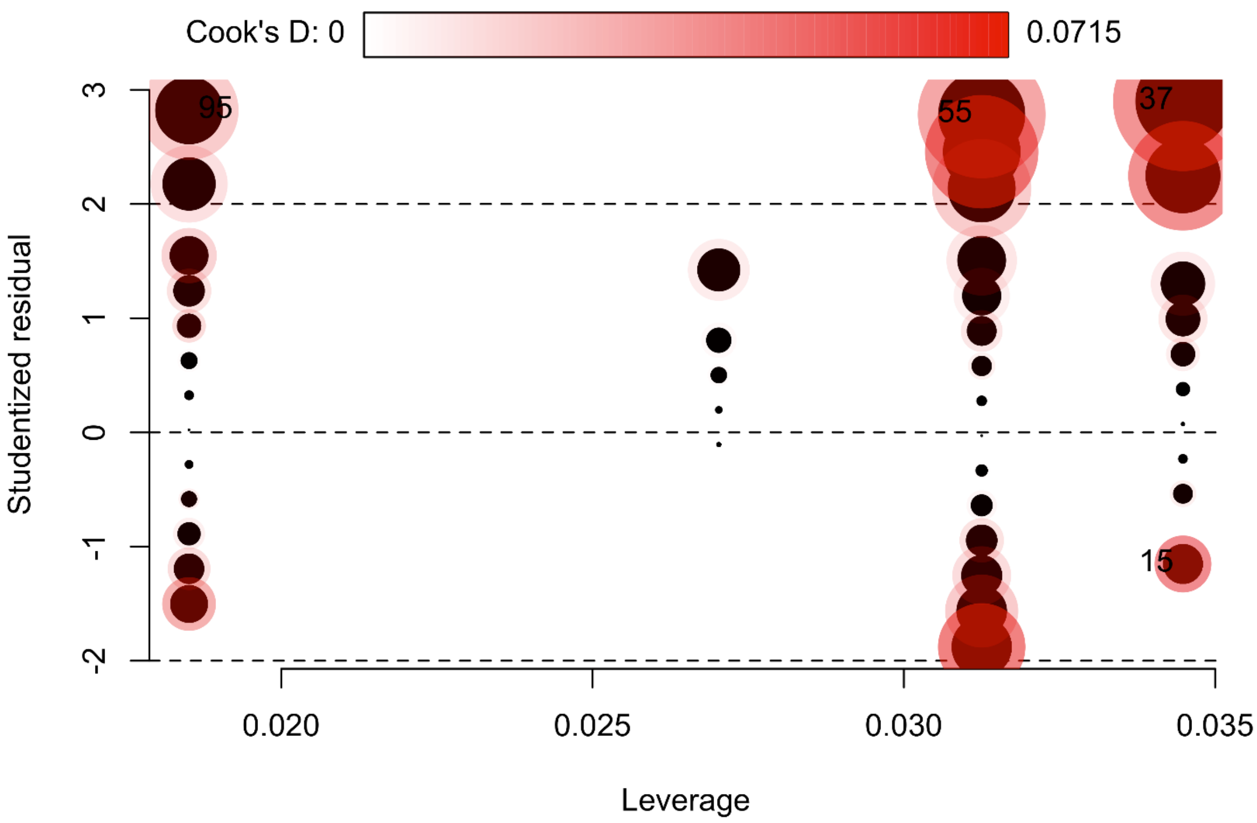

1.1. Analysis of the Effect on Raw Data

Figure 1b: Outlier observations in the relationship between the variable SEX and the variable SBQR SCORE within subsets of the variable GROUP (Note: id = row position in the dataset)

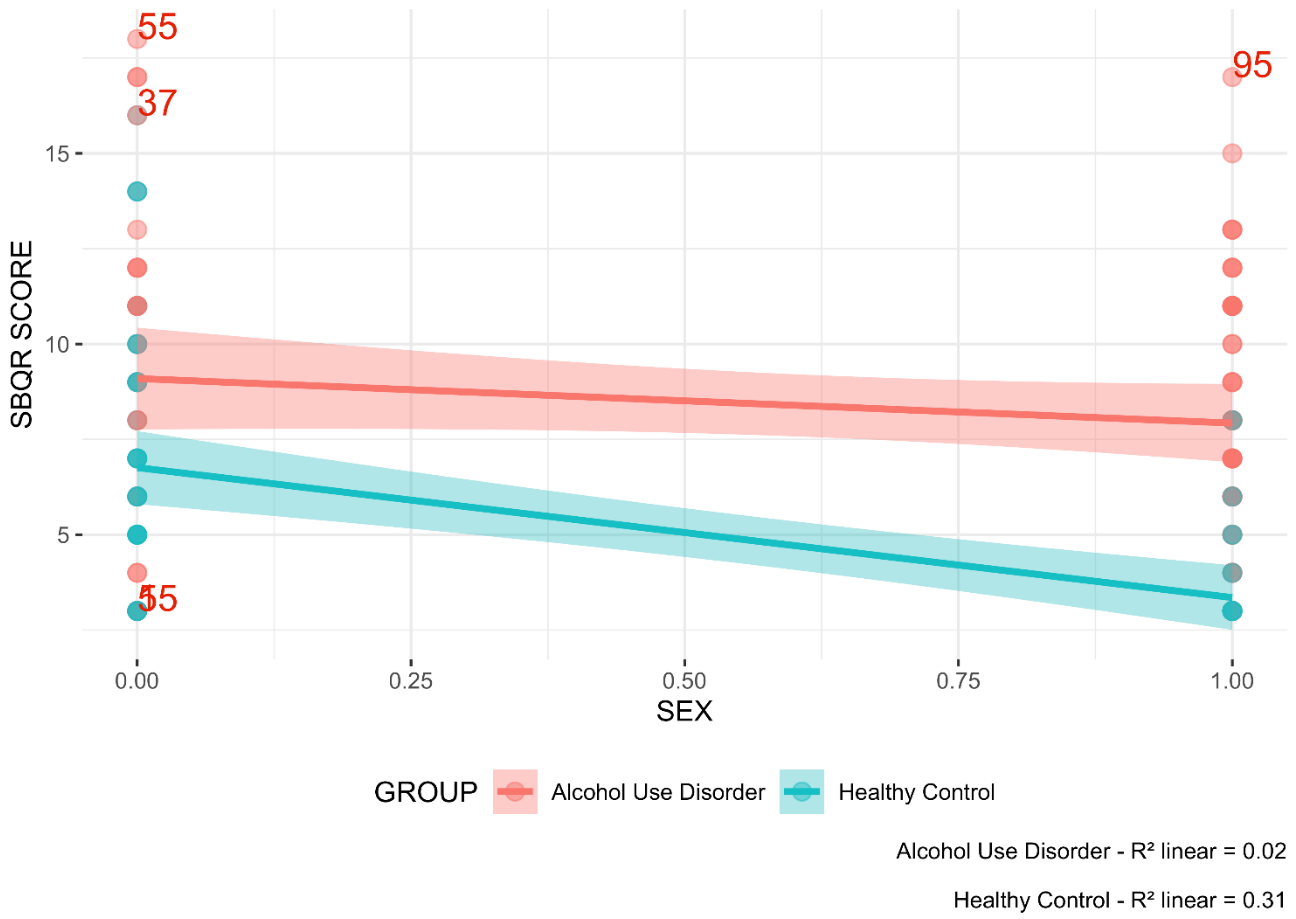

1.2. Analysis of the Effect on Smoothed Data

Figure 1c: Outlier observations in the relationship between the variable SEX and the variable SBQR SCORE within subsets of the variable GROUP (Note: id = row position in the dataset)

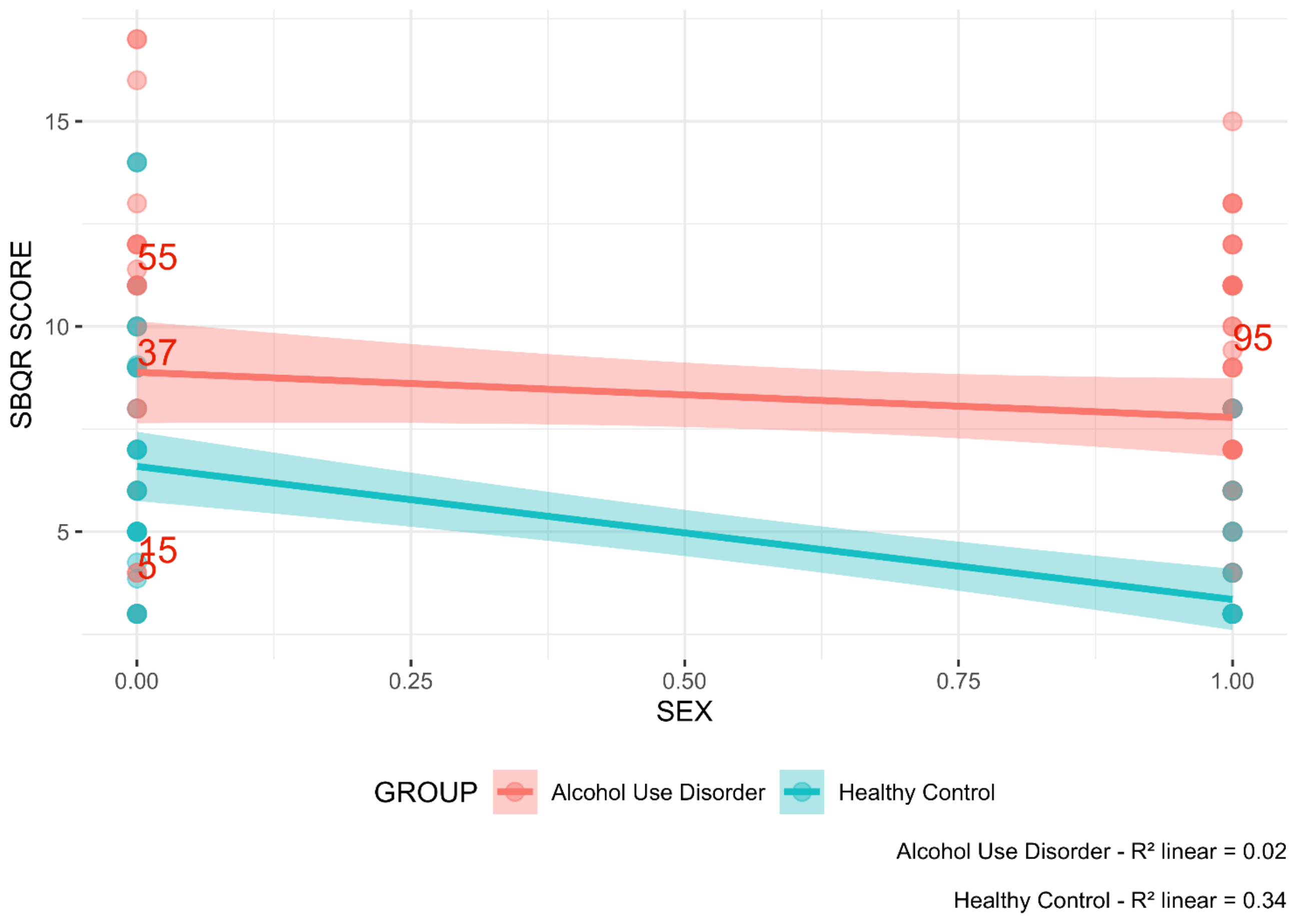

2. Moderation Effect of the Variable GROUP on the Relationship Between the Variable HADS DEPRESSION and the Variable SBQR SCORE

Figure 2a: Outlier observations in the relationship between the variable HADS DEPRESSION and the variable SBQR SCORE within subsets of the variable GROUP (Note: id = row position in the dataset, different circle sizes represent varying Cook's distances, while the red gradient indicates the observed Cook's distance values)

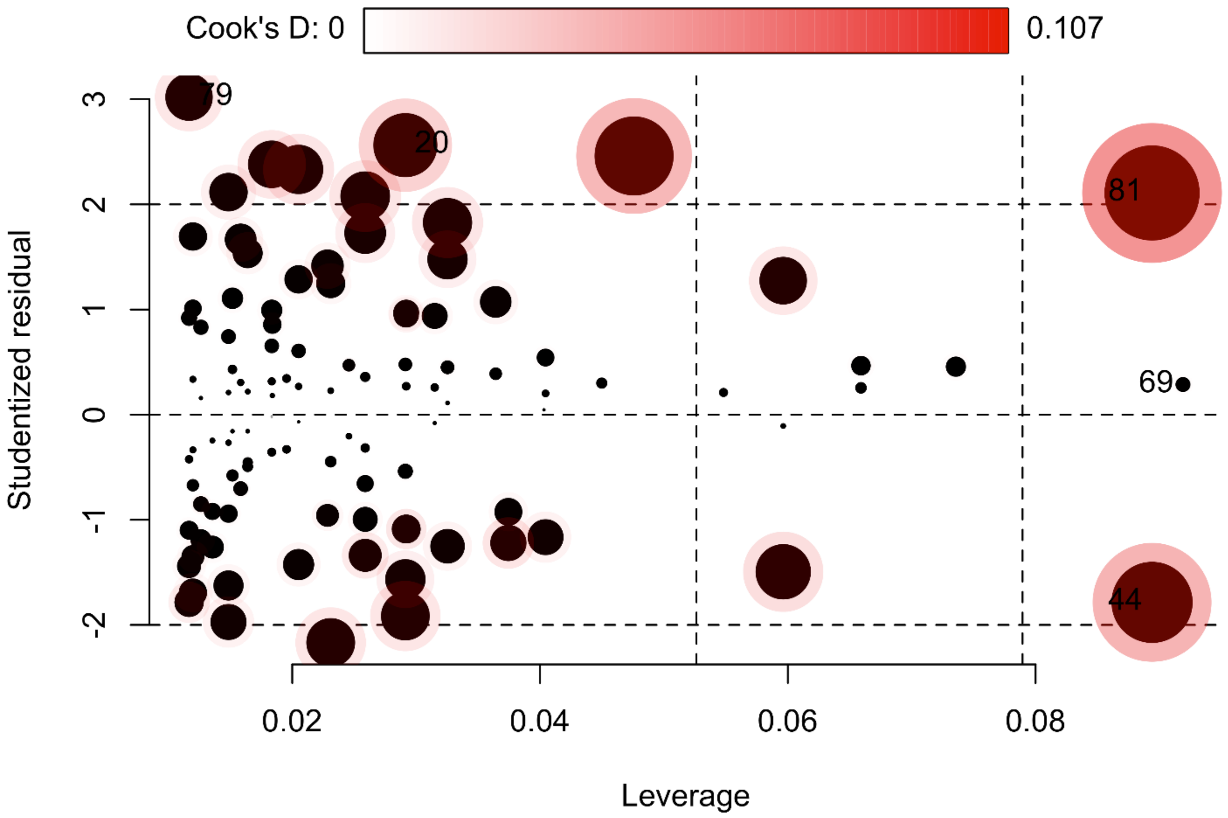

2.1. Analysis of the Effect on Raw Data

Figure 2b: Outlier observations in the relationship between the variable HADS DEPRESSION and the variable SBQR SCORE within subsets of the variable GROUP (Note: id = row position in the dataset)

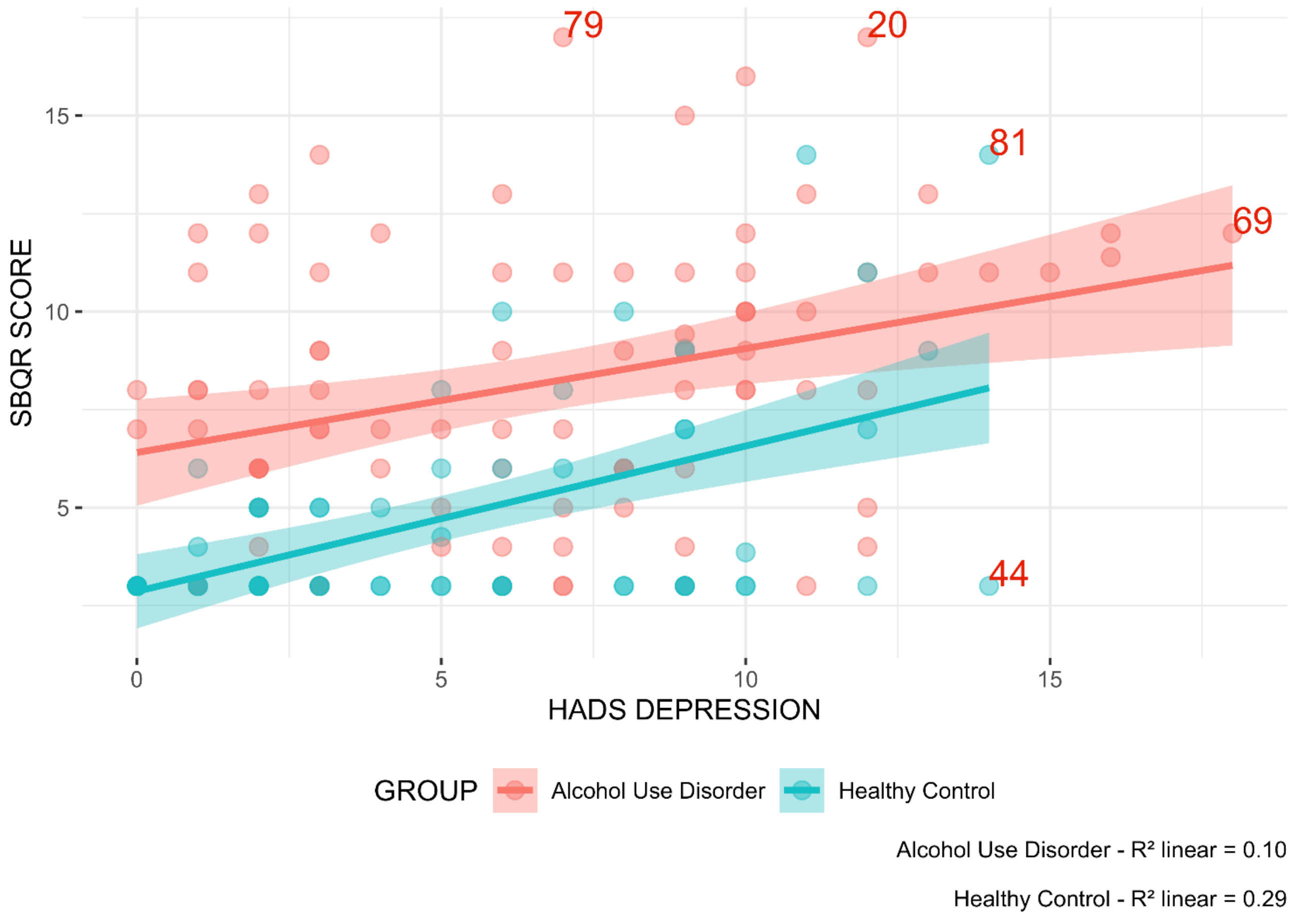

2.2. Analysis of the Effect on Smoothed Data

Figure 2c: Outlier observations in the relationship between the variable HADS DEPRESSION and the variable SBQR SCORE within subsets of the variable GROUP (Note: id = row position in the dataset)

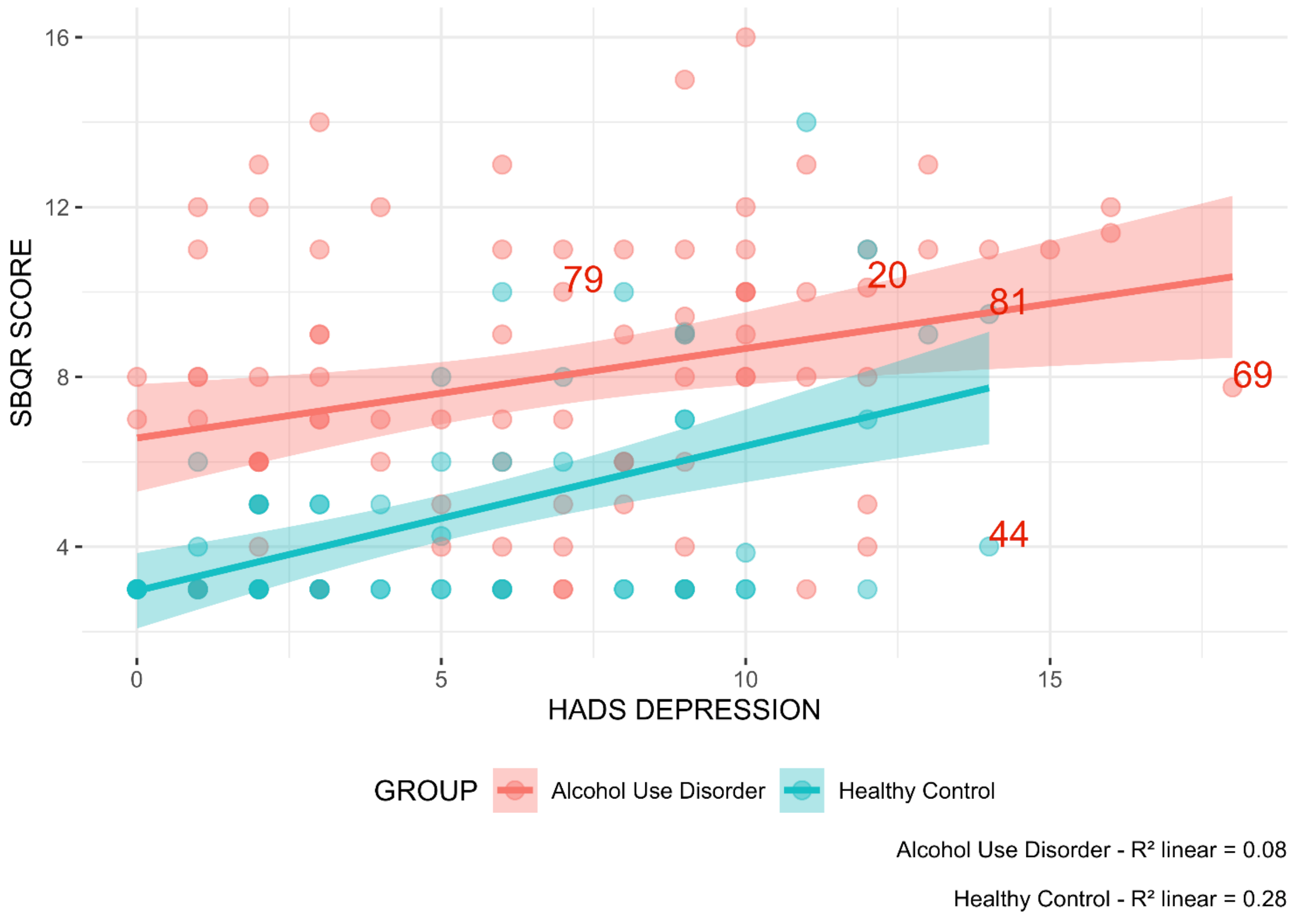

3. **Moderation Effect of the Variable GROUP on the Relationship Between the Variable AGE and the Variable SBQR SCORE**

Figure 3a: Outlier observations in the relationship between the variable AGE and the variable SBQR SCORE within subsets of the variable GROUP (Note: id = row position in the dataset, different circle sizes represent varying Cook's distances, while the red gradient indicates the observed Cook's distance values)

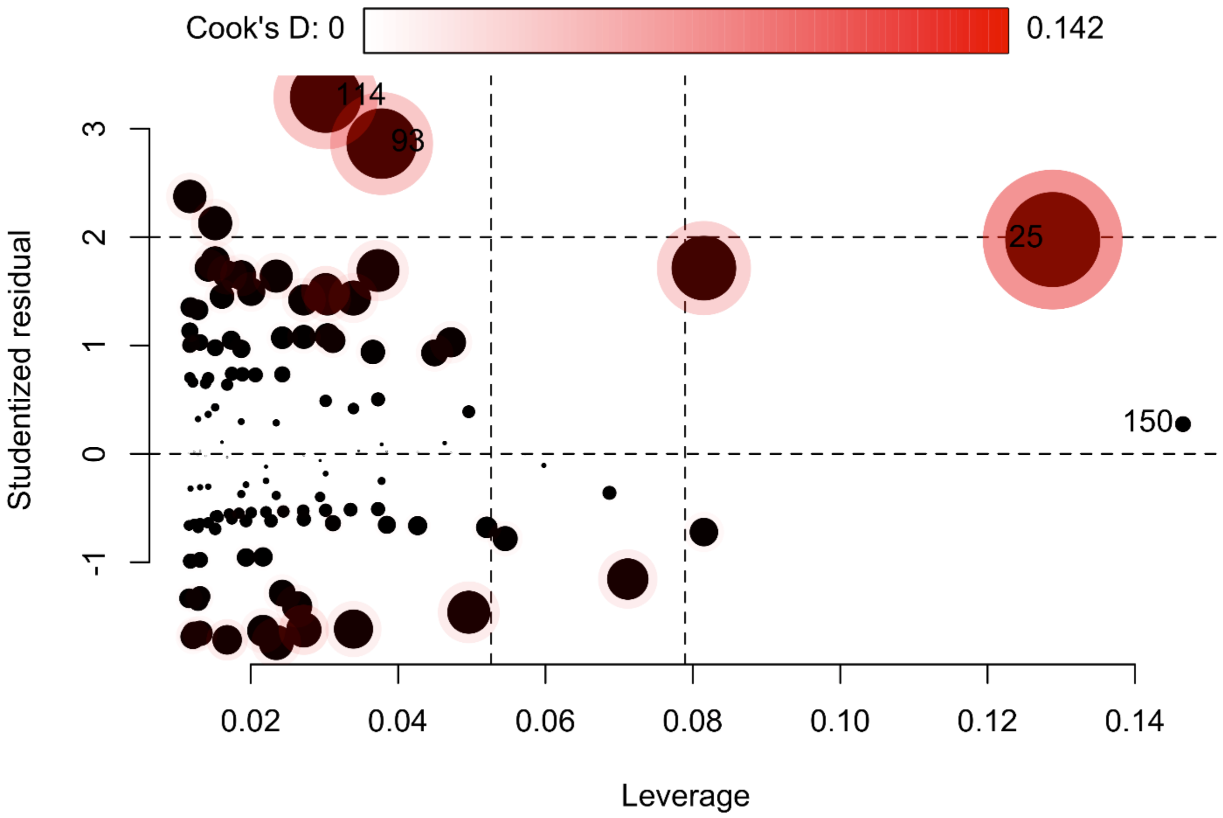

3.1. Analysis of the Effect on Raw Data

Figure 3b: Outlier observations in the relationship between the variable AGE and the variable SBQR SCORE within subsets of the variable GROUP (Note: id = row position in the dataset)

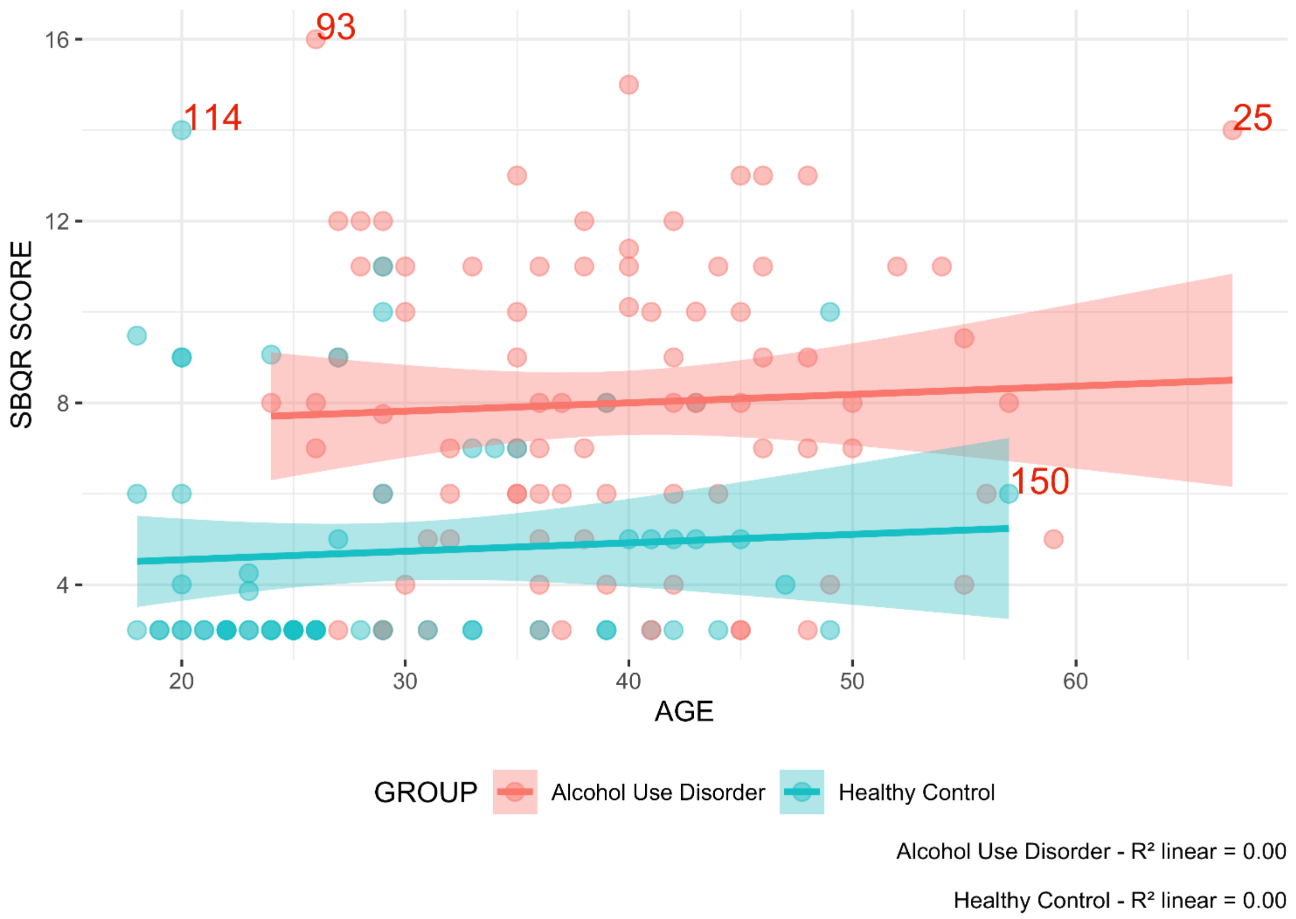

3.2. Analysis of the Effect on Smoothed Data

Figure 3c: Outlier observations in the relationship between the variable AGE and the variable SBQR SCORE within subsets of the variable GROUP (Note: id = row position in the dataset)

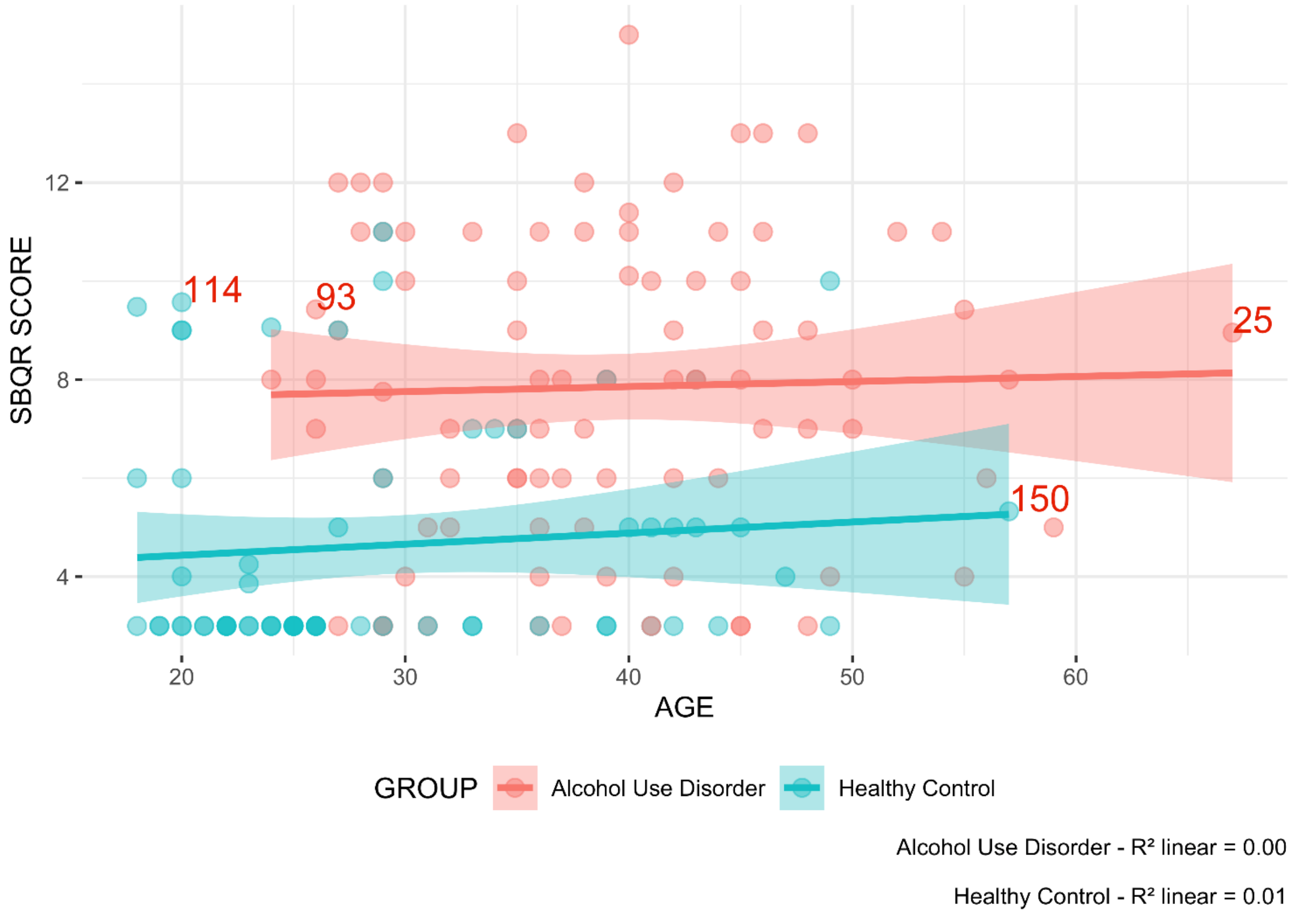

4. Moderation Effect of the Variable GROUP on the Relationship Between the Variable AUDIT SCORE and the Variable SBQR SCORE

Figure 4a: Outlier observations in the relationship between the variable AUDIT SCORE and the variable SBQR SCORE within subsets of the variable GROUP (Note: id = row position in the dataset, different circle sizes represent varying Cook's distances, while the red gradient indicates the observed Cook's distance values)

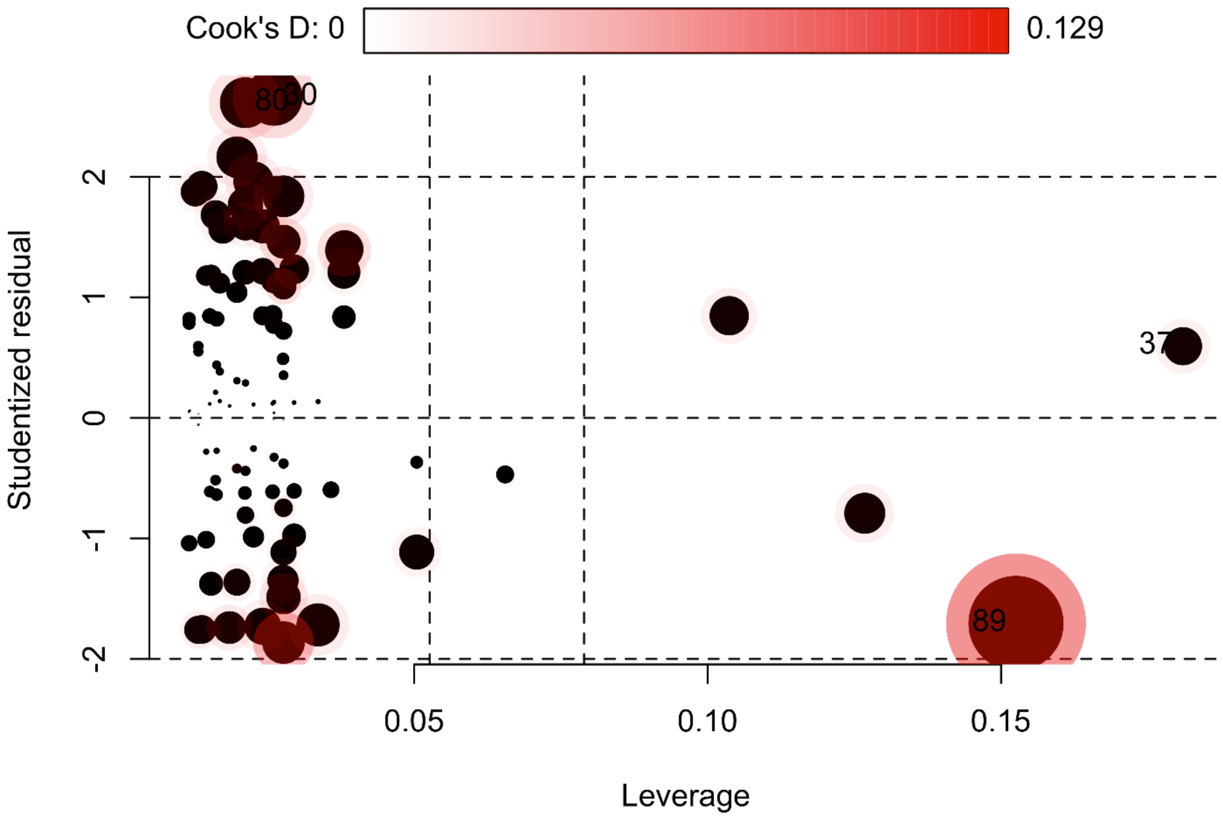

4.1. Analysis of the Effect on Raw Data

Figure 4b: Outlier observations in the relationship between the variable AUDIT SCORE and the variable SBQR SCORE within subsets of the variable GROUP (Note: id = row position in the dataset)

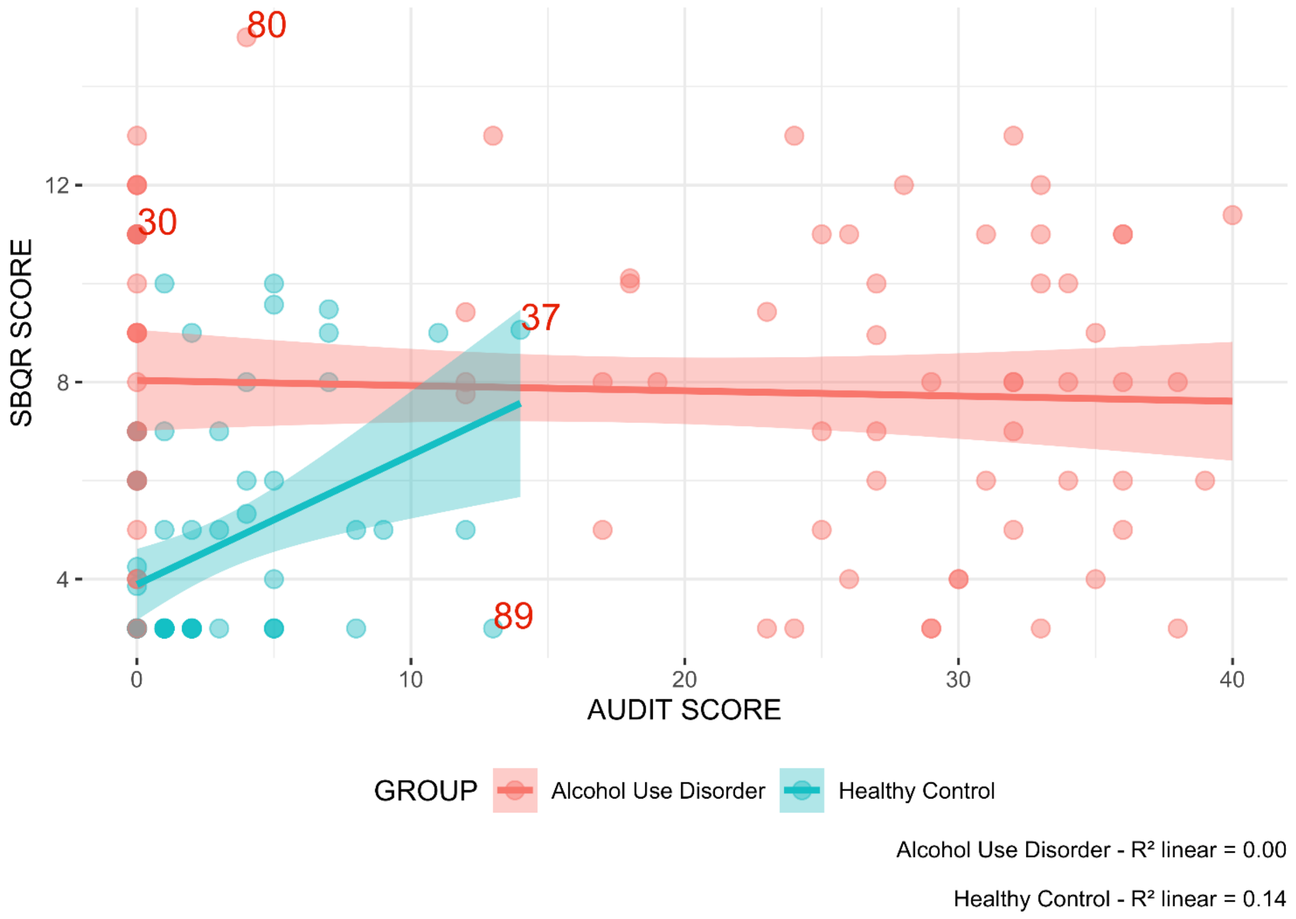

4.2. Analysis of the Effect on Smoothed Data

Figure 4c: Outlier observations in the relationship between the variable AUDIT SCORE and the variable SBQR SCORE within subsets of the variable GROUP (Note: id = row position in the dataset)

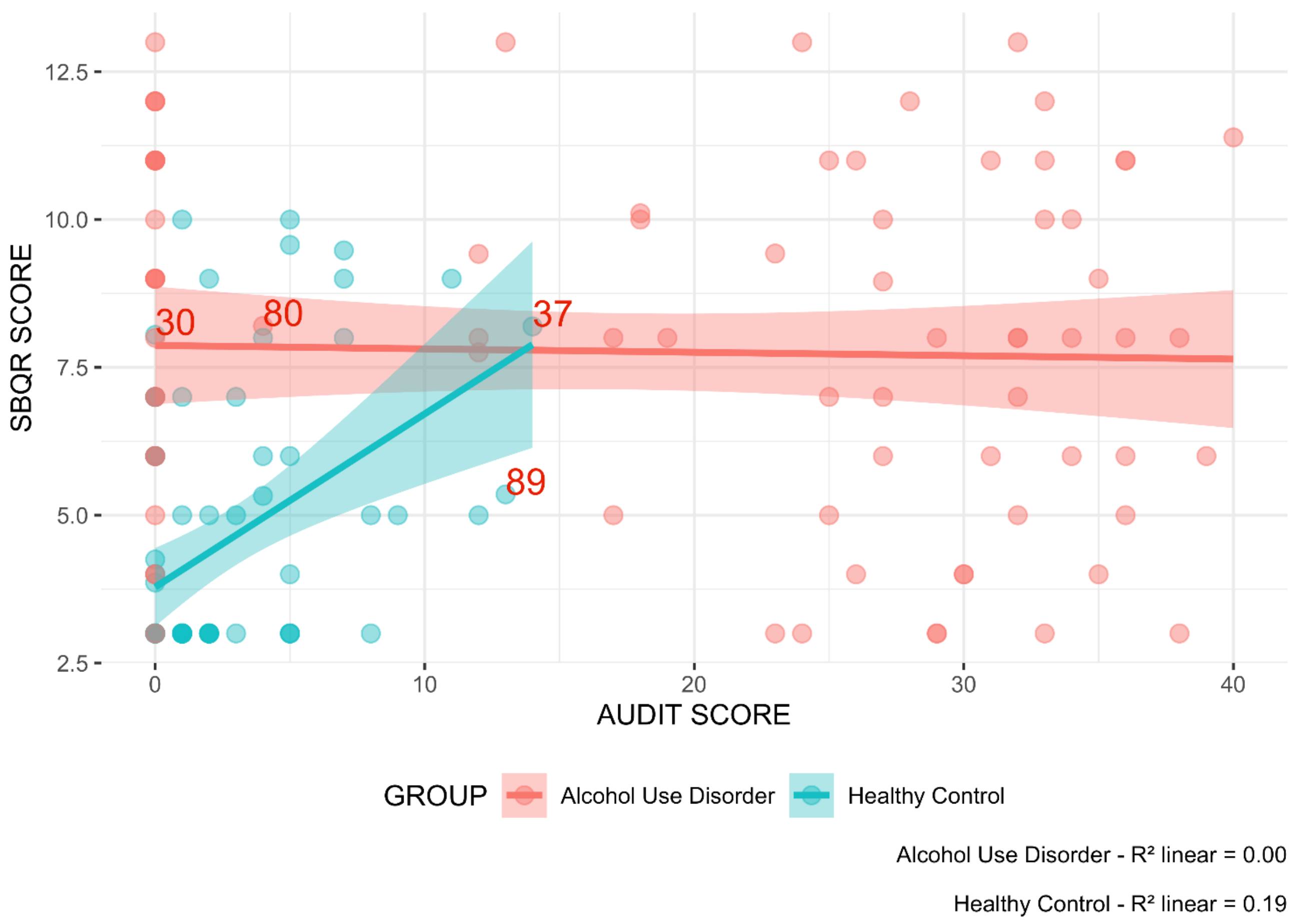

5. **Moderation Effect of the Variable GROUP on the Relationship Between the Variable PERS GENERAL EMOTIONAL REACTIVITY and the Variable SBQR SCORE**

Figure 5a: *Outlier observations in the relationship between the variable PERS GENERAL EMOTIONAL REACTIVITY and the variable SBQR SCORE within subsets of the variable GROUP (Note: id = row position in the dataset, different circle sizes represent varying Cook's distances, while the red gradient indicates the observed Cook's distance values)*

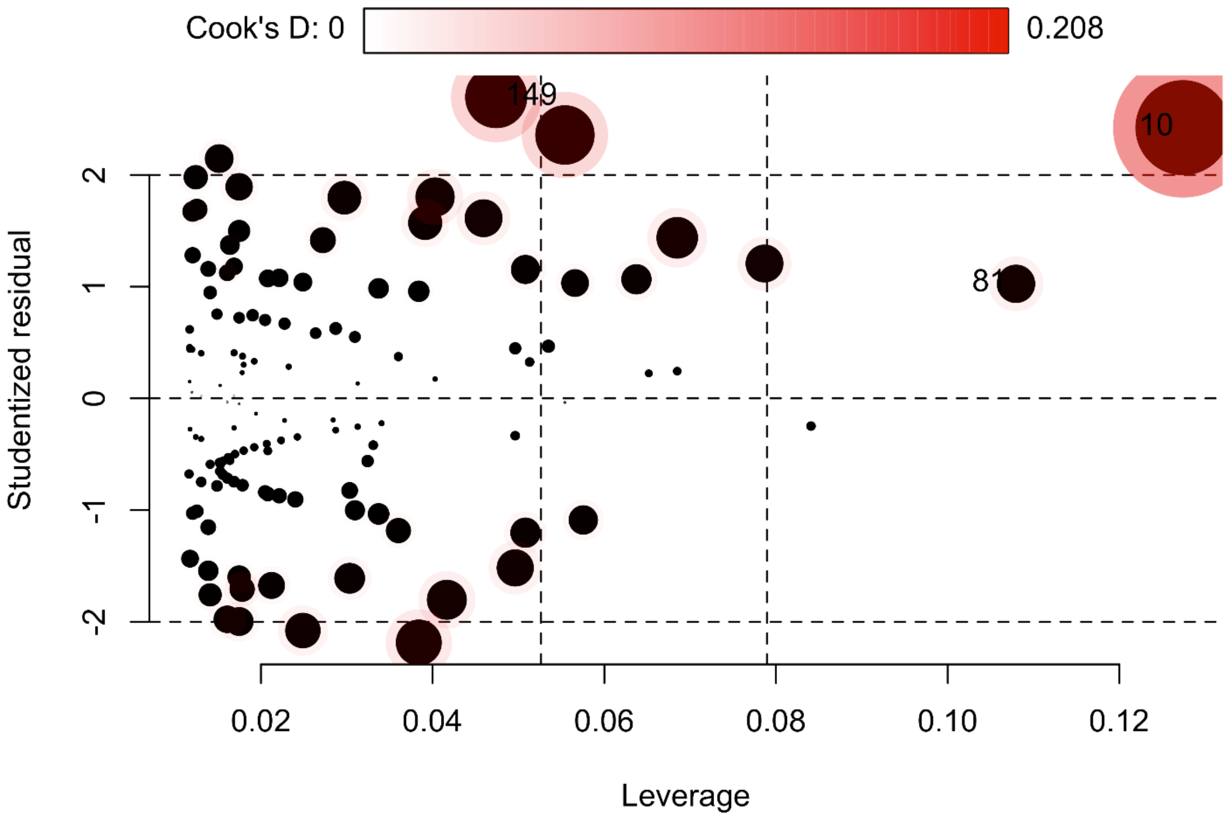

5.1. Analysis of the Effect on Raw Data

Figure 5b: Outlier observations in the relationship between the variable PERS GENERAL EMOTIONAL REACTIVITY and the variable SBQR SCORE within subsets of the variable GROUP (Note: id = row position in the dataset)

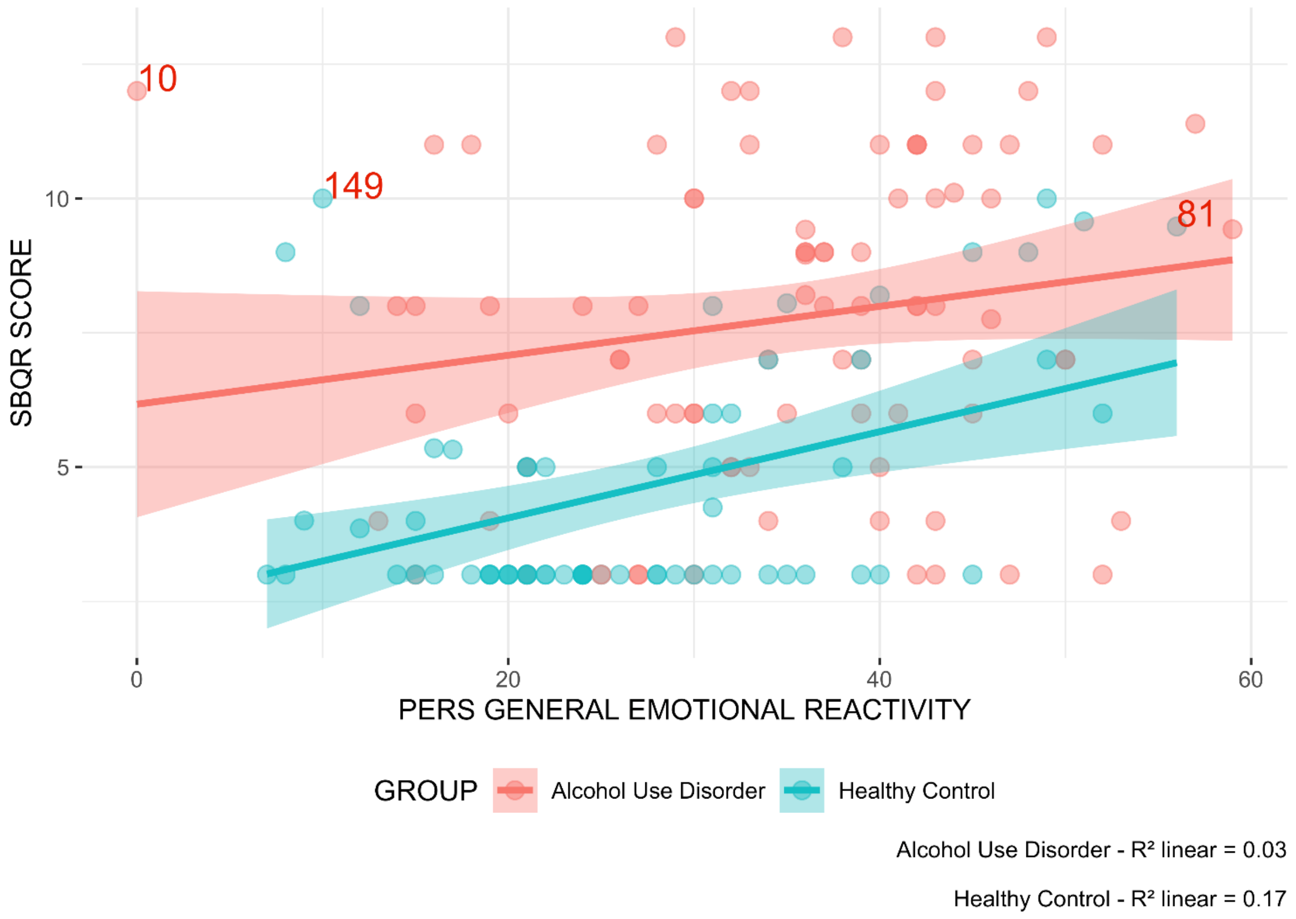

5.2. Analysis of the Effect on Smoothed Data

Figure 5c: Outlier observations in the relationship between the variable PERS GENERAL EMOTIONAL REACTIVITY and the variable SBQR SCORE within subsets of the variable GROUP (Note: id = row position in the dataset)

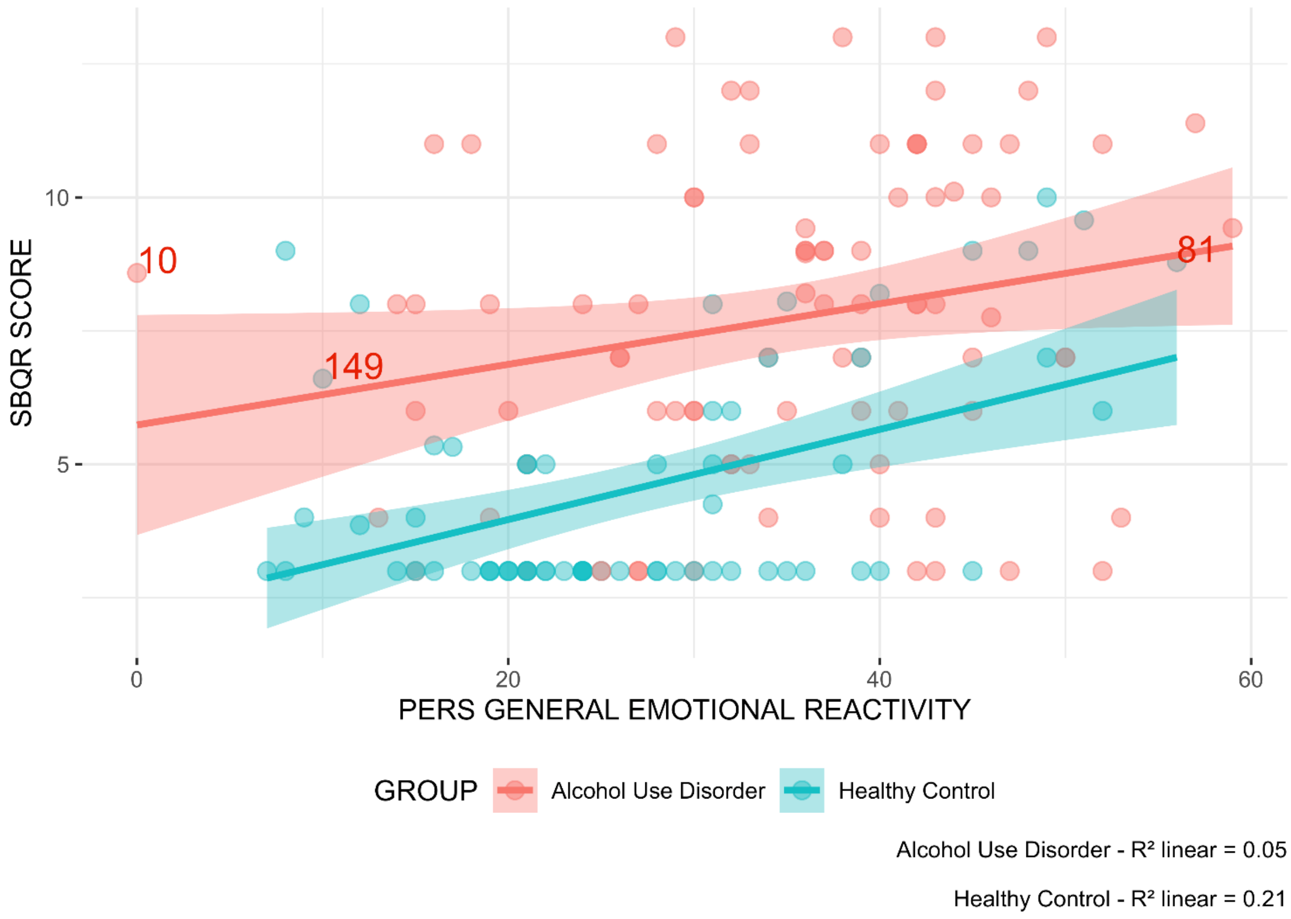

6. Moderation Effect of the Variable GROUP on the Relationship Between the Variable CERQ RUMINATION and the Variable SBQR SCORE

Figure 6a: Outlier observations in the relationship between the variable CERQ RUMINATION and the variable SBQR SCORE within subsets of the variable GROUP (Note: id = row position in the dataset, different circle sizes represent varying Cook's distances, while the red gradient indicates the observed Cook's distance values)

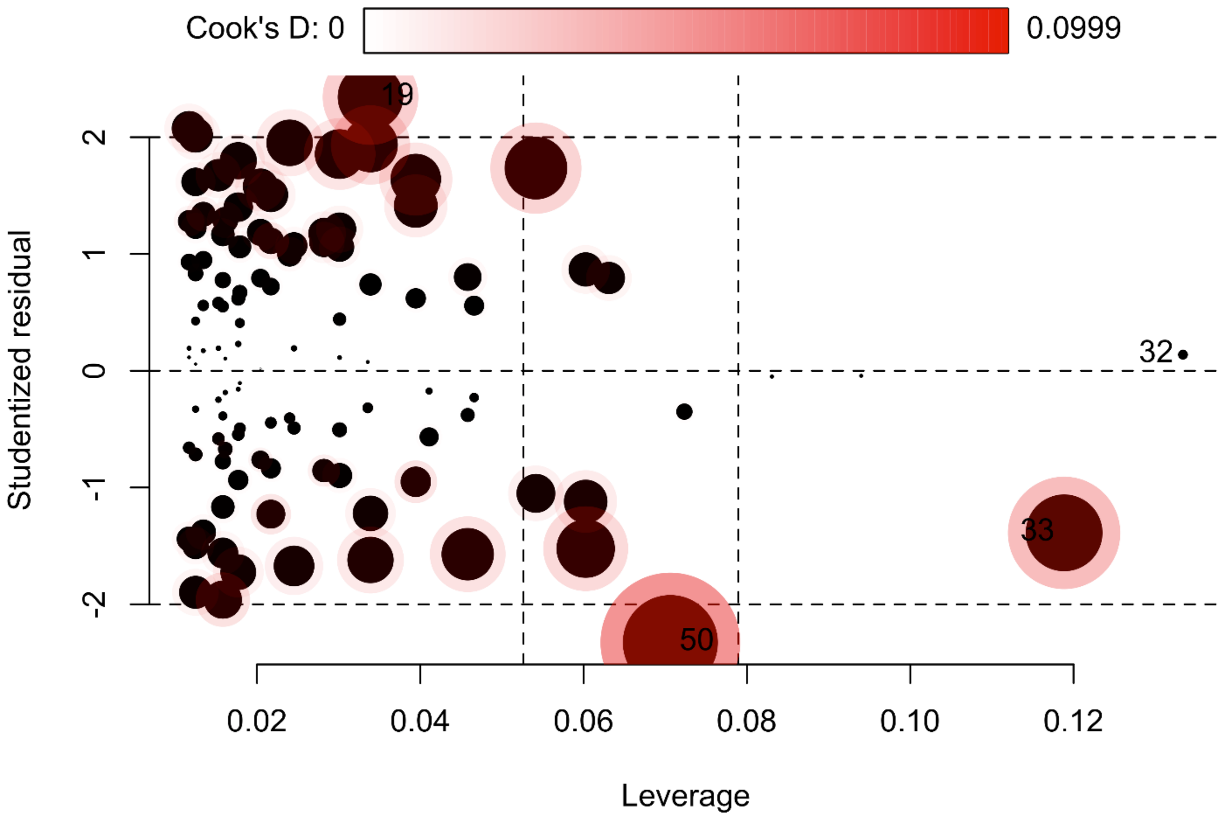

6.1. Analysis of the Effect on Raw Data

Figure 6b: Outlier observations in the relationship between the variable CERQ RUMINATION and the variable SBQR SCORE within subsets of the variable GROUP

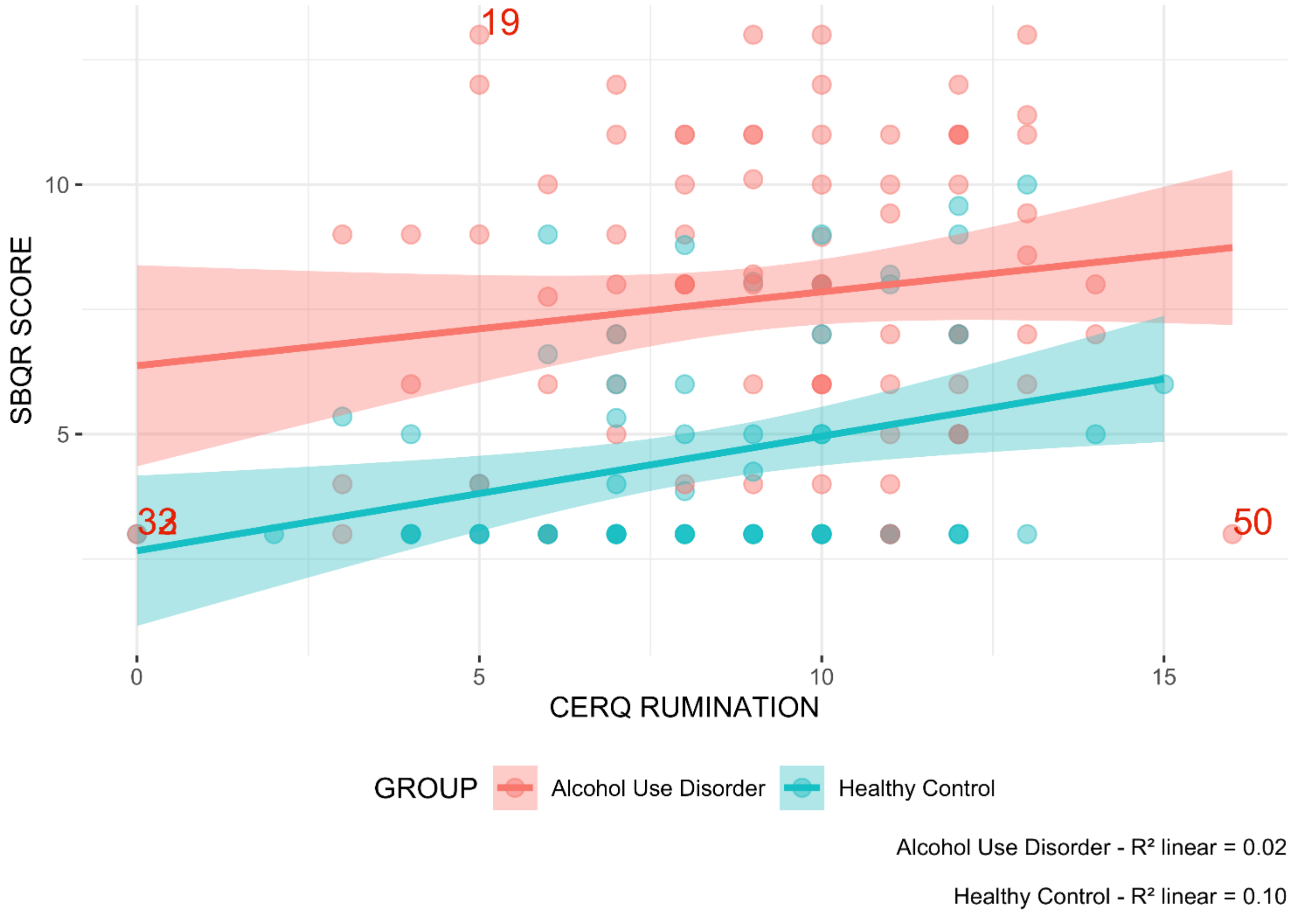

6.2. Analysis of the Effect on Smoothed Data

Figure 6c: Outlier observations in the relationship between the variable CERQ RUMINATION and the variable SBQR SCORE within subsets of the variable GROUP (Note: id = row position in the dataset)

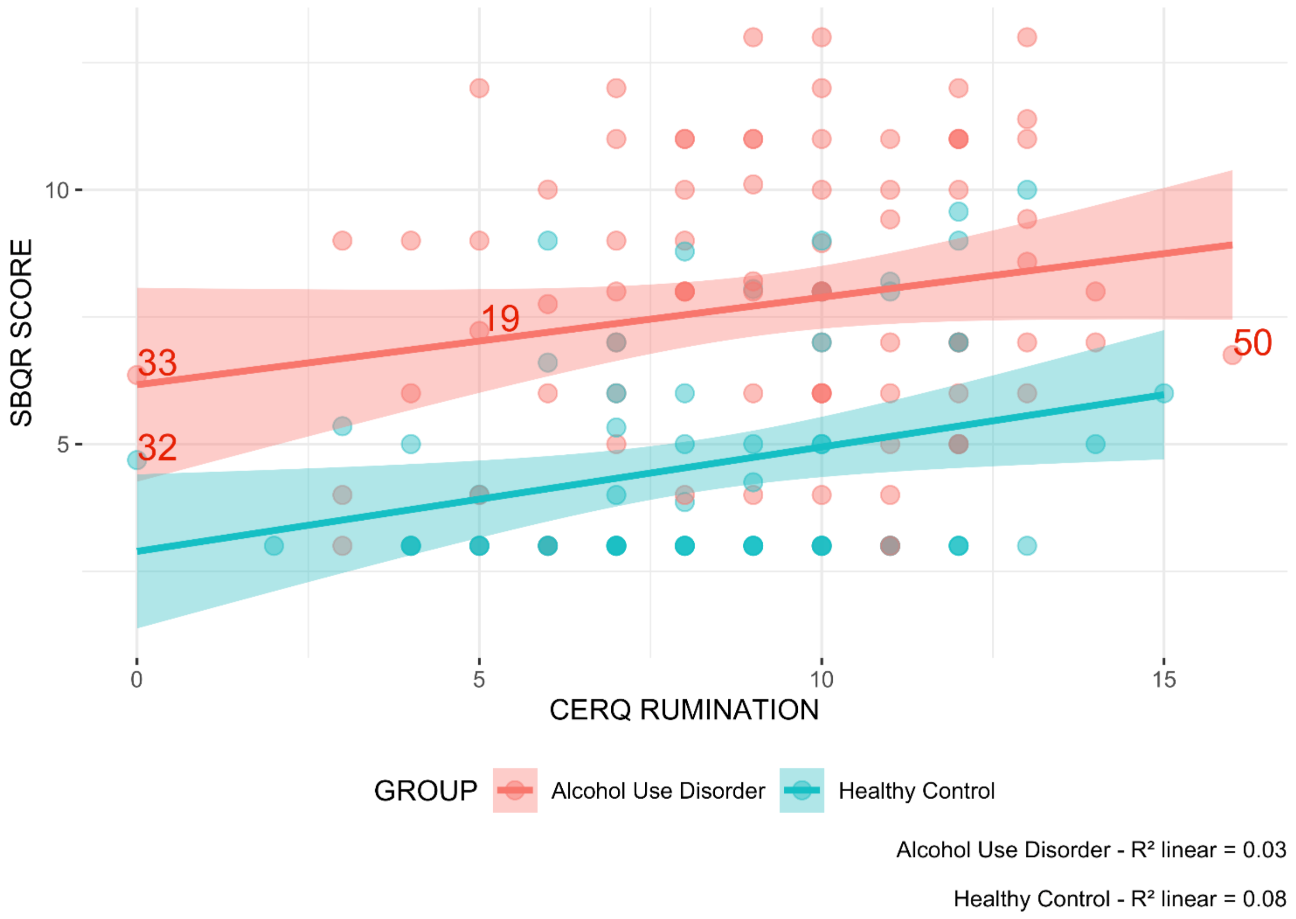

7. Moderation Effect of the Variable GROUP on the Relationship Between the Variable EDUCATION and the Variable SBQR SCORE

Figure 7a: Outlier observations in the relationship between the variable EDUCATION and the variable SBQR SCORE within subsets of the variable GROUP (Note: id = row position in the dataset, different circle sizes represent varying Cook's distances, while the red gradient indicates the observed Cook's distance values)

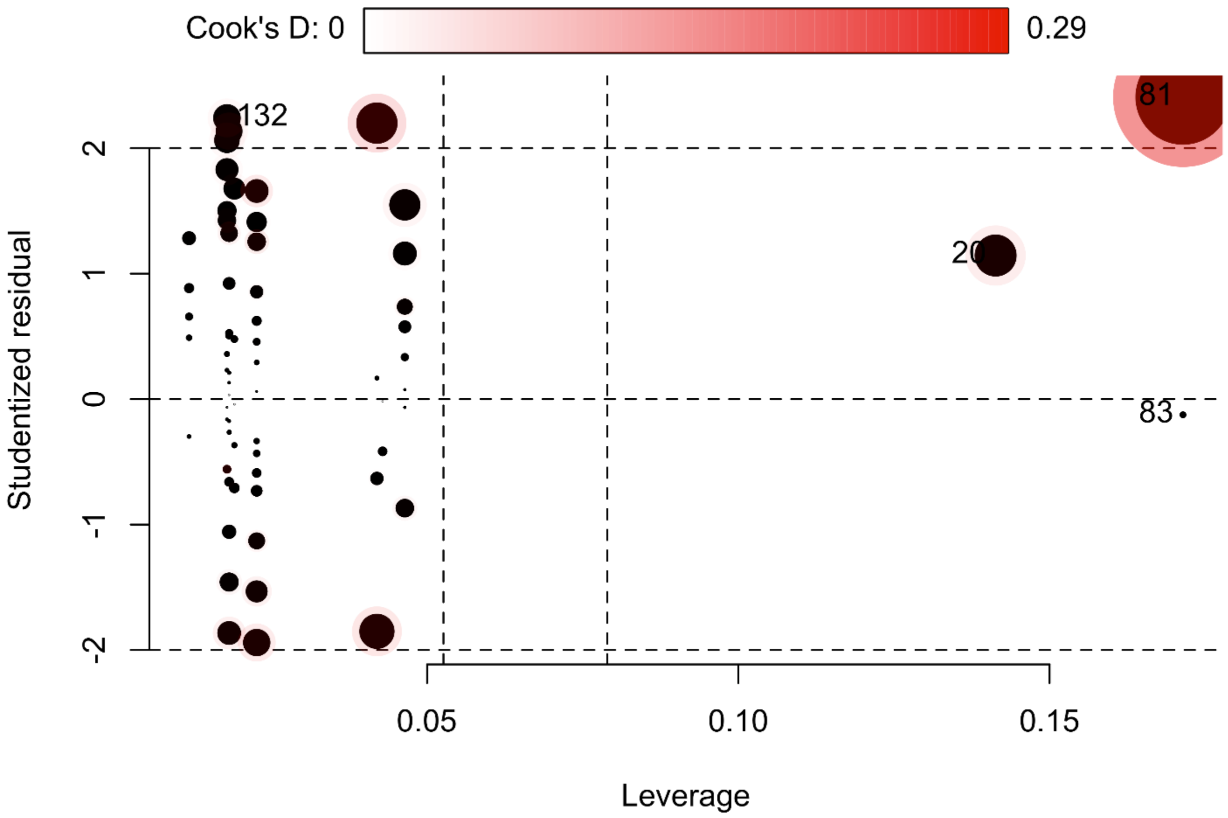

7.1. Analysis of the Effect on Raw Data

Figure 7b: Outlier observations in the relationship between the variable EDUCATION and the variable SBQR SCORE within subsets of the variable GROUP (Note: id = row position in the dataset)

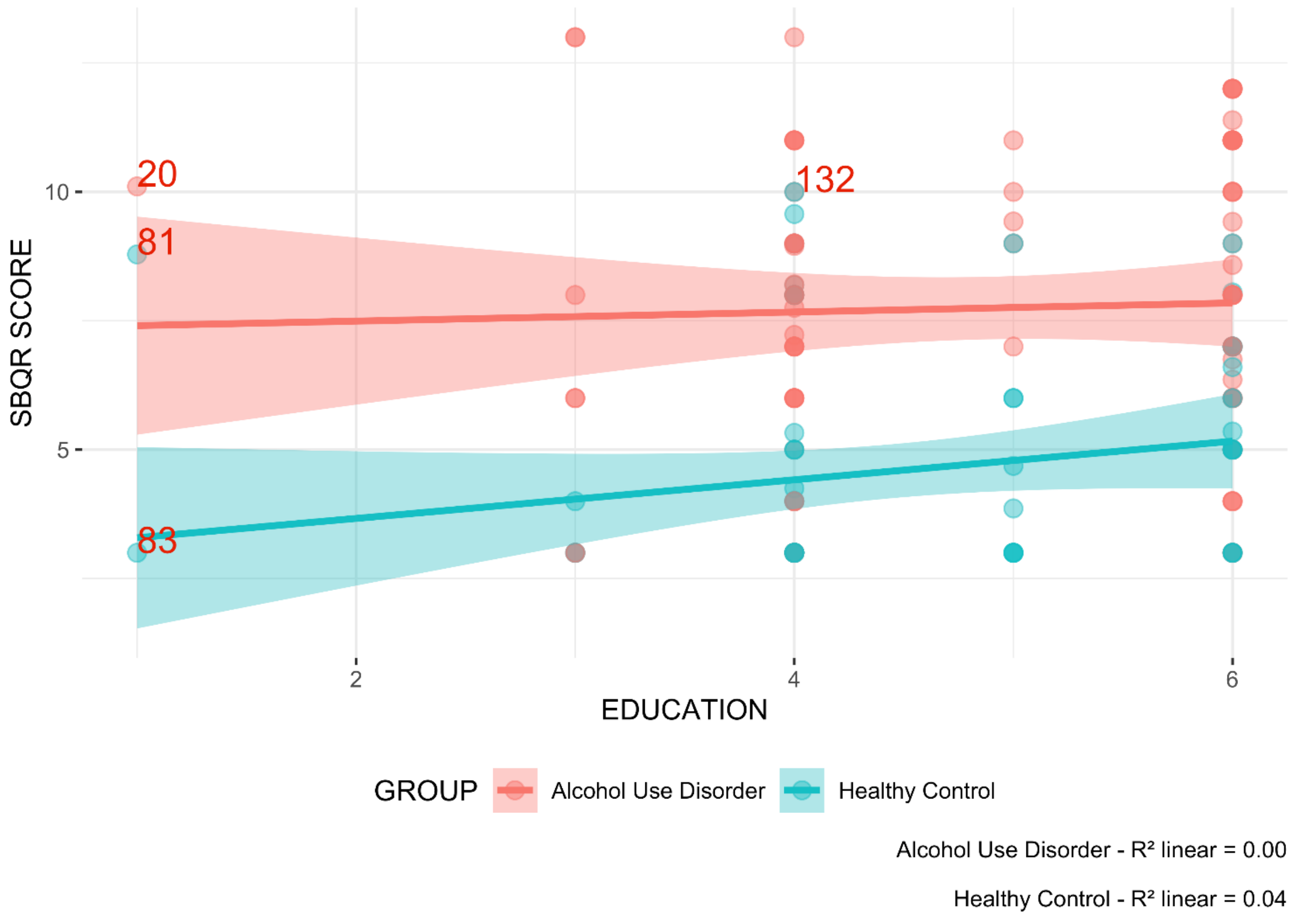

7.2. Analysis of the Effect on Smoothed Data

Figure 7c: Outlier observations in the relationship between the variable EDUCATION and the variable SBQR SCORE within subsets of the variable GROUP (Note: id = row position in the dataset)

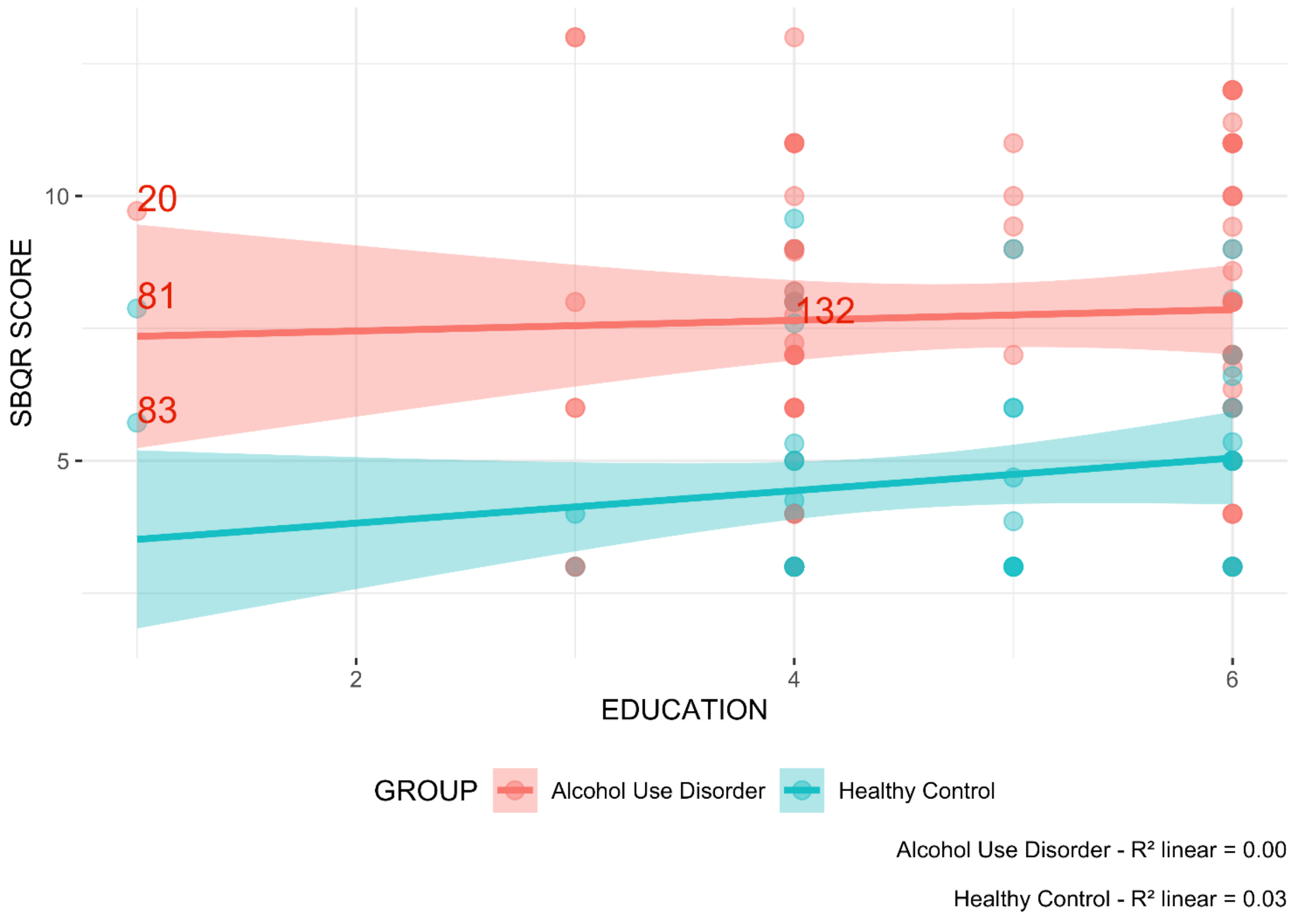

8. Summary of Outlier Observations in Moderated Regression Models

The row numbers (observations) in the dataset that appeared as outliers at least once in the analysis are as follows:

(5,15,37,55,95,20,44,69,79,81,25,93,114,150,30,80,89,10,149,19,32,33,50,83,132)
