# Supplementary material for: A moderated mediation model in assessing links between rumination, emotional reactivity, and suicidal risk in alcohol use disorder
Source: Front Psychiatry. 2025 Feb 28;16:1479827. doi: 10.3389/fpsyt.2025.1479827 (PMC11907195; doi:10.3389/fpsyt.2025.1479827)
Supplement: Supplementary file 3 [file Table1.docx]

Supplementary Material C

# Table 1 *Estimates of Correlations Between Components in the Model*

| Variables 1 | Variables 2 | *r* | *p* |
| --- | --- | --- | --- |
| HADS Depression | AUDIT Score | 0.23 | 0.006 |
| HADS Depression | SEX | -0.23 | 0.004 |
| HADS Depression | AGE | -0.13 | 0.083 |
| HADS Depression | EDUCATION | 0.10 | 0.262 |
| AUDIT Score | SEX | -0.13 | 0.099 |
| AUDIT Score | AGE | 0.22 | 0.003 |
| AUDIT Score | EDUCATION | 0.17 | 0.025 |
| SEX | AGE | 0.04 | 0.612 |
| SEX | EDUCATION | -0.10 | 0.207 |
| AGE | EDUCATION | 0.11 | 0.159 |
| CERQ Rumination | HADS Depression | 0.21 | 0.007 |
| CERQ Rumination | AUDIT Score | 0.25 | 0.001 |
| CERQ Rumination | SEX | -0.13 | 0.105 |
| CERQ Rumination | AGE | 0.00 | 0.974 |
| CERQ Rumination | EDUCATION | 0.08 | NA |
